# Supplementary figures and images for: Insufficiency of 40S ribosomal proteins, RPS26 and RPS25, negatively affects biosynthesis of polyglycine-containing proteins in fragile-X associated conditions
Source: eLife. 2025 May 16;13:RP98631. doi: 10.7554/eLife.98631 (PMC12084008; doi:10.7554/eLife.98631)

Images corresponding to **Figure 1:**

**
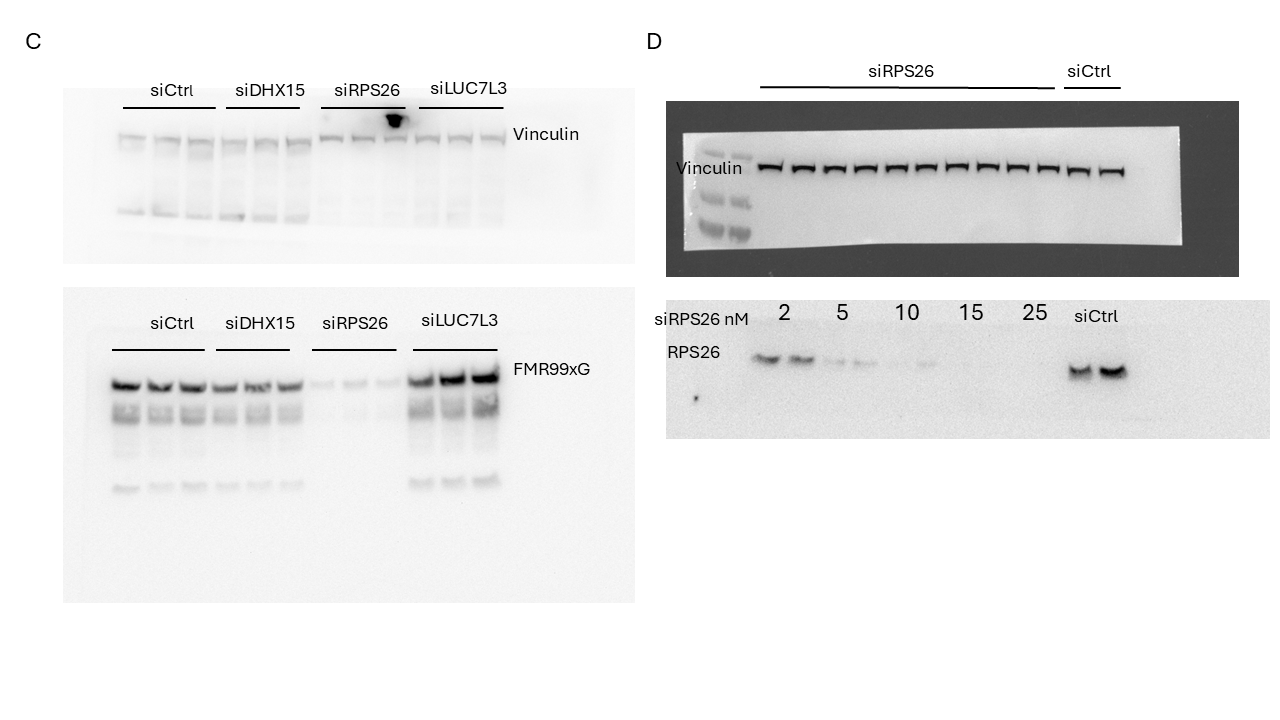
**

Supplement: Figure 1—source data 1. [file elife-98631-fig1-data1.zip › Figure 1 - source data 1 .docx]

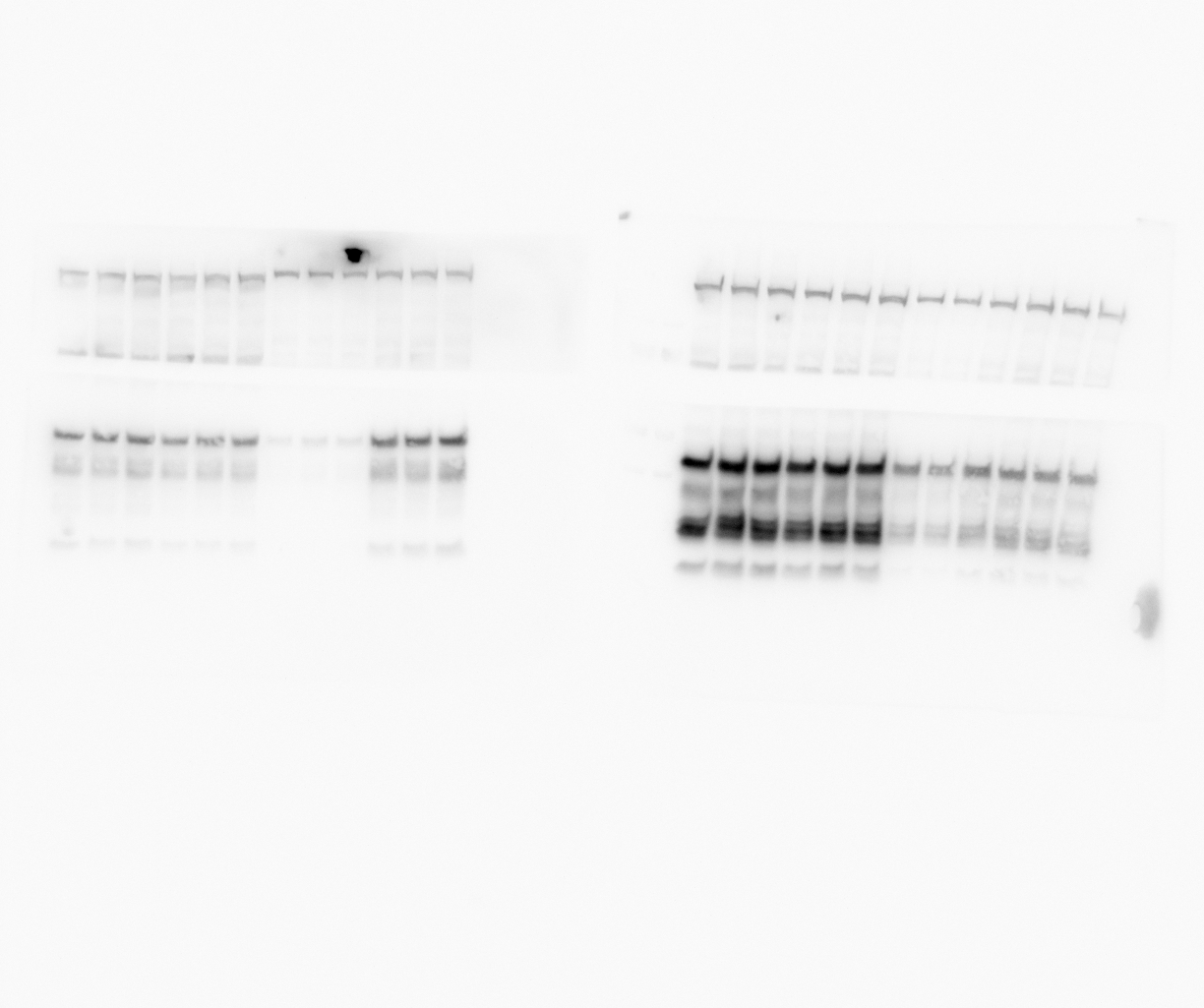

Supplement: Figure 1—source data 2. [file elife-98631-fig1-data2.zip › 1C FMR99xG with vinculin.tif]

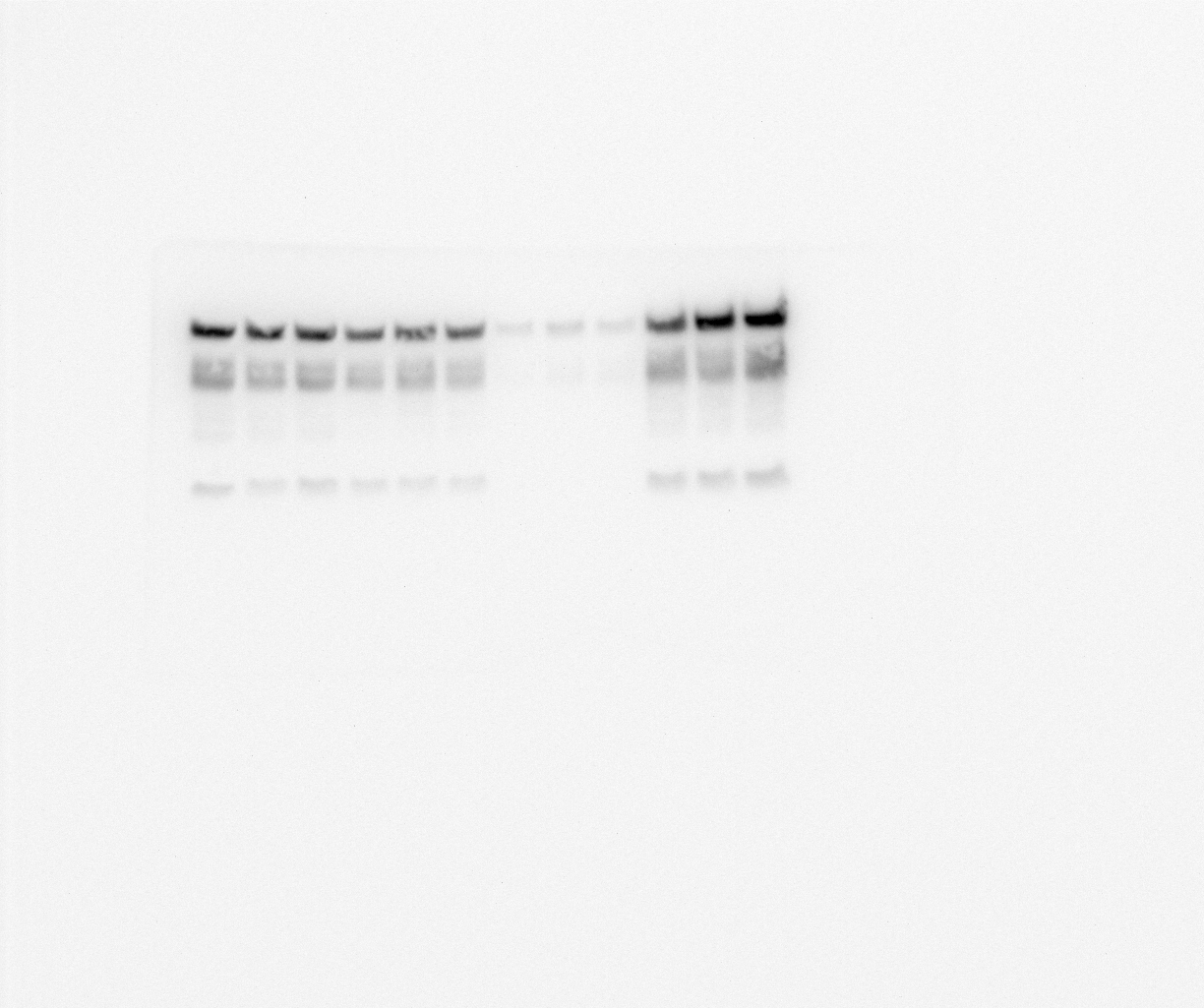

Supplement: Figure 1—source data 2. [file elife-98631-fig1-data2.zip › 1C FMR99xG .tif]

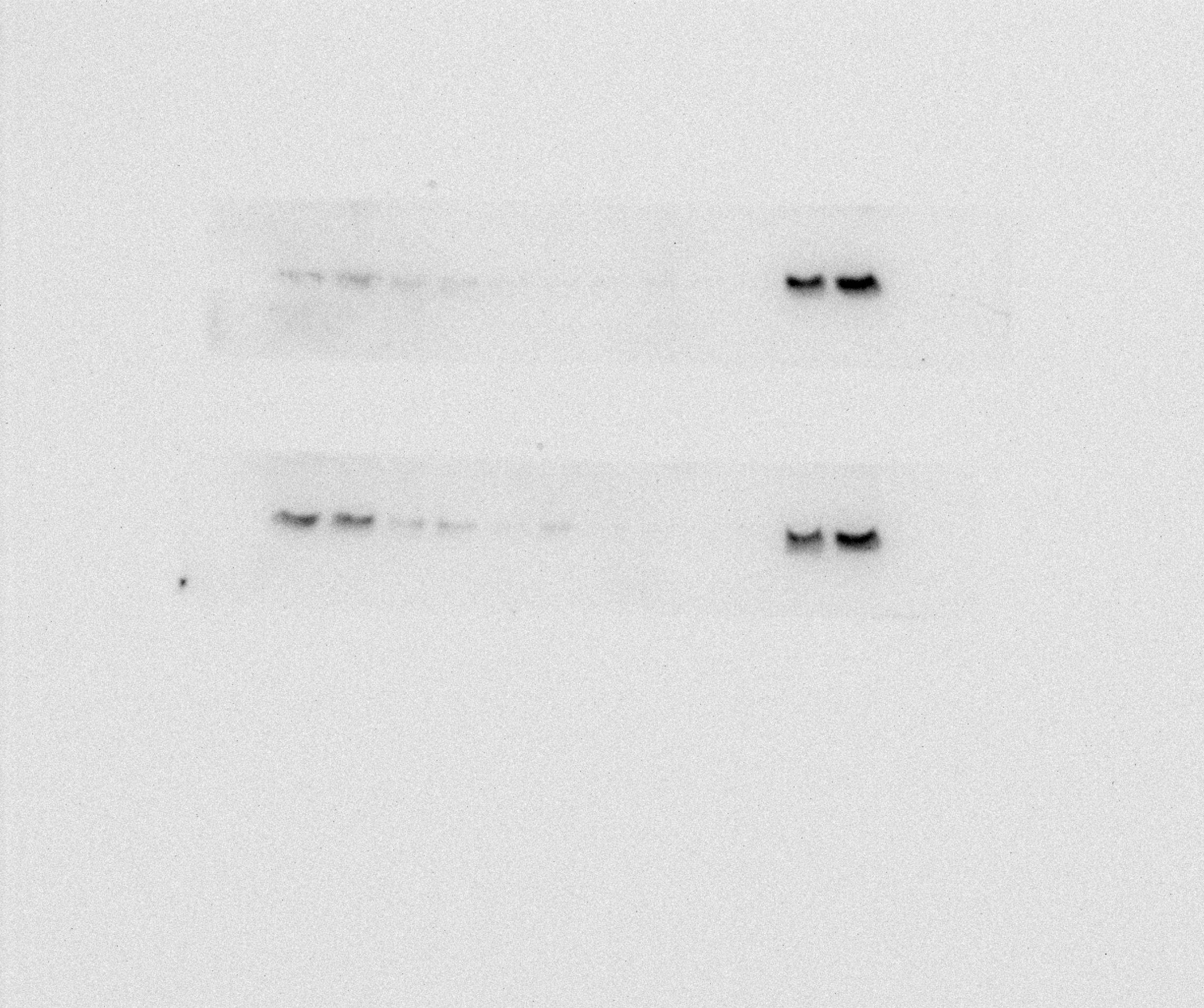

Supplement: Figure 1—source data 2. [file elife-98631-fig1-data2.zip › 1D RPS26.tif]

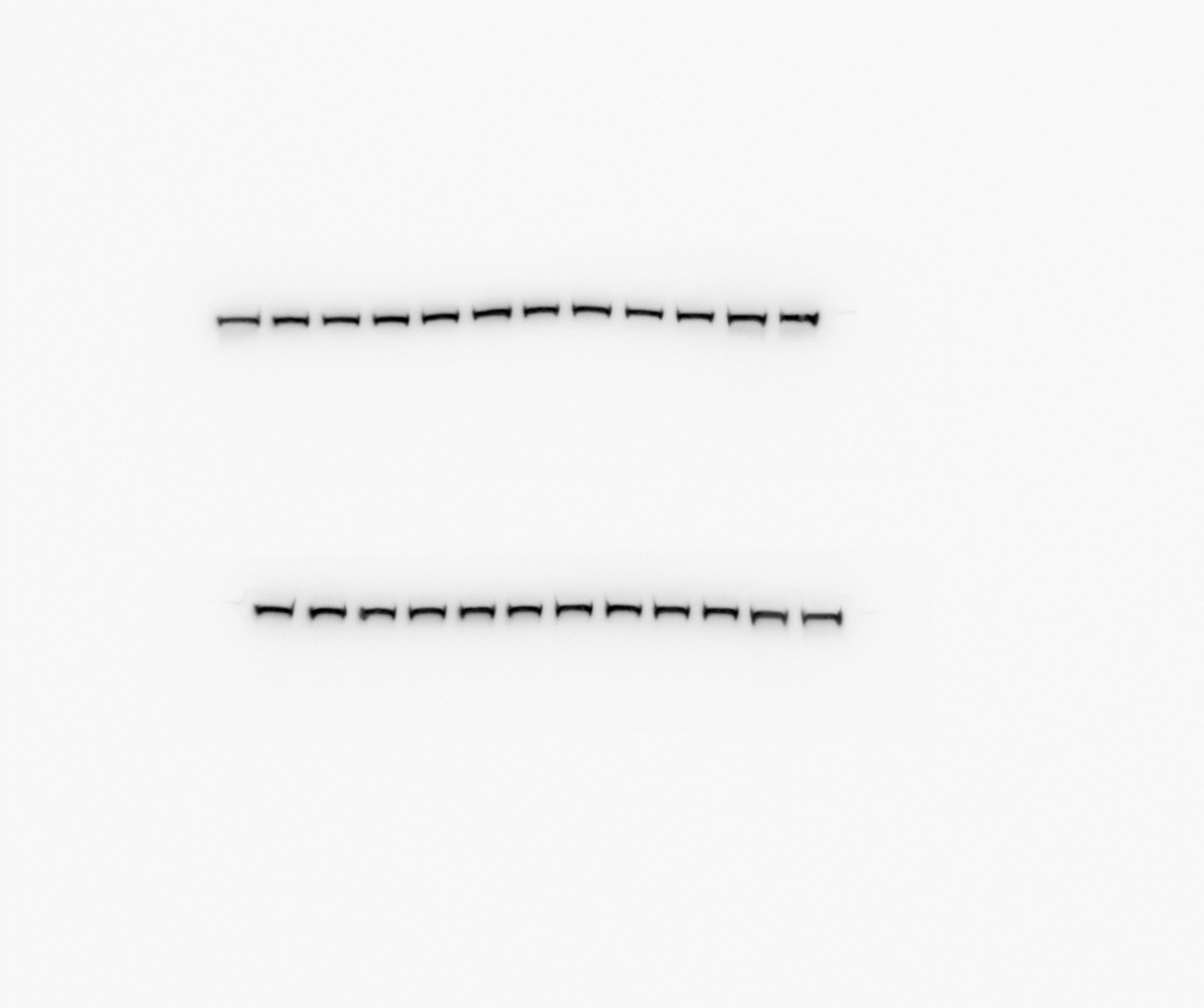

Supplement: Figure 1—source data 2. [file elife-98631-fig1-data2.zip › 1D Vinculin.tif]

Images corresponding to **Figure 1 – figure supplement 1**:

B

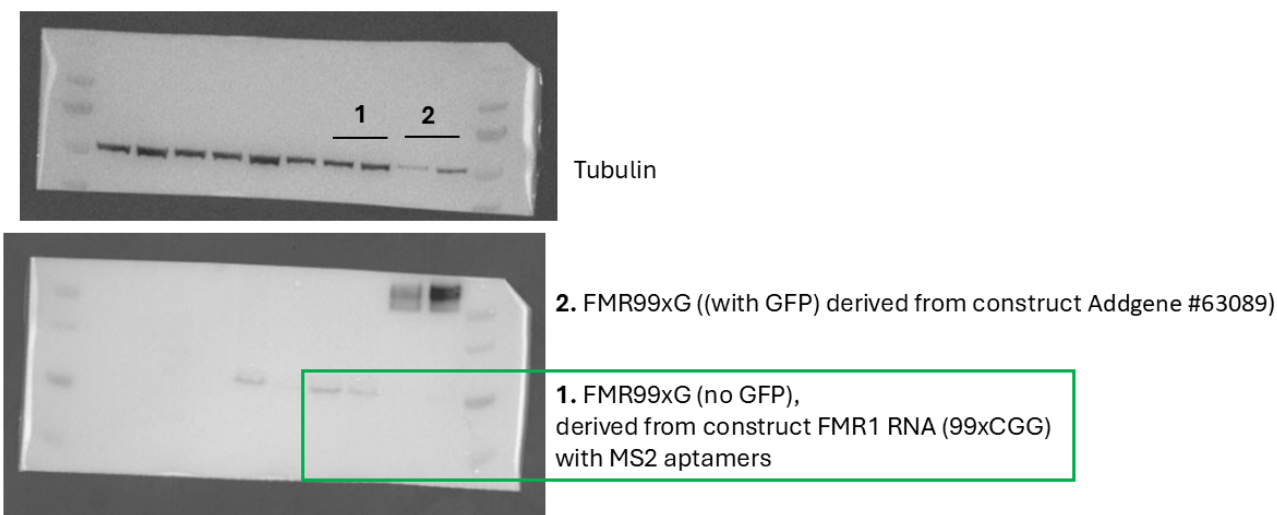

D

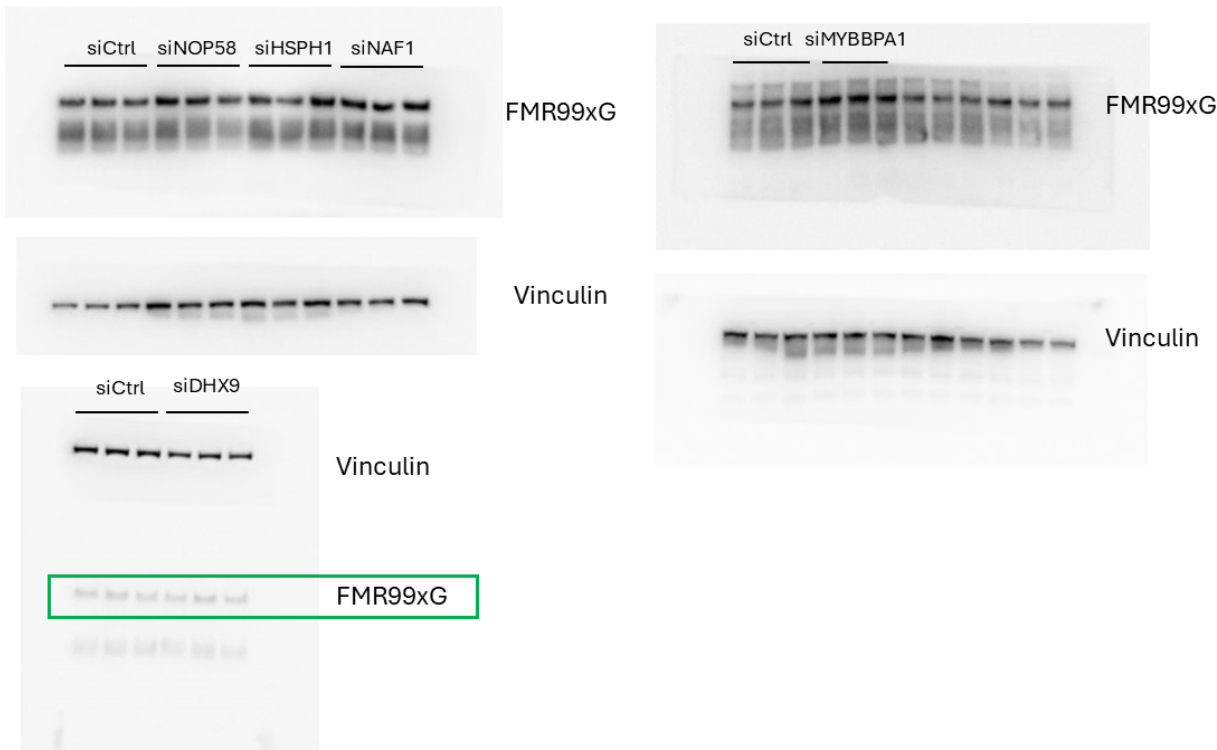

G

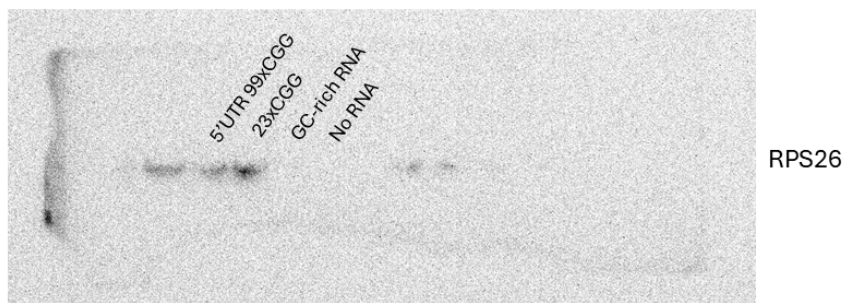

H

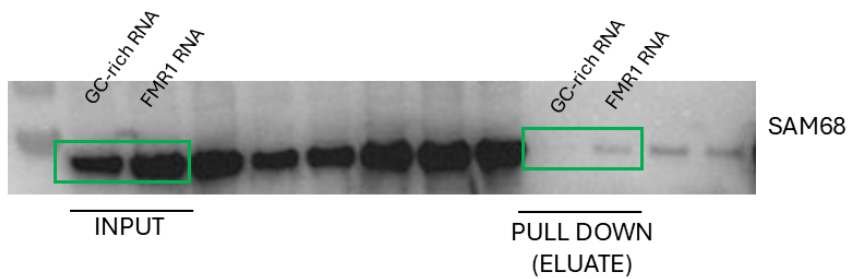

Supplement: Figure 1—figure supplement 1—source data 1. [file elife-98631-fig1-figsupp1-data1.zip › Figure 1 - figure supplement 1.pdf]

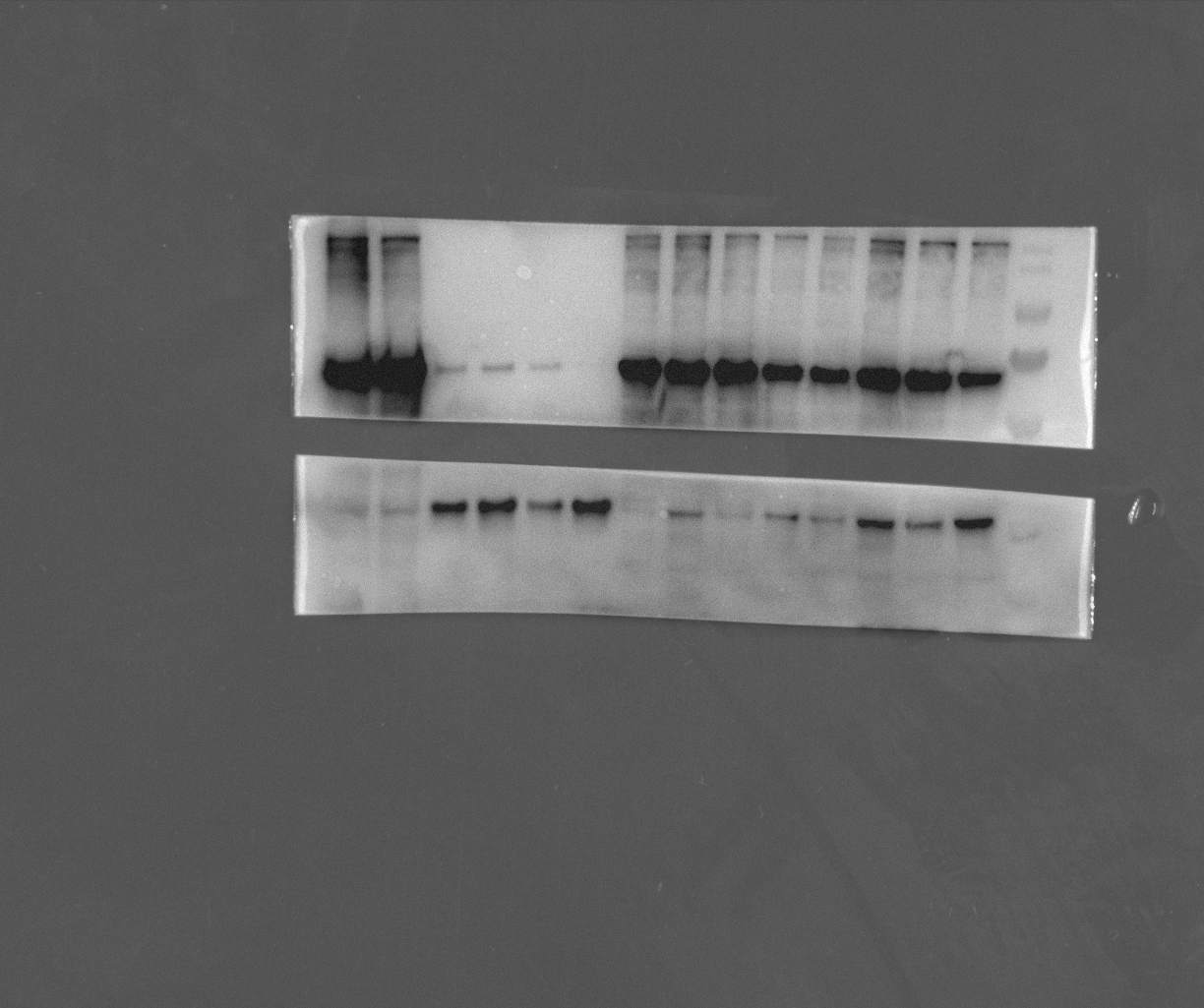

Supplement: Figure 1—figure supplement 1—source data 2. [file elife-98631-fig1-figsupp1-data2.zip › Sfig1G SAM68tif.tif]

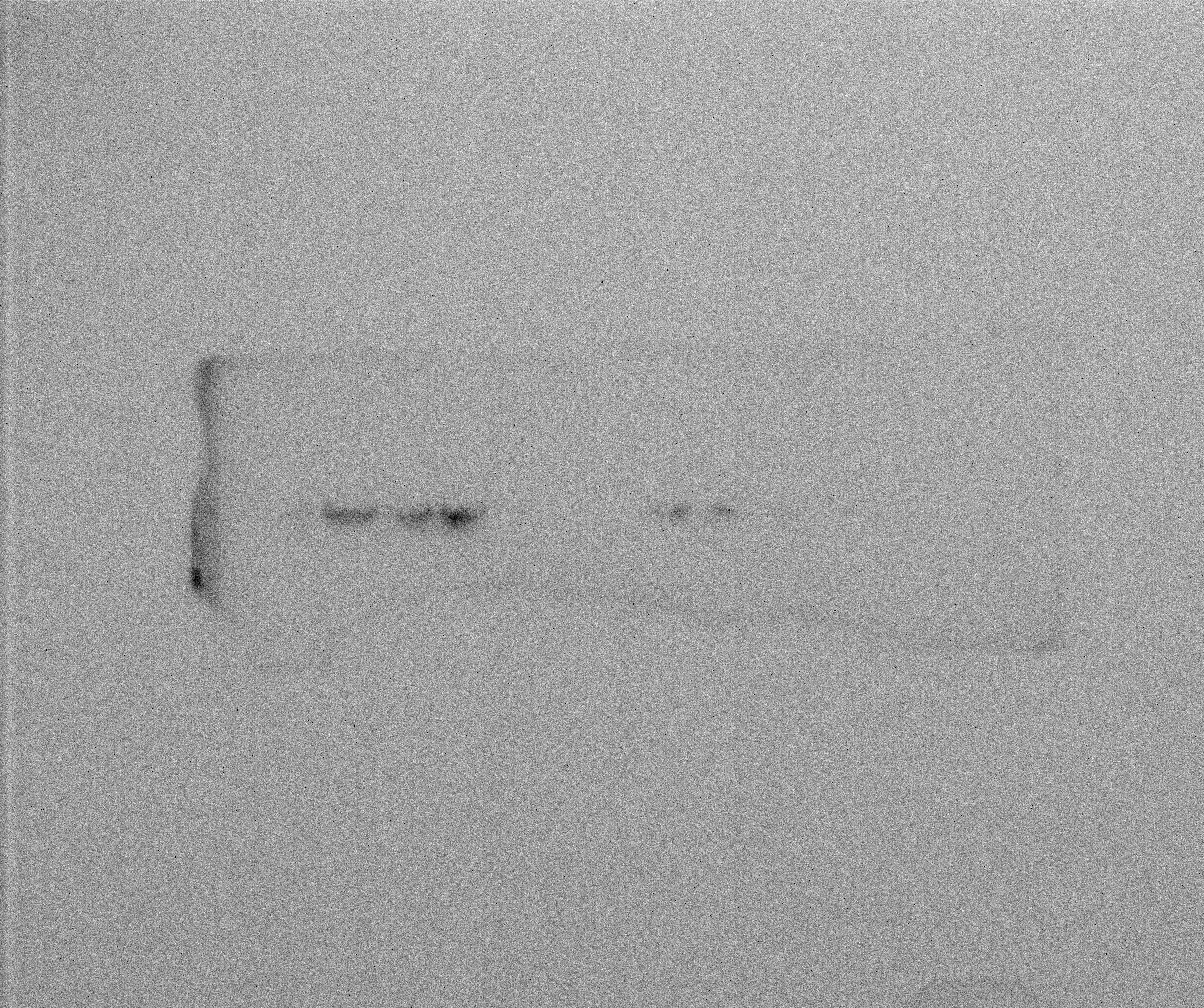

Supplement: Figure 1—figure supplement 1—source data 2. [file elife-98631-fig1-figsupp1-data2.zip › Sfig1F RPS26.tif]

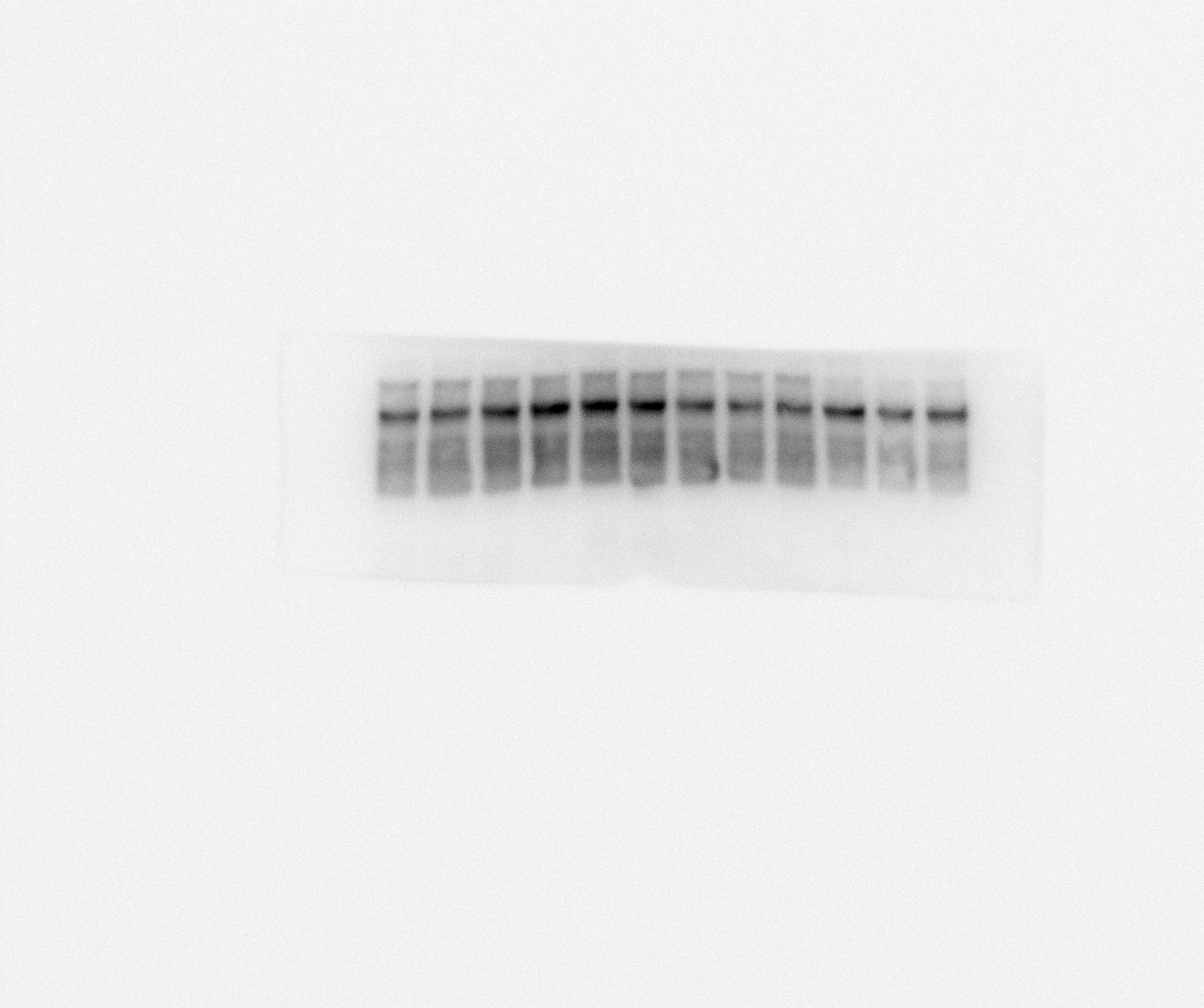

Supplement: Figure 1—figure supplement 1—source data 2. [file elife-98631-fig1-figsupp1-data2.zip › Sfig1DFMR99xG post siMYBBPA1.tif]

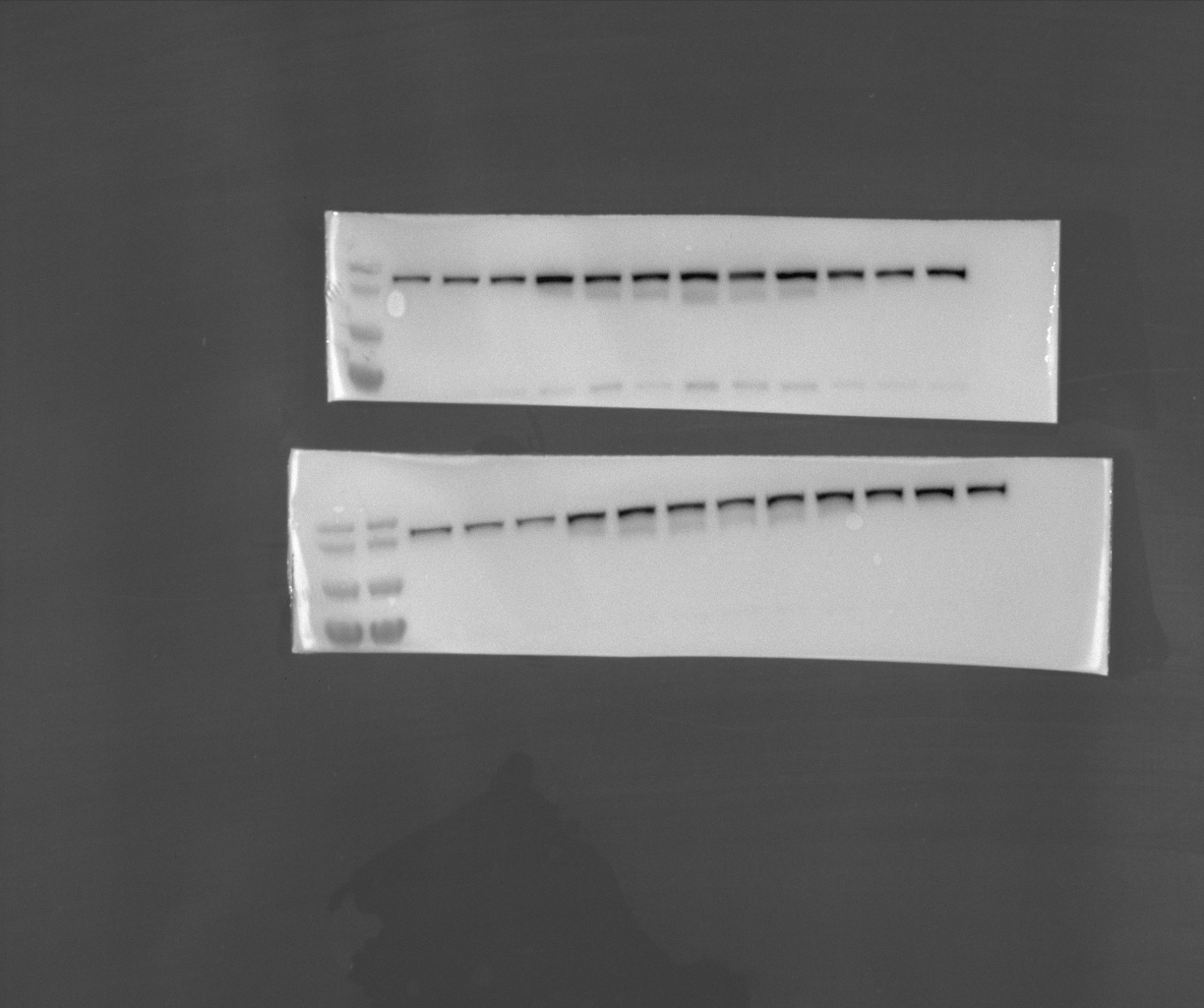

Supplement: Figure 1—figure supplement 1—source data 2. [file elife-98631-fig1-figsupp1-data2.zip › Sfig1D Vinculin to NOP58, HSPH1, NAF1.tif]

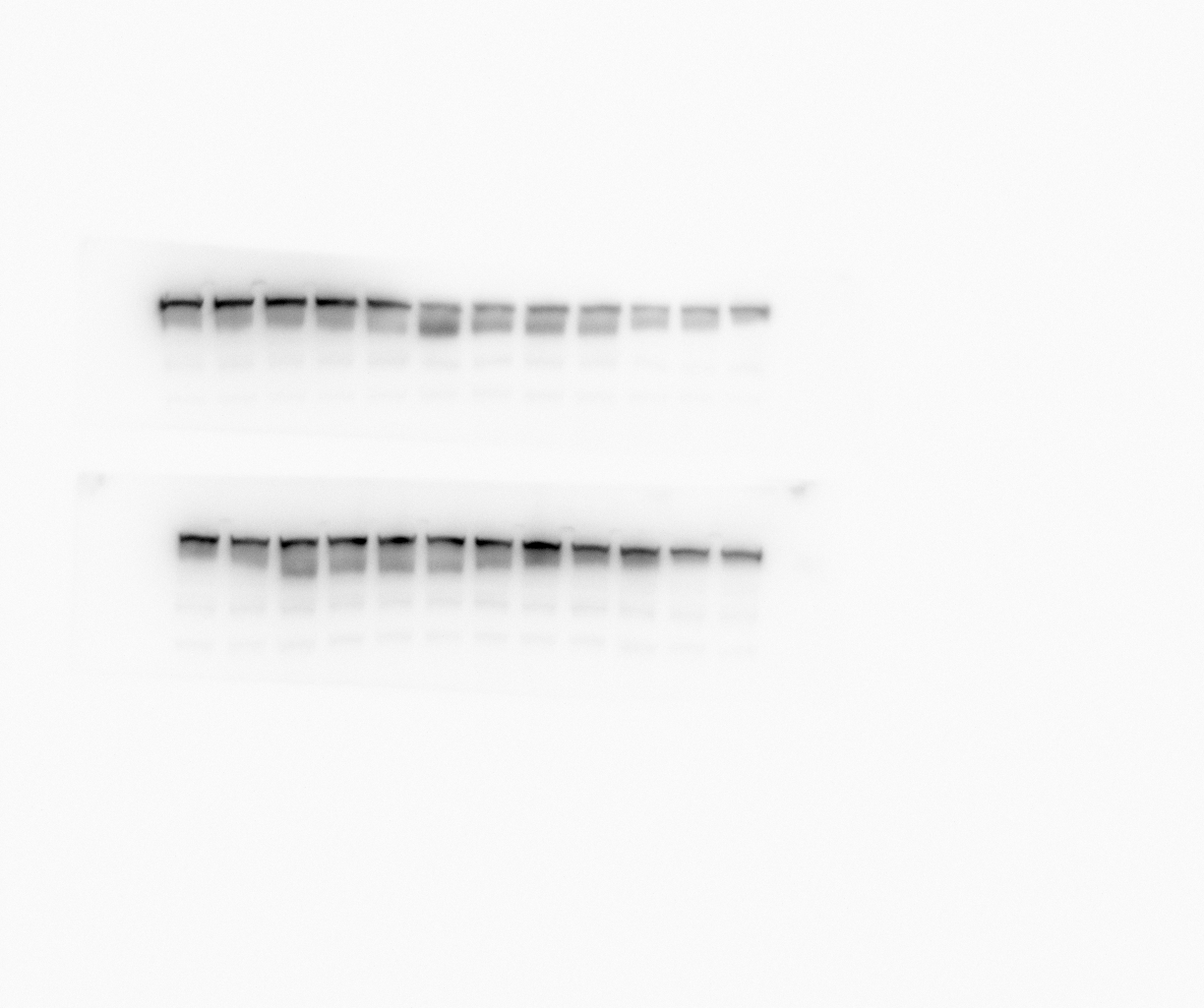

Supplement: Figure 1—figure supplement 1—source data 2. [file elife-98631-fig1-figsupp1-data2.zip › Sfig1D Vinculin to MYBBPA1.tif]

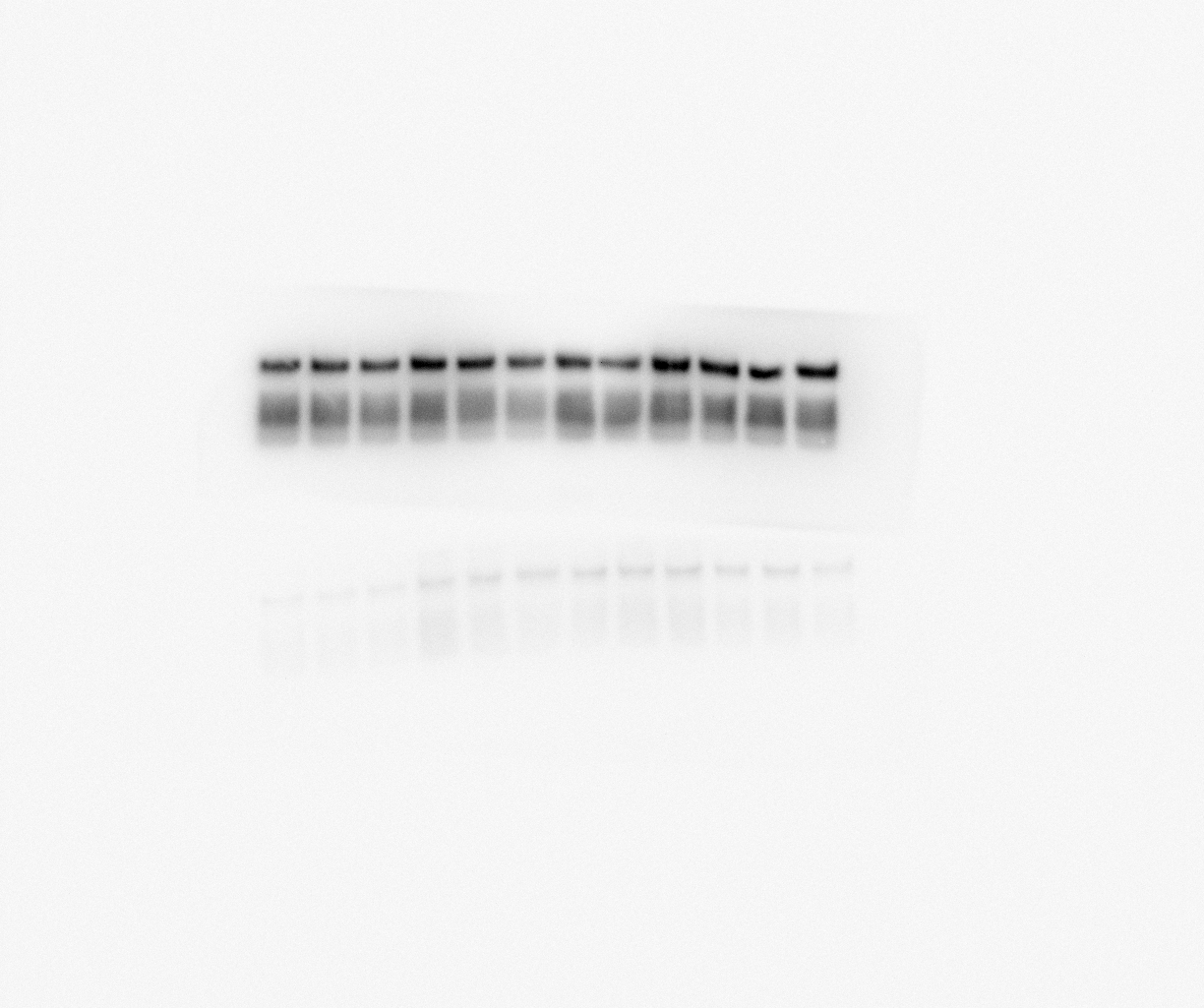

Supplement: Figure 1—figure supplement 1—source data 2. [file elife-98631-fig1-figsupp1-data2.zip › Sfig1D FMR99xG post siNOP siHSPH siNAF.tif]

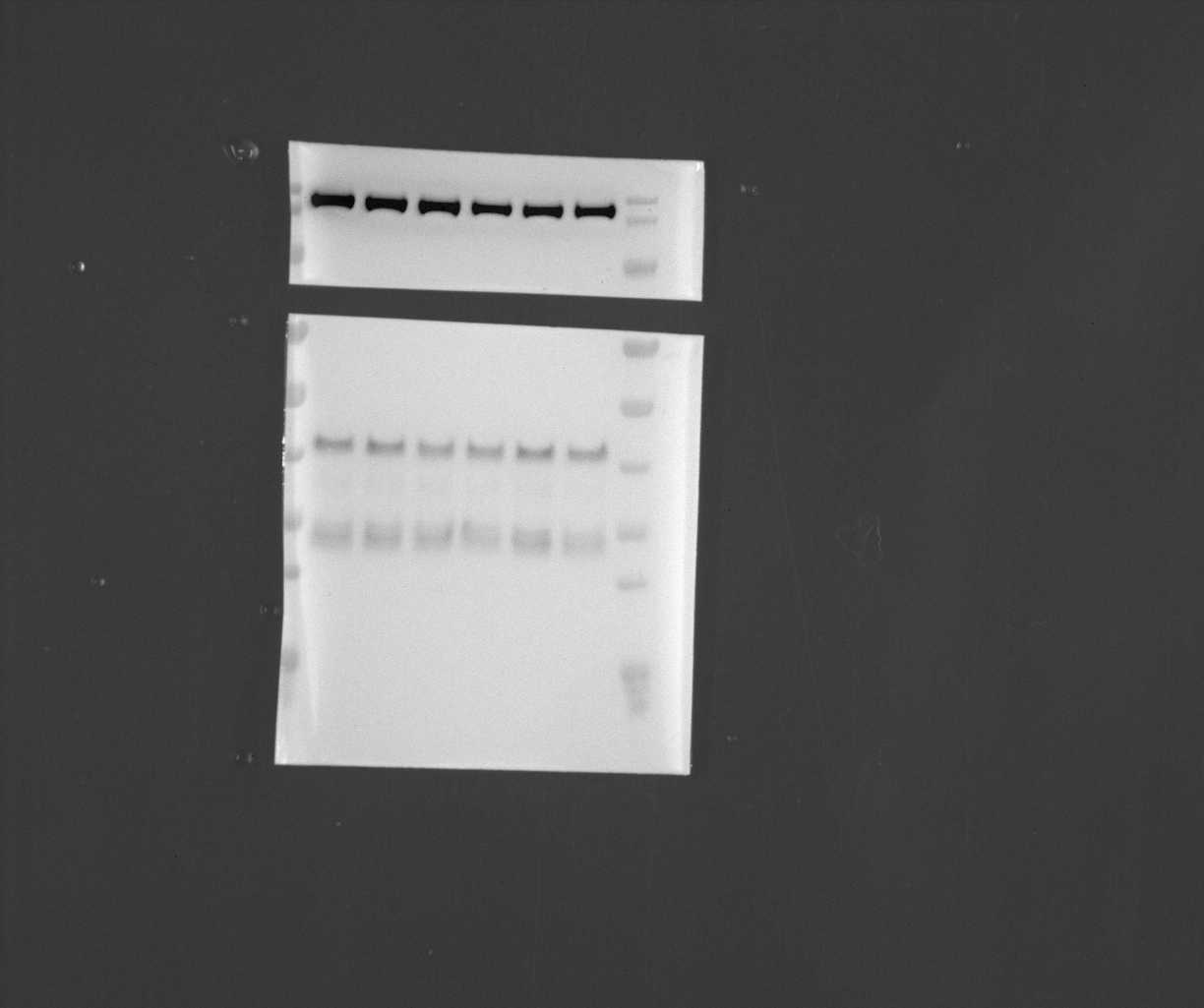

Supplement: Figure 1—figure supplement 1—source data 2. [file elife-98631-fig1-figsupp1-data2.zip › Sfig1D FMR99xG post siDHX9 + Vinculin.tif]

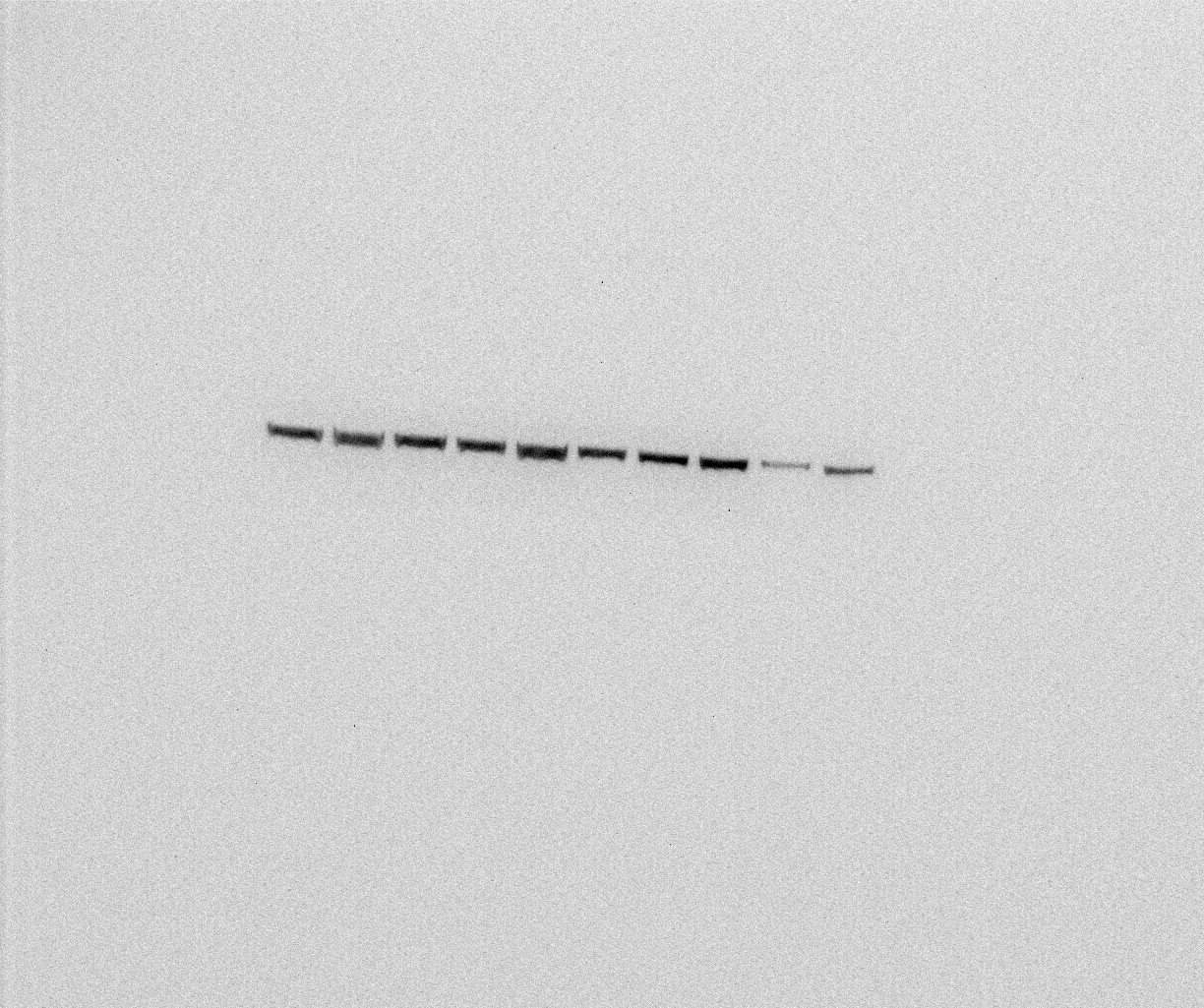

Supplement: Figure 1—figure supplement 1—source data 2. [file elife-98631-fig1-figsupp1-data2.zip › Sfig1B Tubulin.tif]

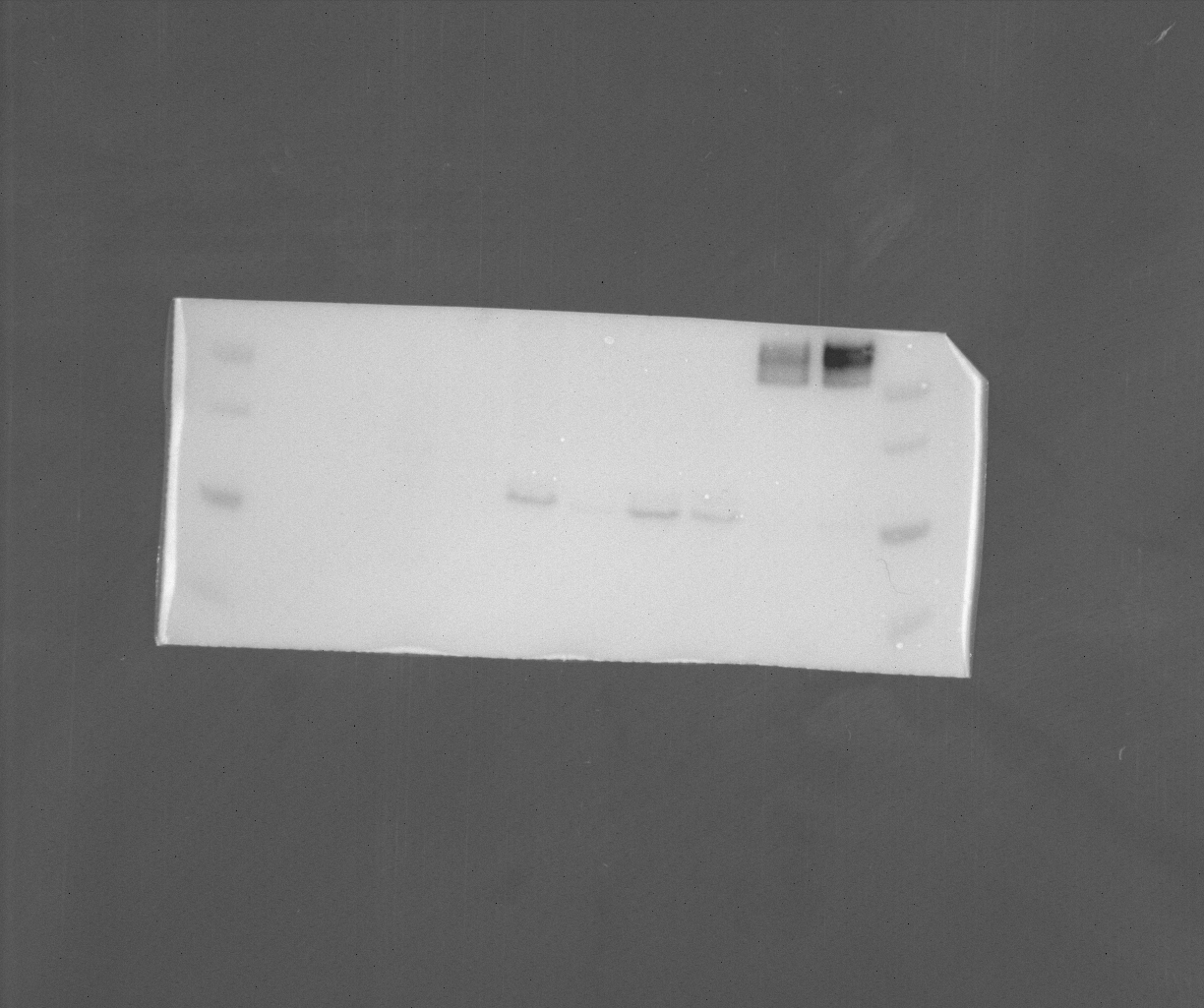

Supplement: Figure 1—figure supplement 1—source data 2. [file elife-98631-fig1-figsupp1-data2.zip › Sfig1B FMRpolyG_GFP.tif]

Images corresponding to **Figure 2**:

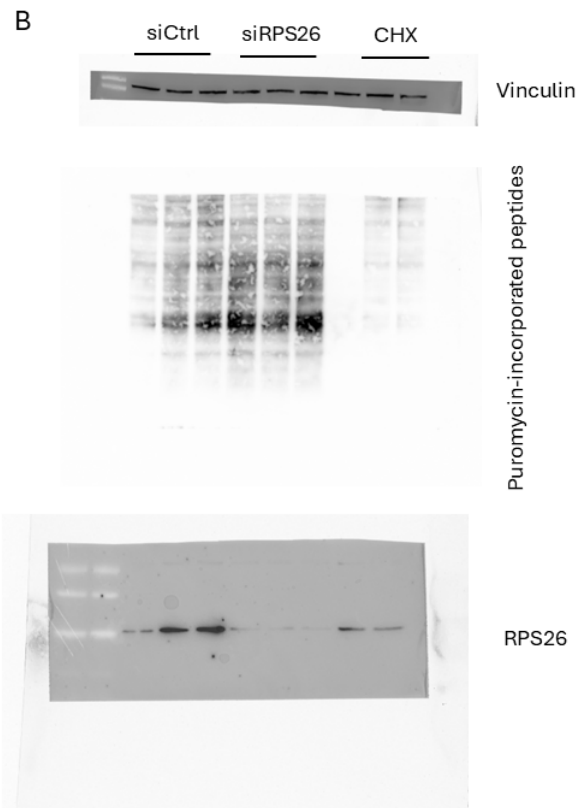

Supplement: Figure 2—source data 1. [file elife-98631-fig2-data1.zip › Figure 2 - source data 1.pdf]

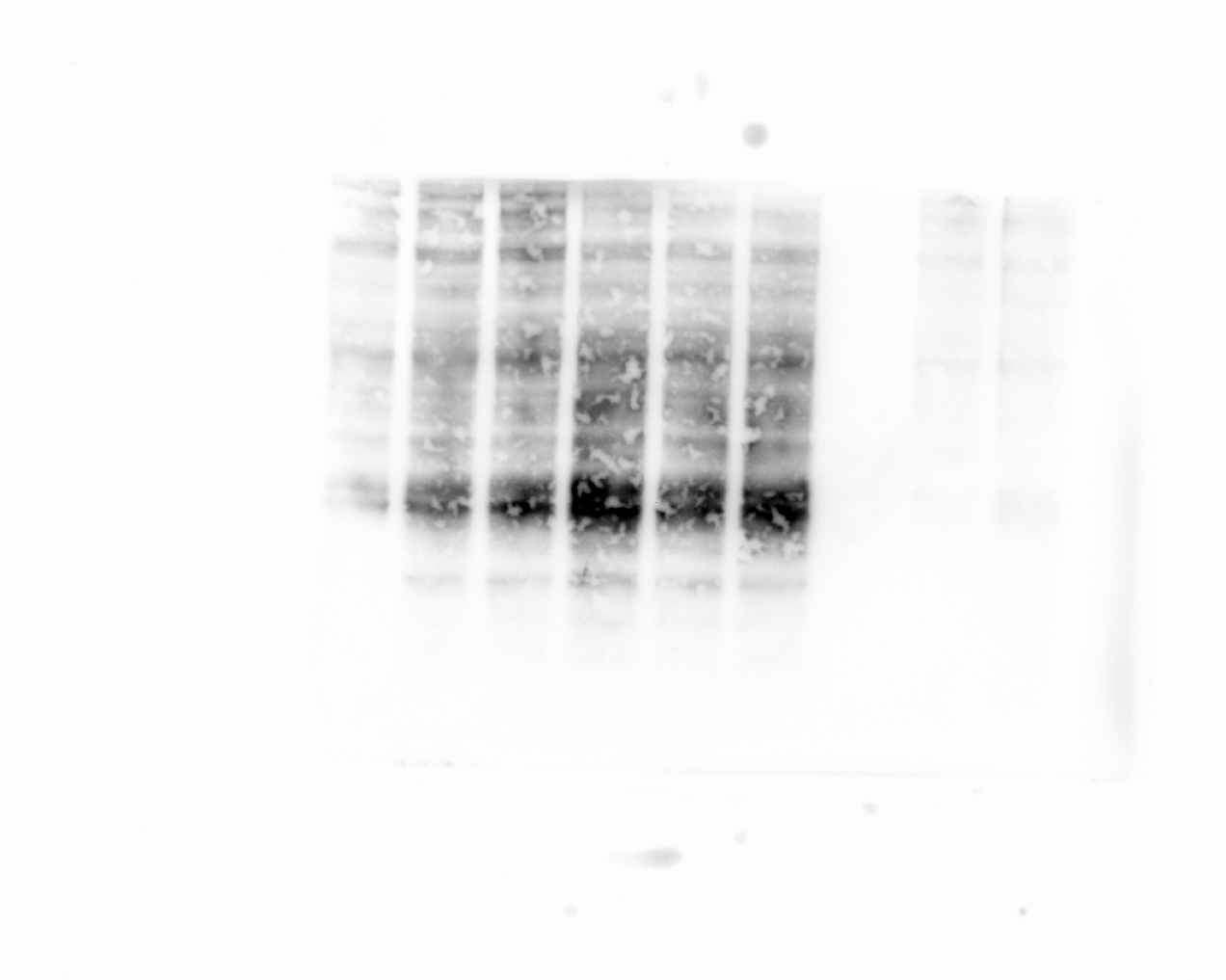

Supplement: Figure 2—source data 2. [file elife-98631-fig2-data2.zip › 2B PURO.tif]

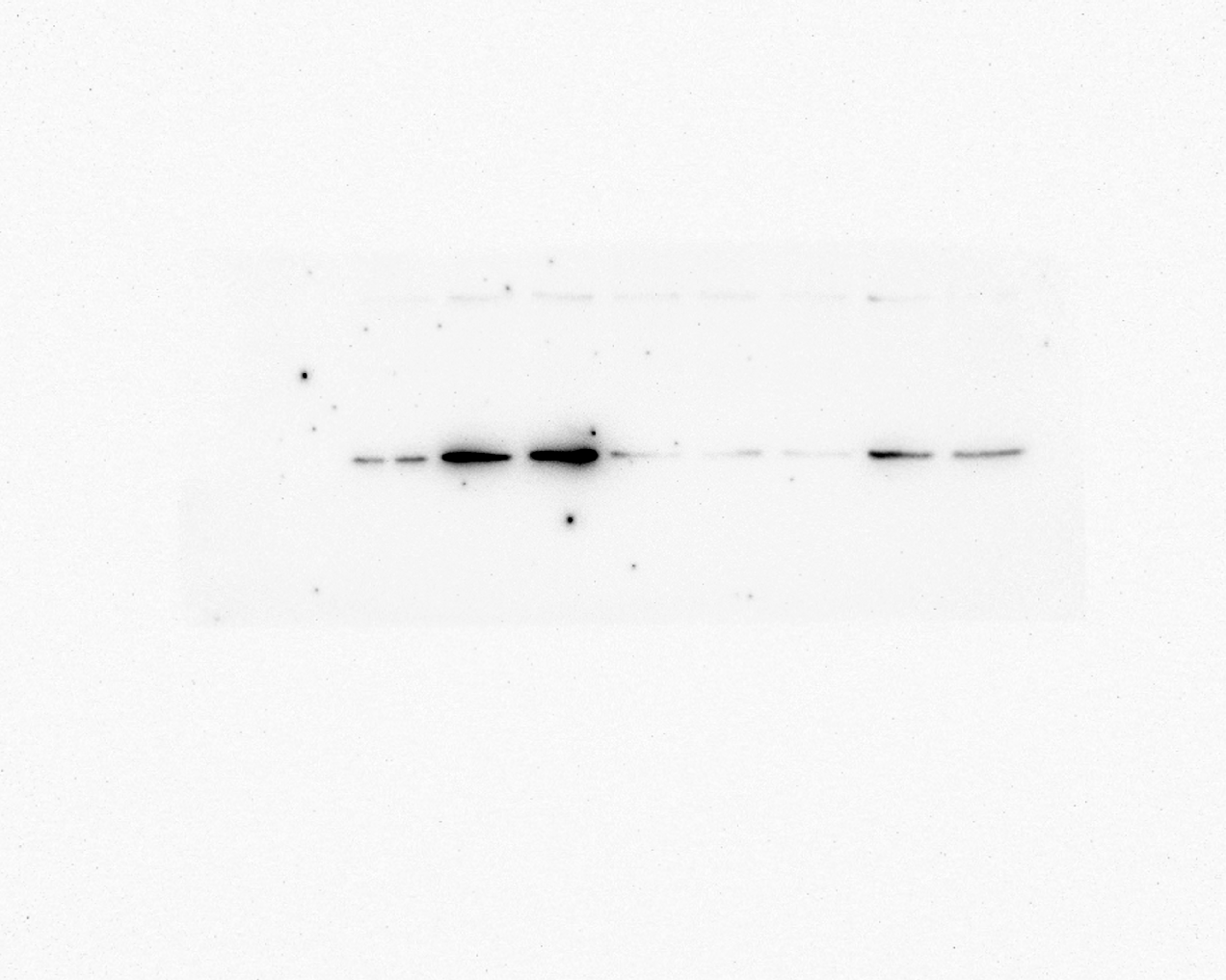

Supplement: Figure 2—source data 2. [file elife-98631-fig2-data2.zip › 2B RPS26.tif]

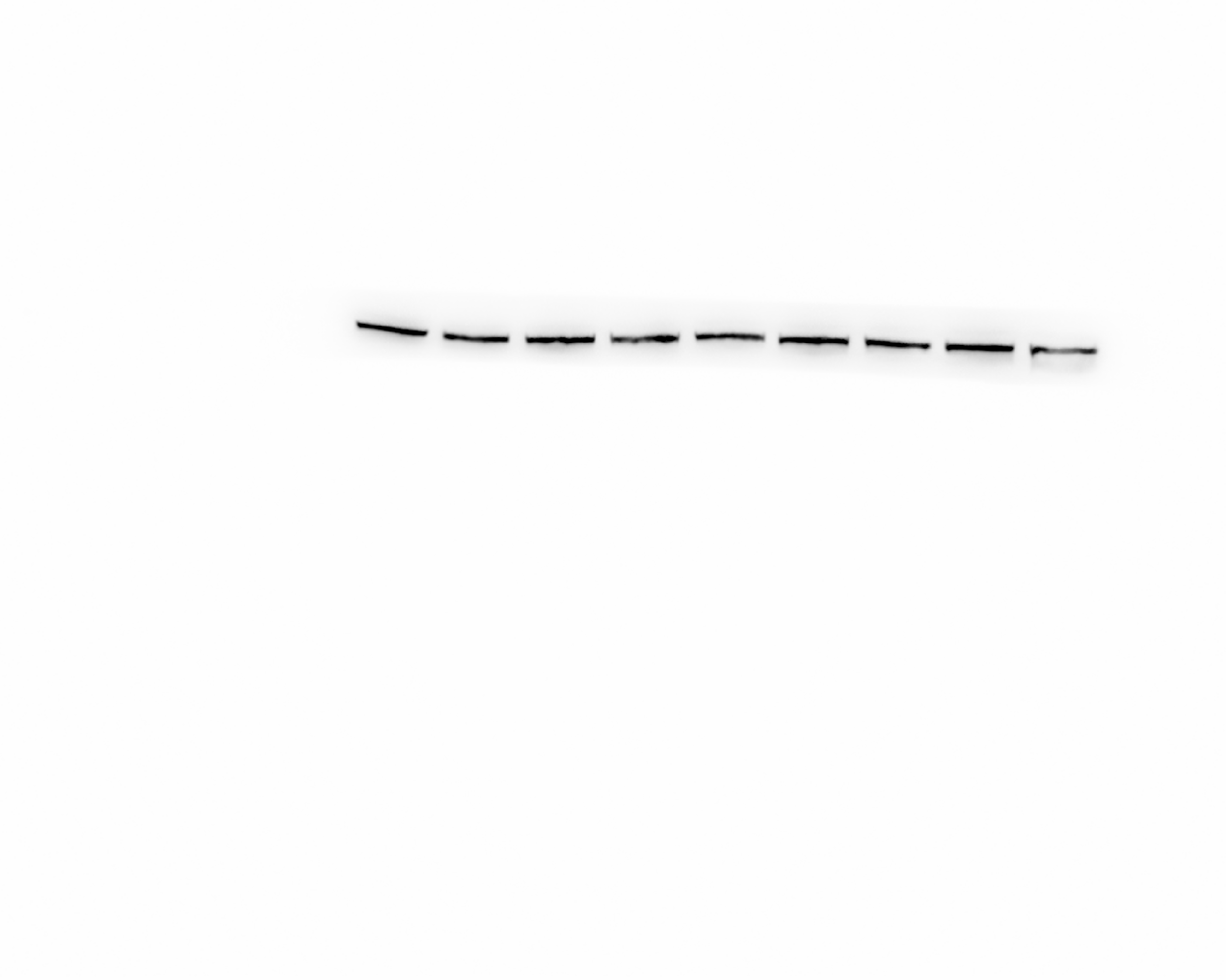

Supplement: Figure 2—source data 2. [file elife-98631-fig2-data2.zip › 2B Vinculin.tif]

Images corresponding to **Figure 3**:

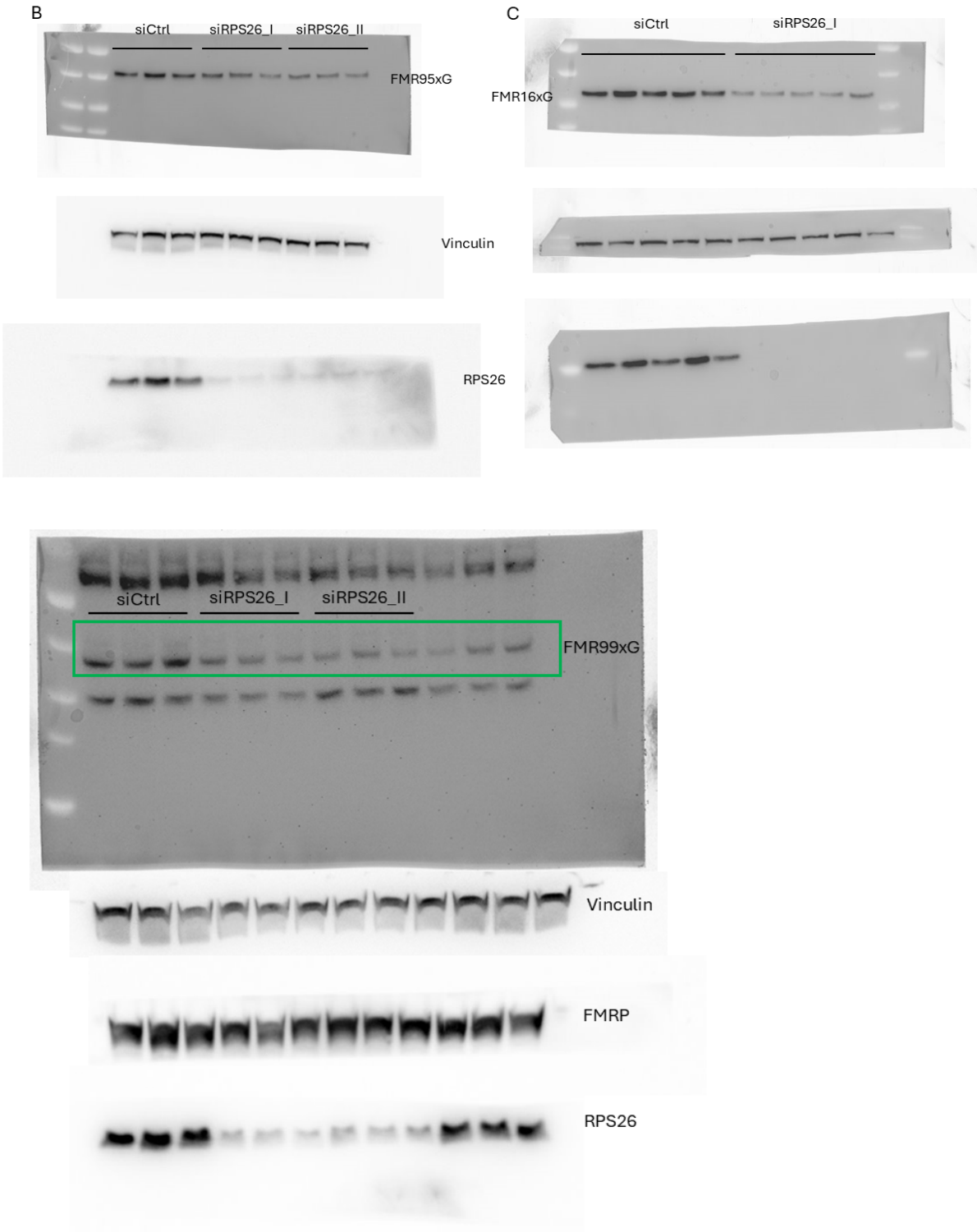

F

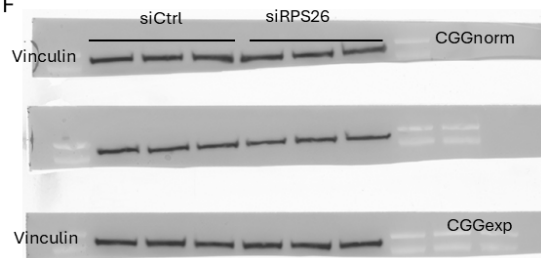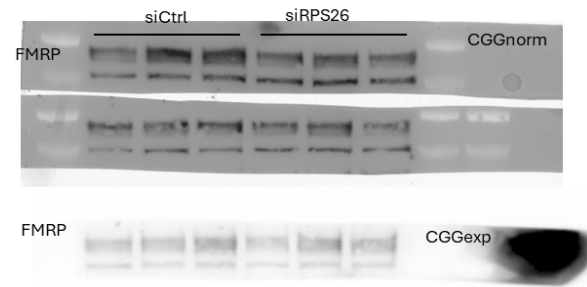

Supplement: Figure 3—source data 1. [file elife-98631-fig3-data1.zip › Figure 3 - source data 1.pdf]

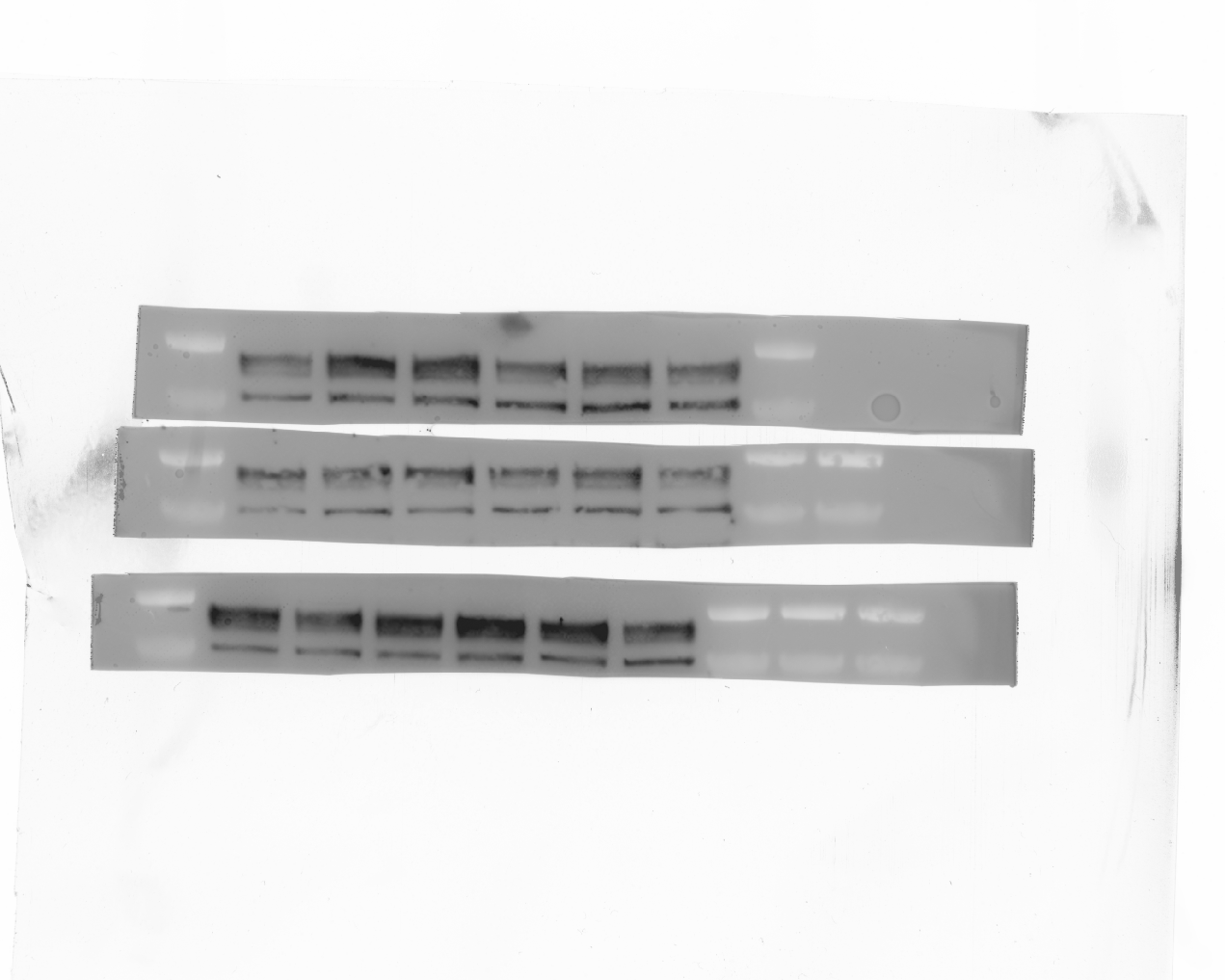

Supplement: Figure 3—source data 2. [file elife-98631-fig3-data2.zip › Figure 3 /3F FMRP CGGnorm.tif]

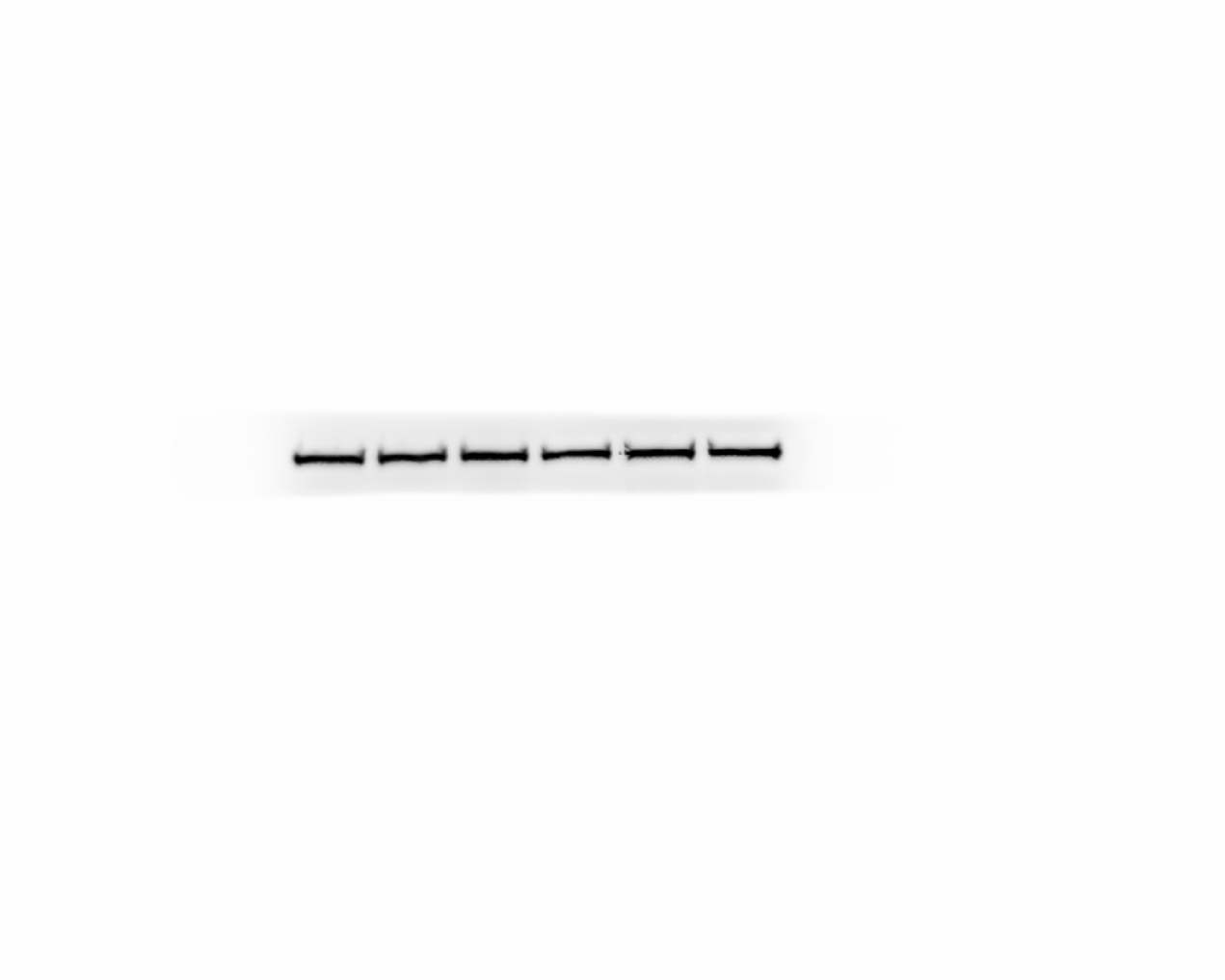

Supplement: Figure 3—source data 2. [file elife-98631-fig3-data2.zip › Figure 3 /3F Vinculin CGGexp:- .tif]

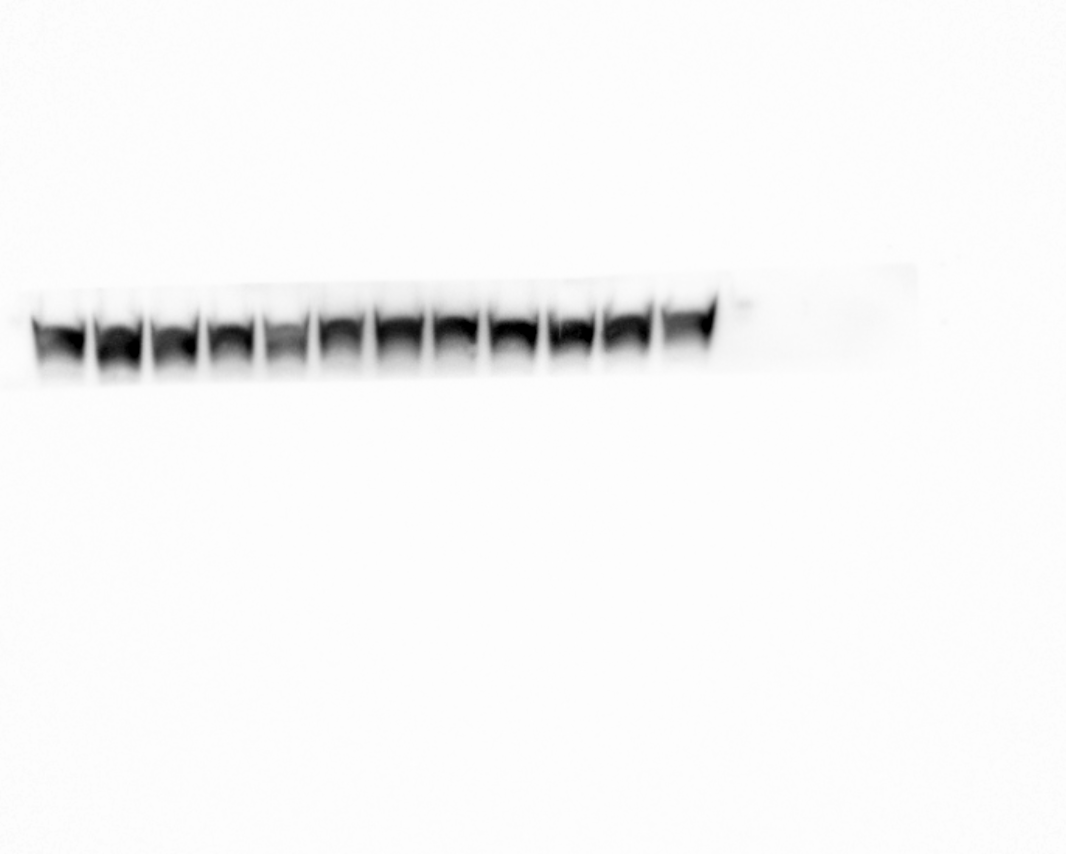

Supplement: Figure 3—source data 2. [file elife-98631-fig3-data2.zip › Figure 3 /3E FMRP.tif]

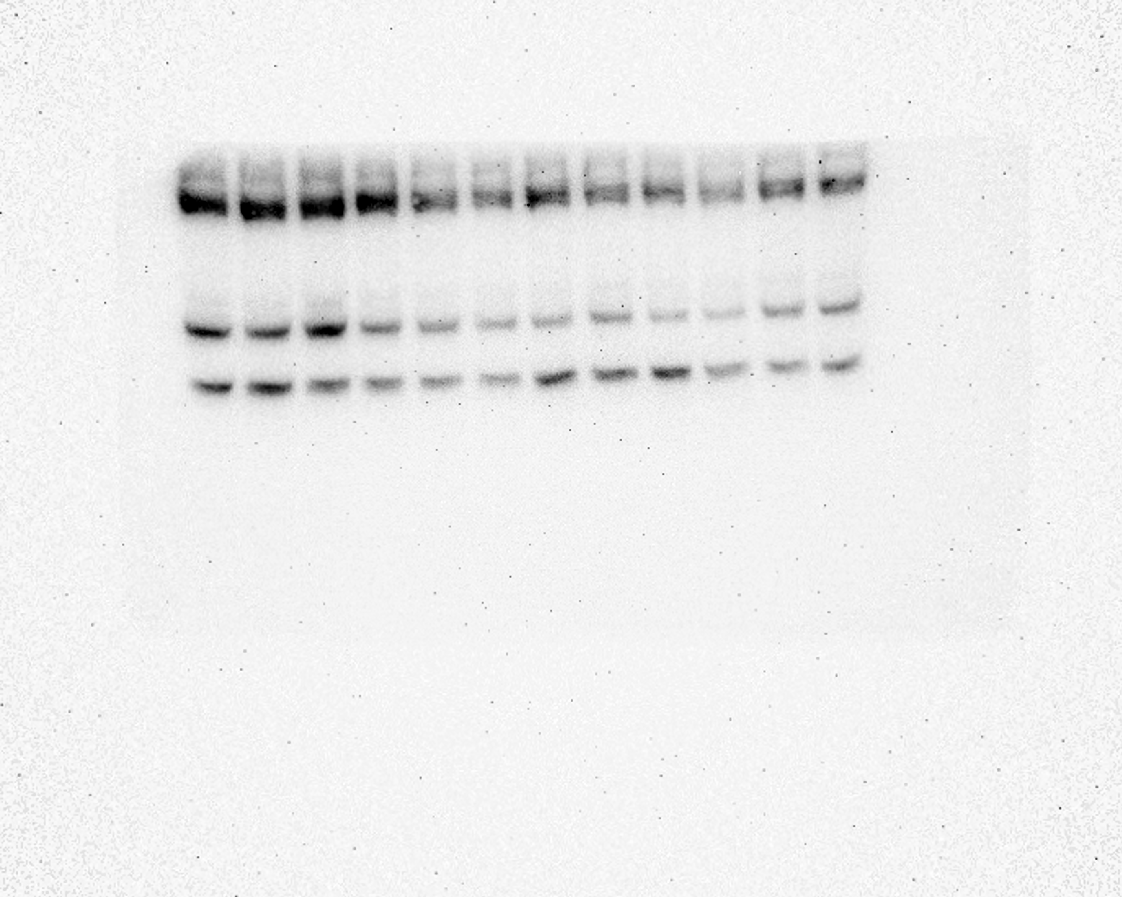

Supplement: Figure 3—source data 2. [file elife-98631-fig3-data2.zip › Figure 3 /3E FMR99xG.tif]

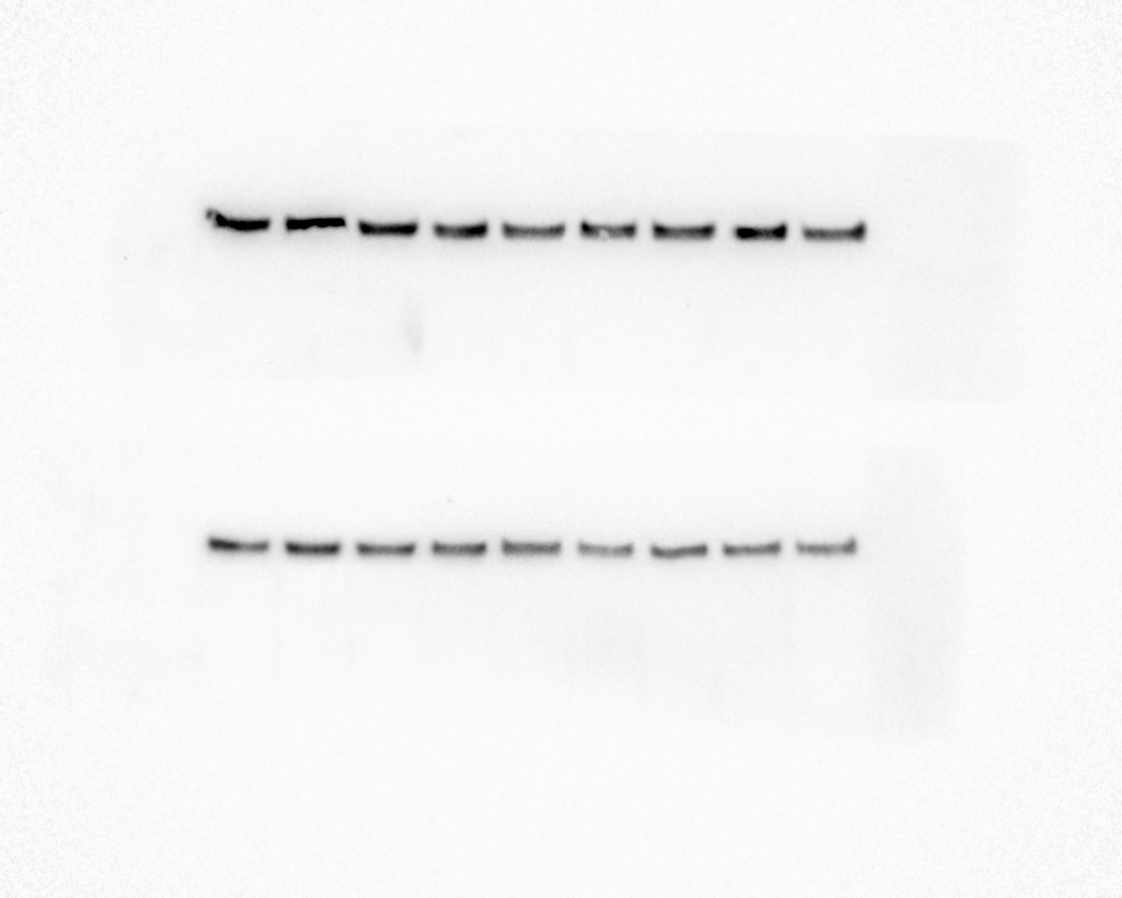

Supplement: Figure 3—source data 2. [file elife-98631-fig3-data2.zip › Figure 3 /3B FMR95xG.tif]

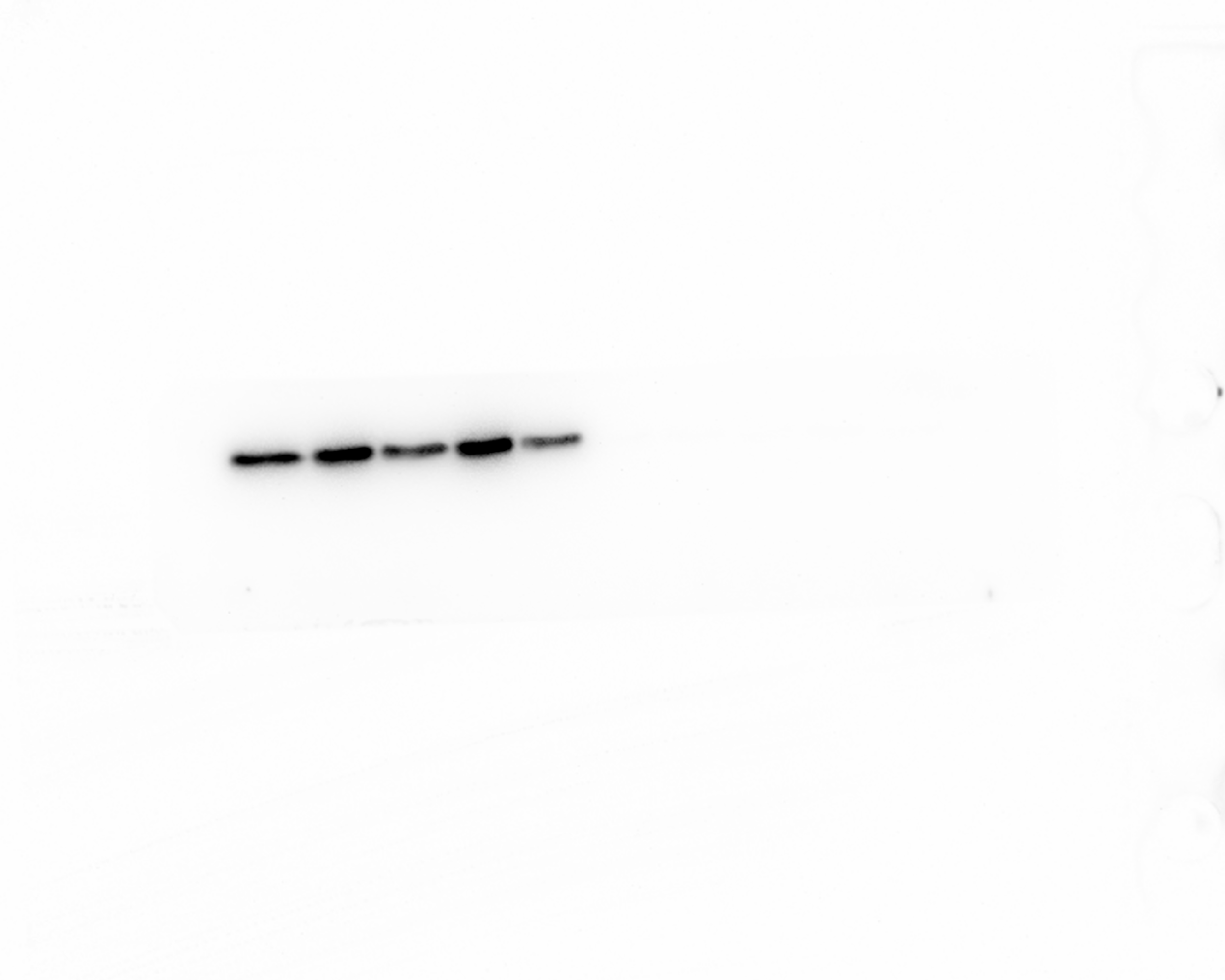

Supplement: Figure 3—source data 2. [file elife-98631-fig3-data2.zip › Figure 3 /3C RPS26.tif]

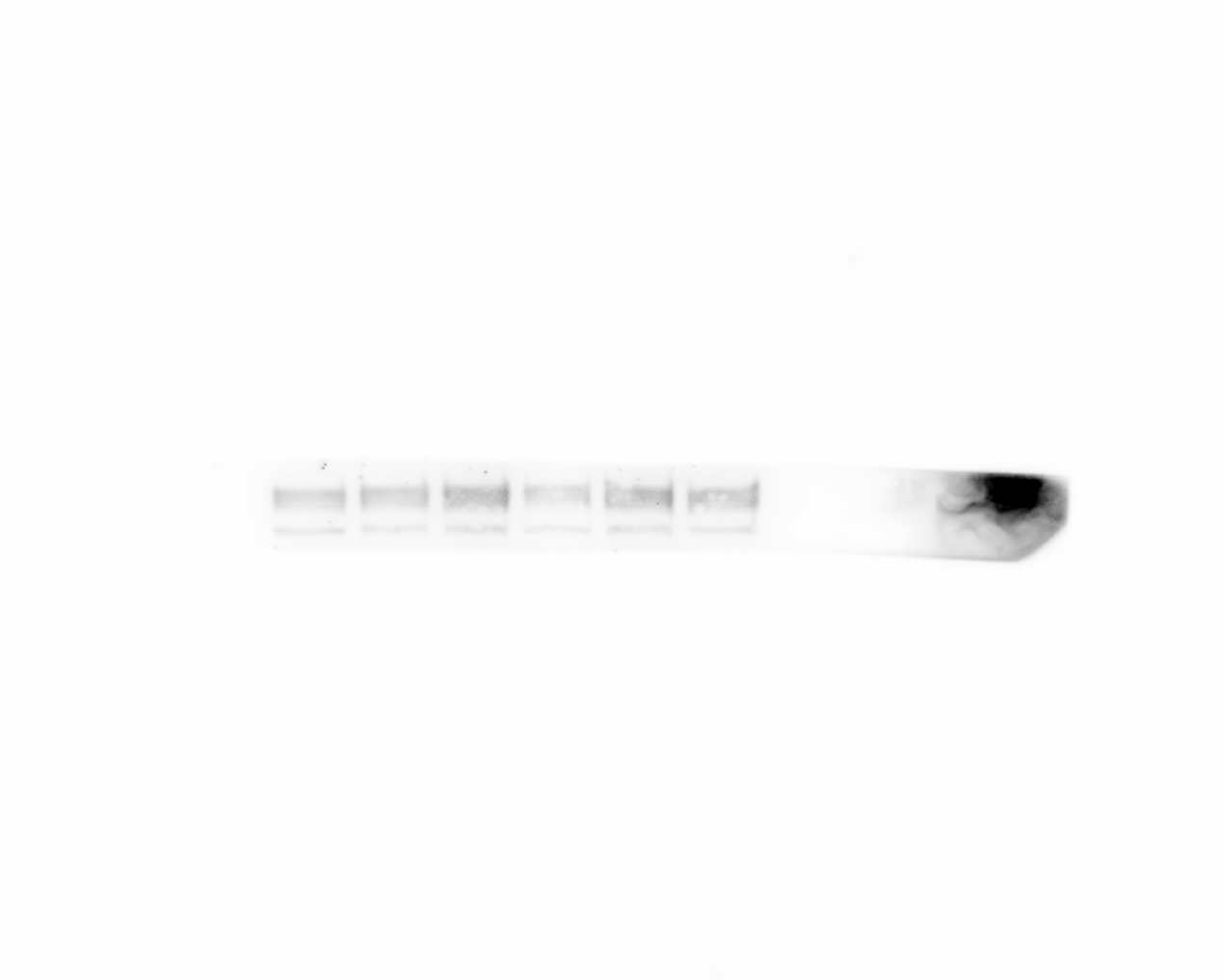

Supplement: Figure 3—source data 2. [file elife-98631-fig3-data2.zip › Figure 3 /3F FMRP CGGexp:-.tif]

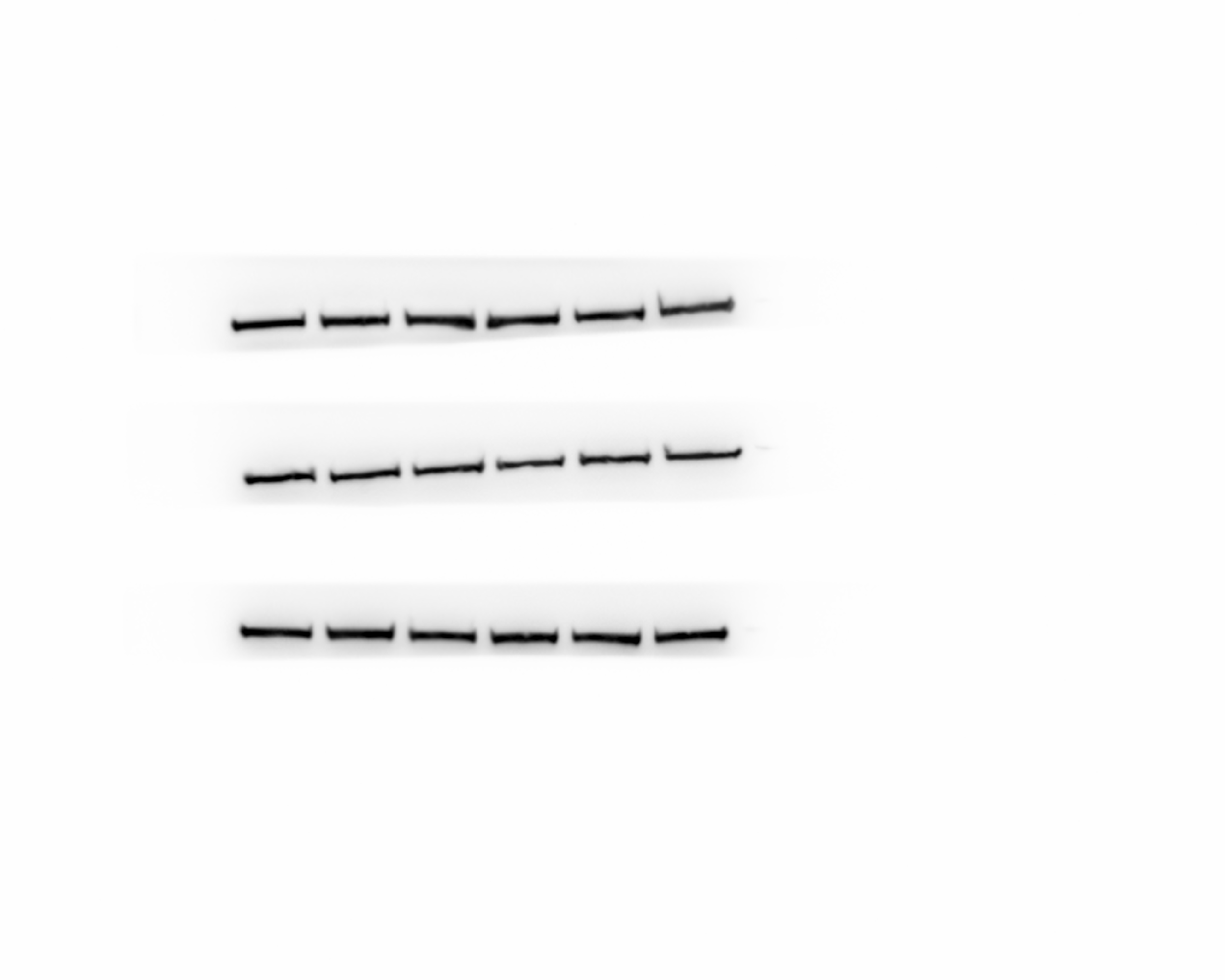

Supplement: Figure 3—source data 2. [file elife-98631-fig3-data2.zip › Figure 3 /3F Vinculin CGGnorm.tif]

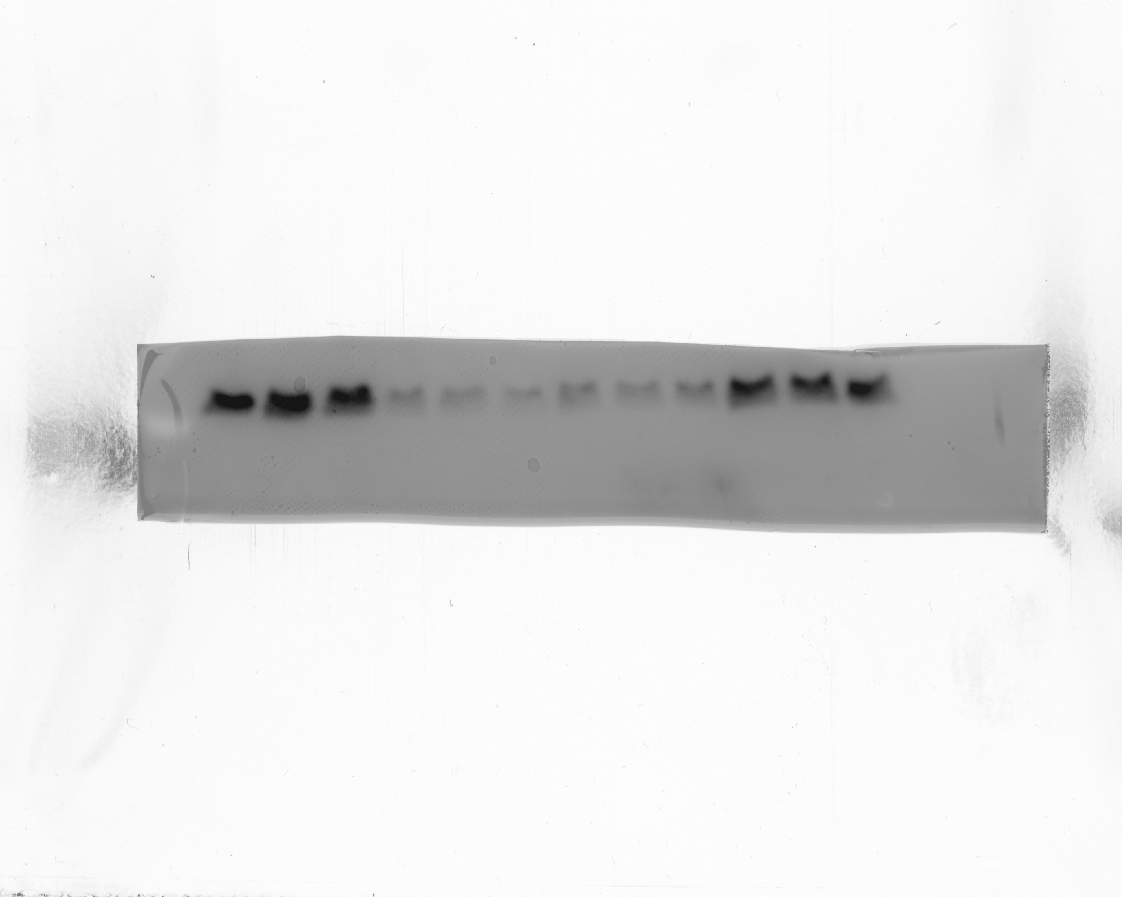

Supplement: Figure 3—source data 2. [file elife-98631-fig3-data2.zip › Figure 3 /3E RPS26.tif]

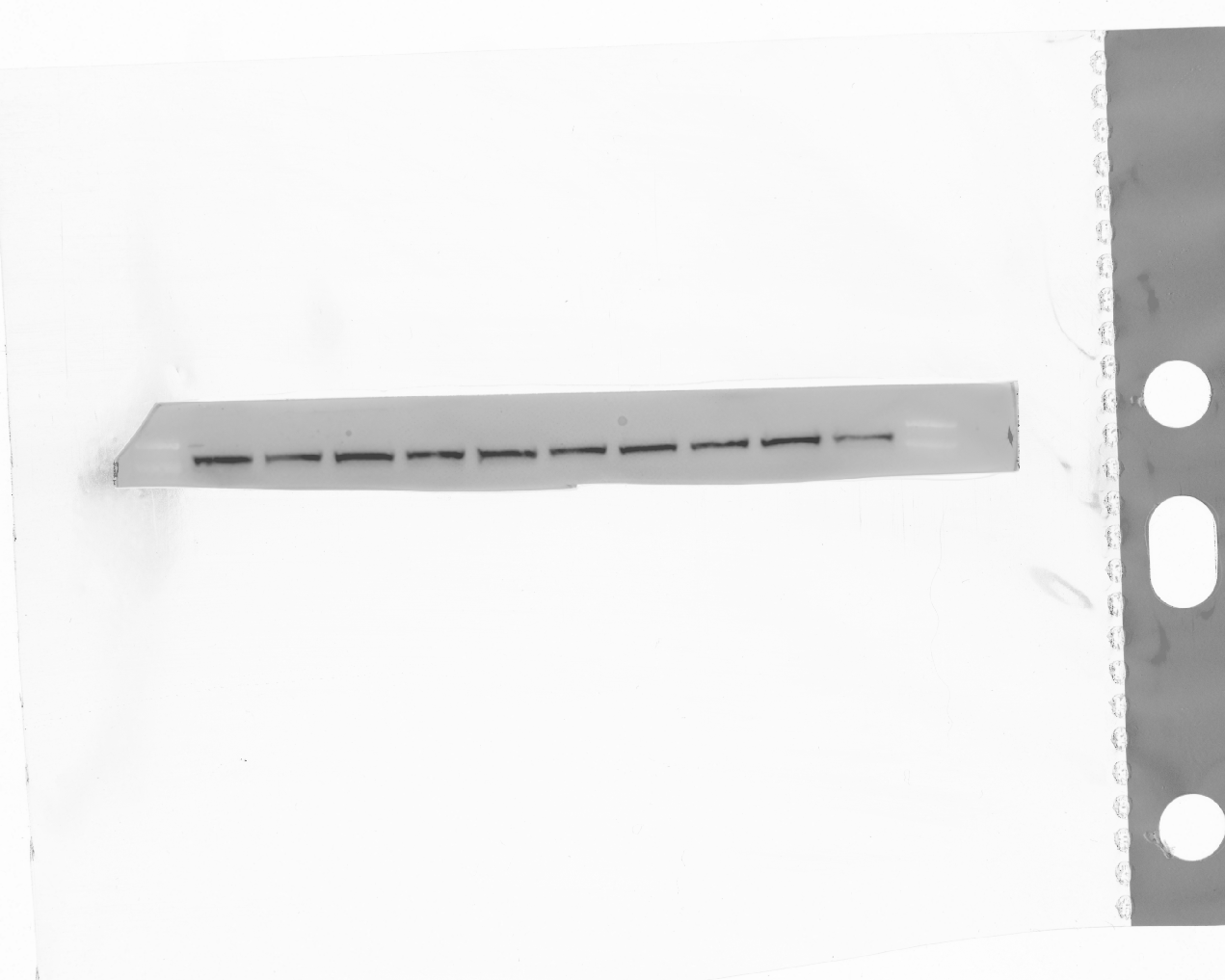

Supplement: Figure 3—source data 2. [file elife-98631-fig3-data2.zip › Figure 3 /3C Vinculin.tif]

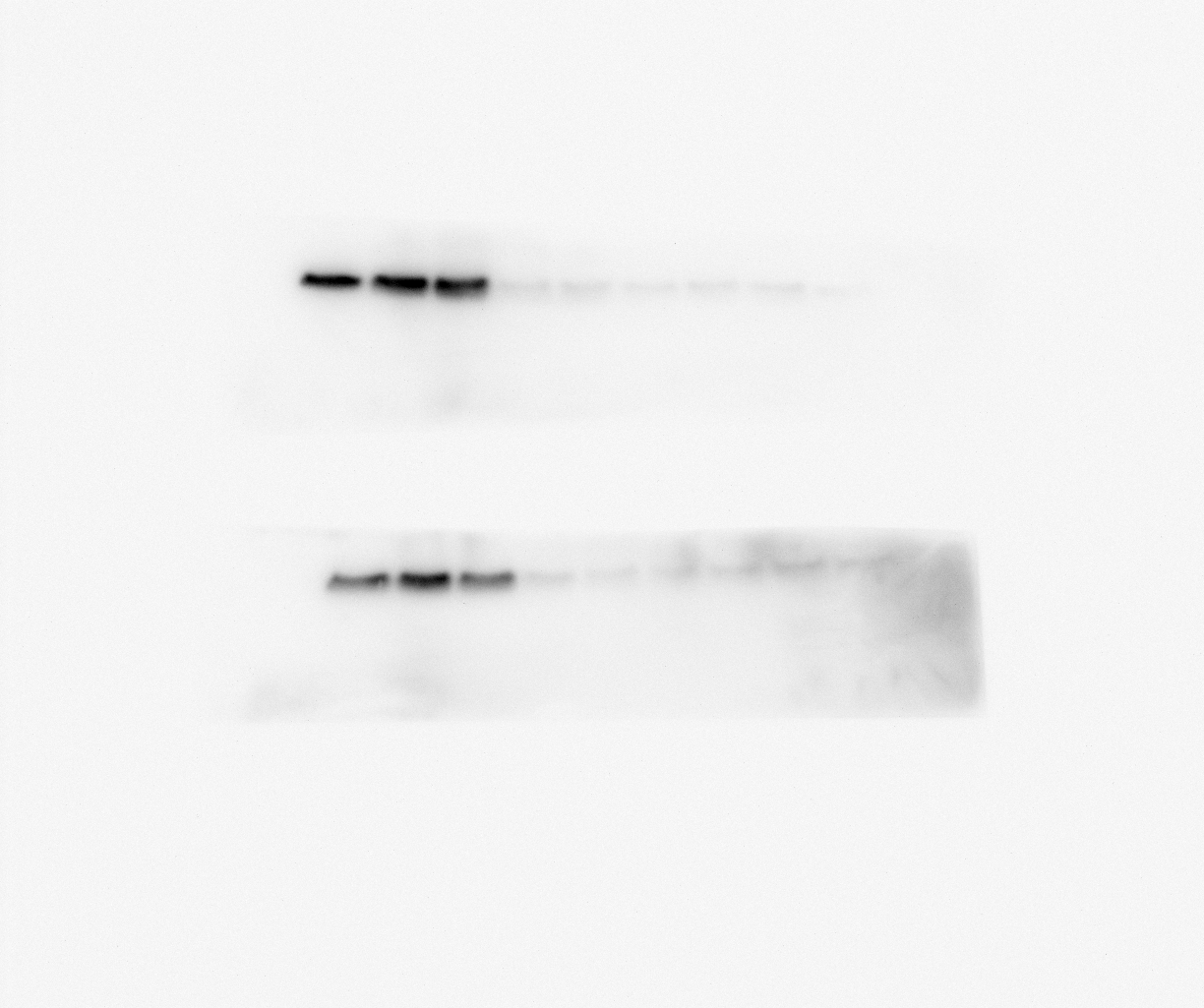

Supplement: Figure 3—source data 2. [file elife-98631-fig3-data2.zip › Figure 3 /3B RPS26.tif]

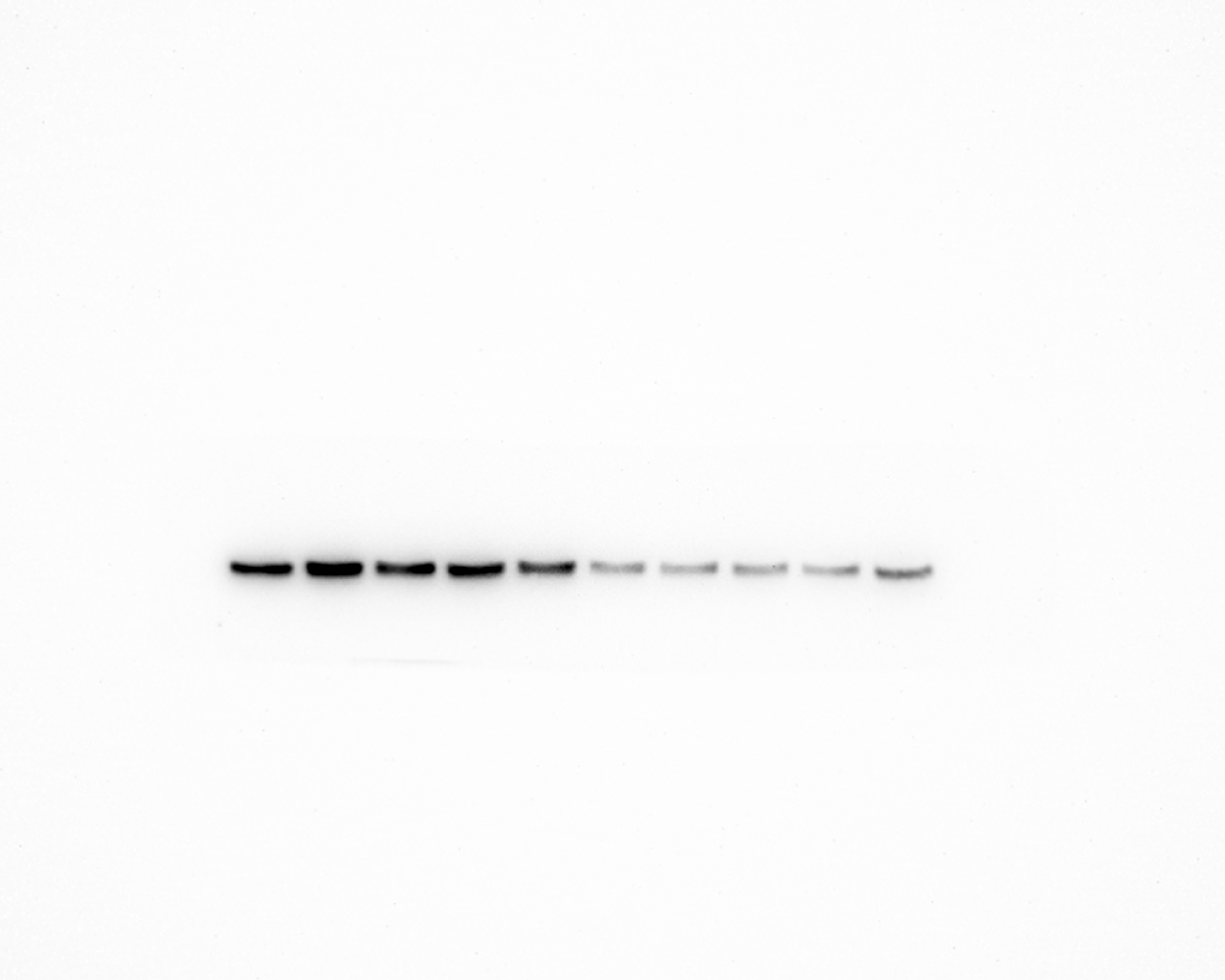

Supplement: Figure 3—source data 2. [file elife-98631-fig3-data2.zip › Figure 3 /3C FMR16xG.tif]

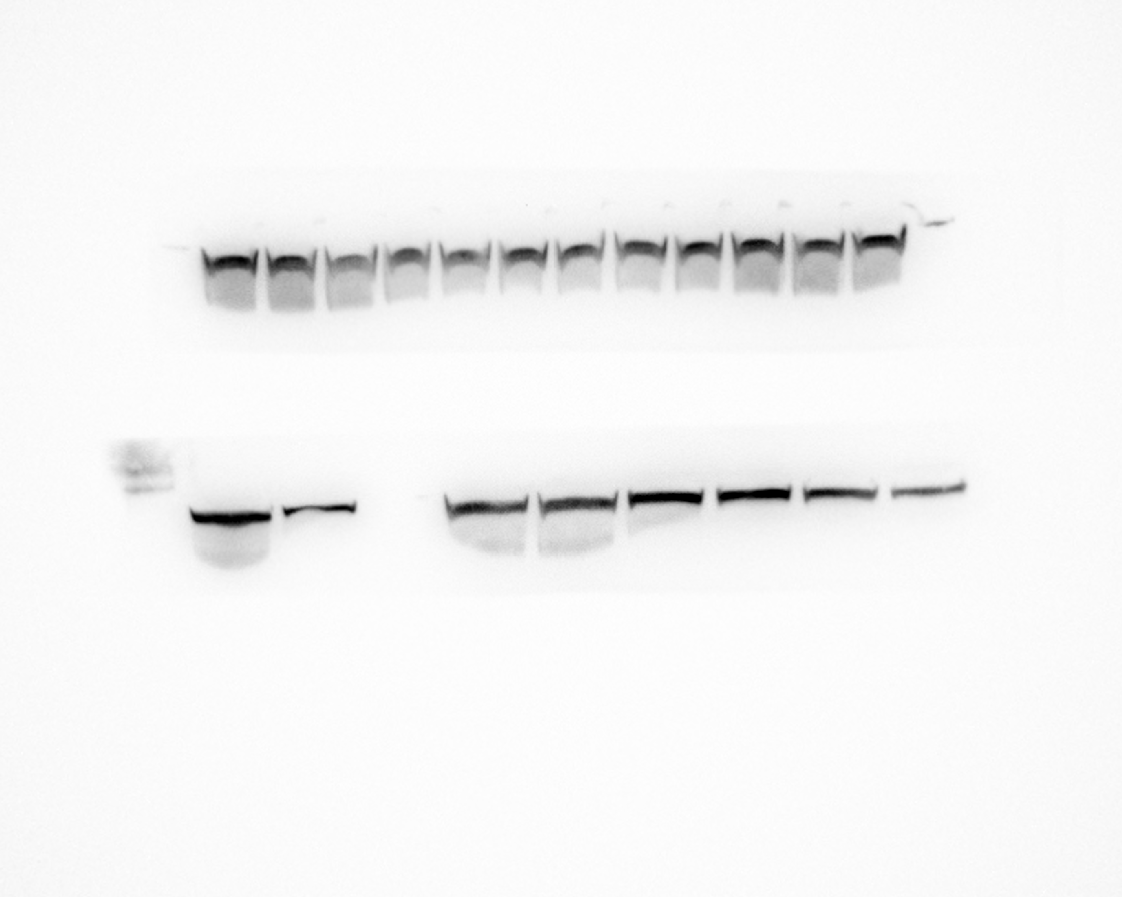

Supplement: Figure 3—source data 2. [file elife-98631-fig3-data2.zip › Figure 3 /3E Vinculin.tif]

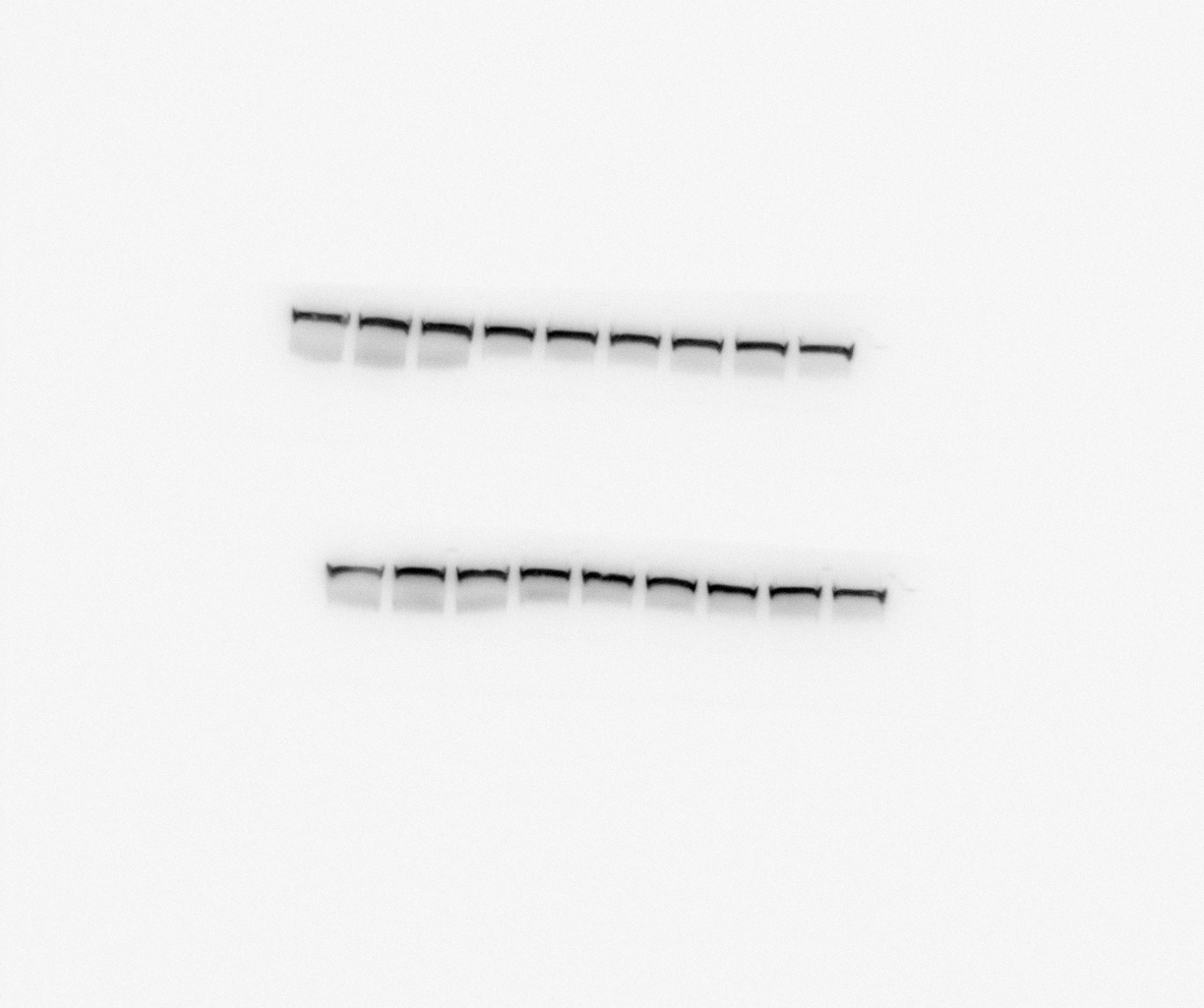

Supplement: Figure 3—source data 2. [file elife-98631-fig3-data2.zip › Figure 3 /3B Vinculin.tif]

Images corresponding to **Figure 3 – figure supplement 1**:

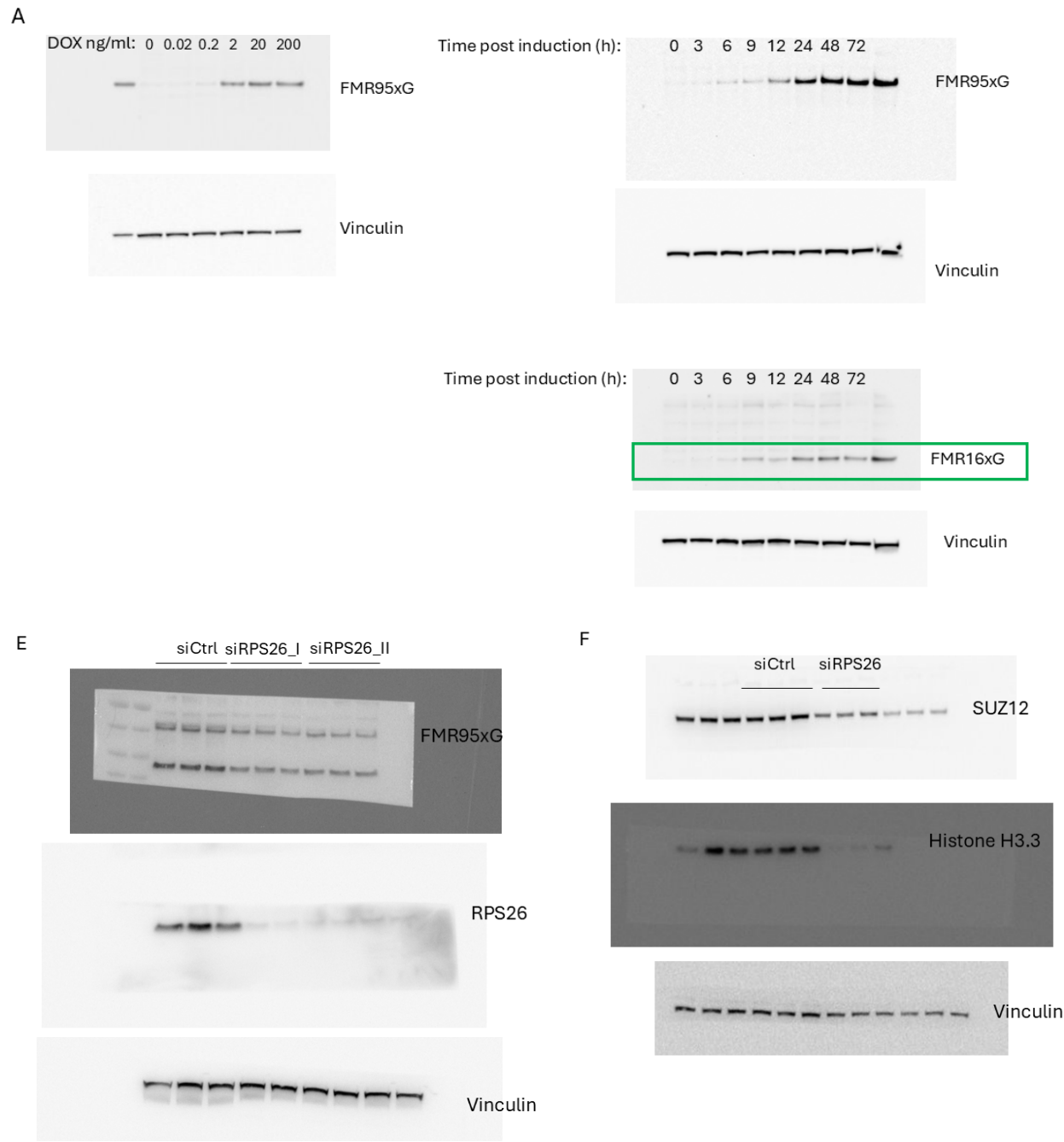

Supplement: Figure 3—figure supplement 1—source data 1. [file elife-98631-fig3-figsupp1-data1.zip › Figure 3 - figure supplement 1 - source data 1.pdf]

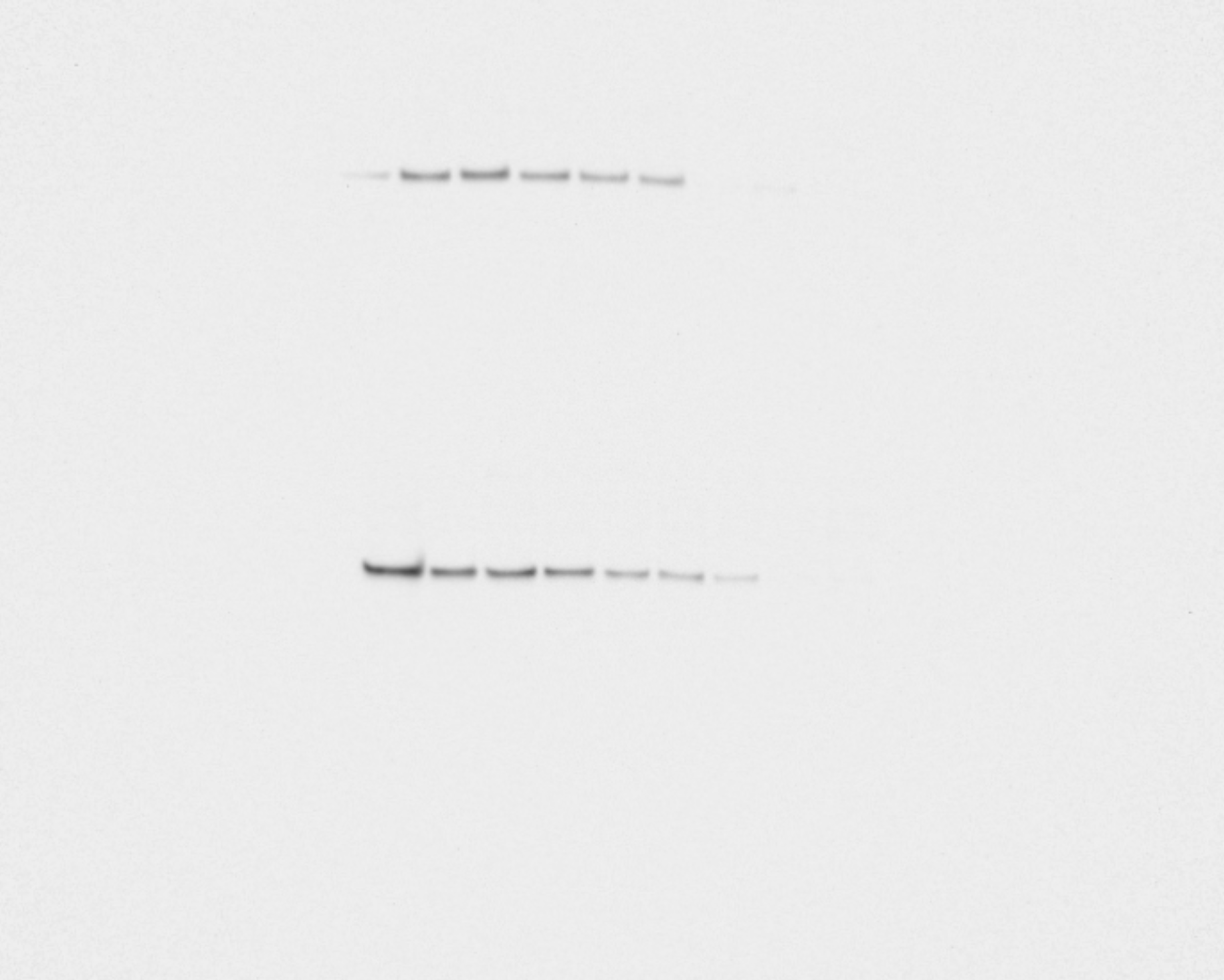

Supplement: Figure 3—figure supplement 1—source data 2. [file elife-98631-fig3-figsupp1-data2.zip › SFig3A FMR16xG - dox time dependency.tif]

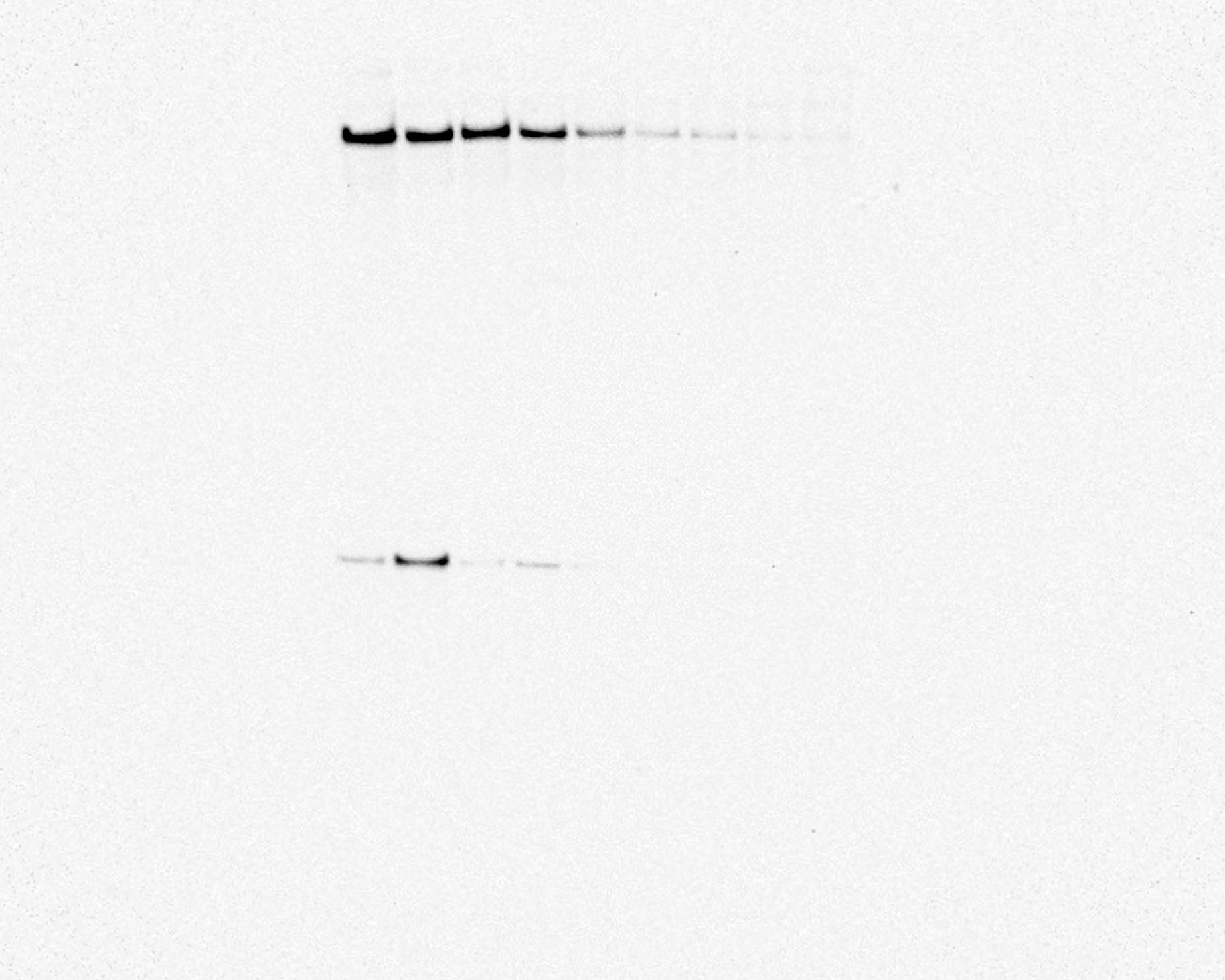

Supplement: Figure 3—figure supplement 1—source data 2. [file elife-98631-fig3-figsupp1-data2.zip › SFig3A FMR95xG - dox time dependency.tif]

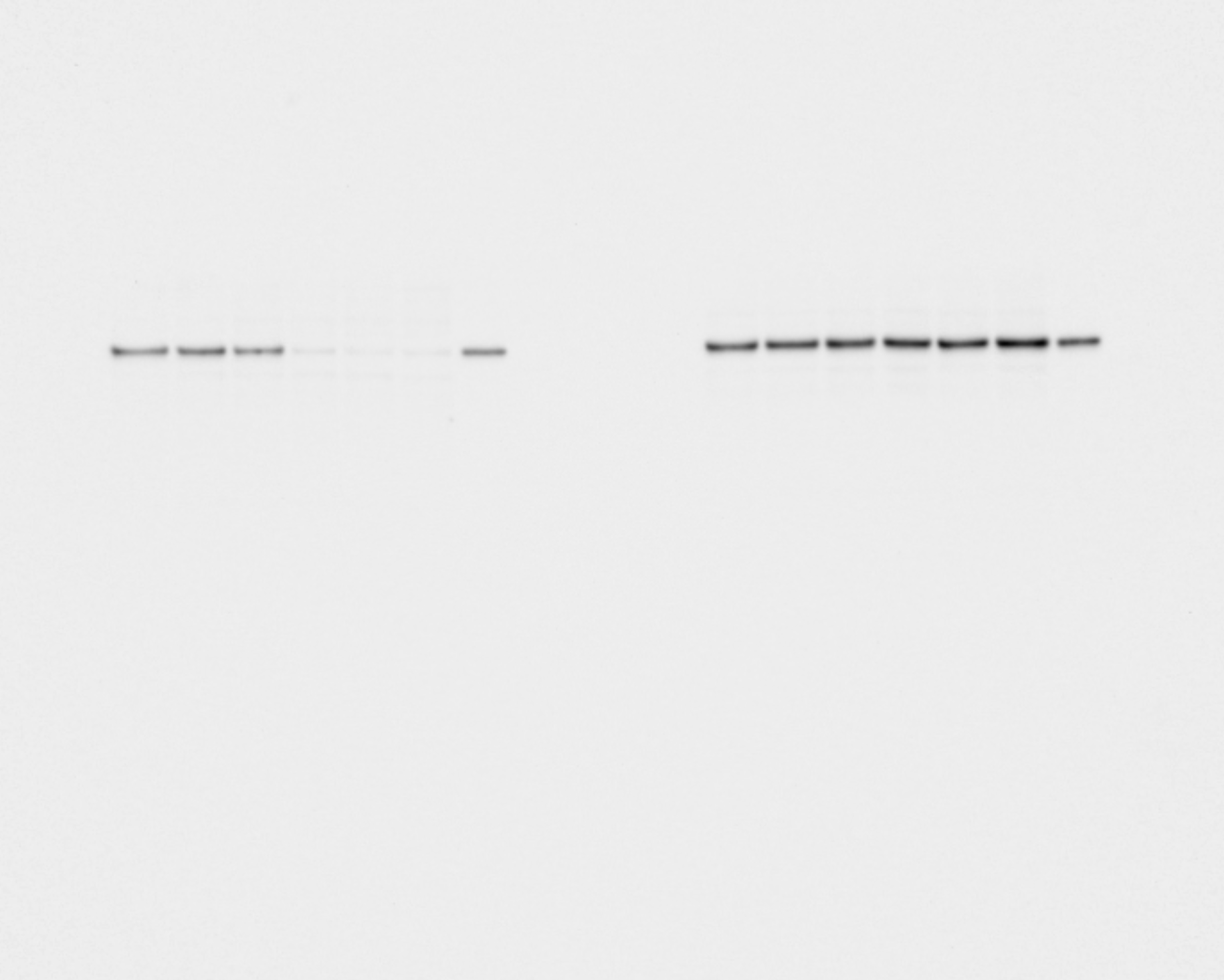

Supplement: Figure 3—figure supplement 1—source data 2. [file elife-98631-fig3-figsupp1-data2.zip › SFig3A FMR95xG - dox conc. dependency.tif]

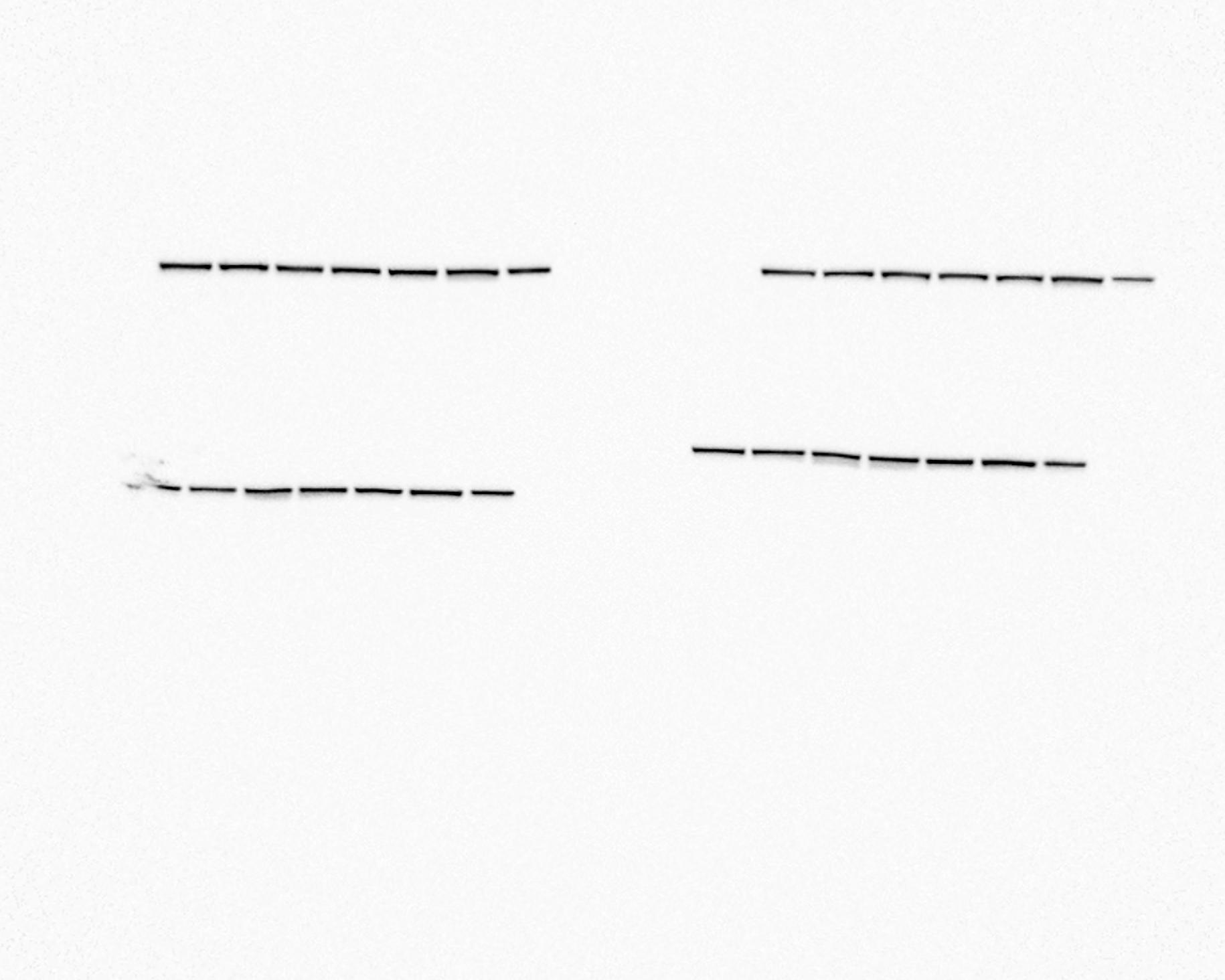

Supplement: Figure 3—figure supplement 1—source data 2. [file elife-98631-fig3-figsupp1-data2.zip › SFig3A Vinculin - dox conc. dependency.tif]

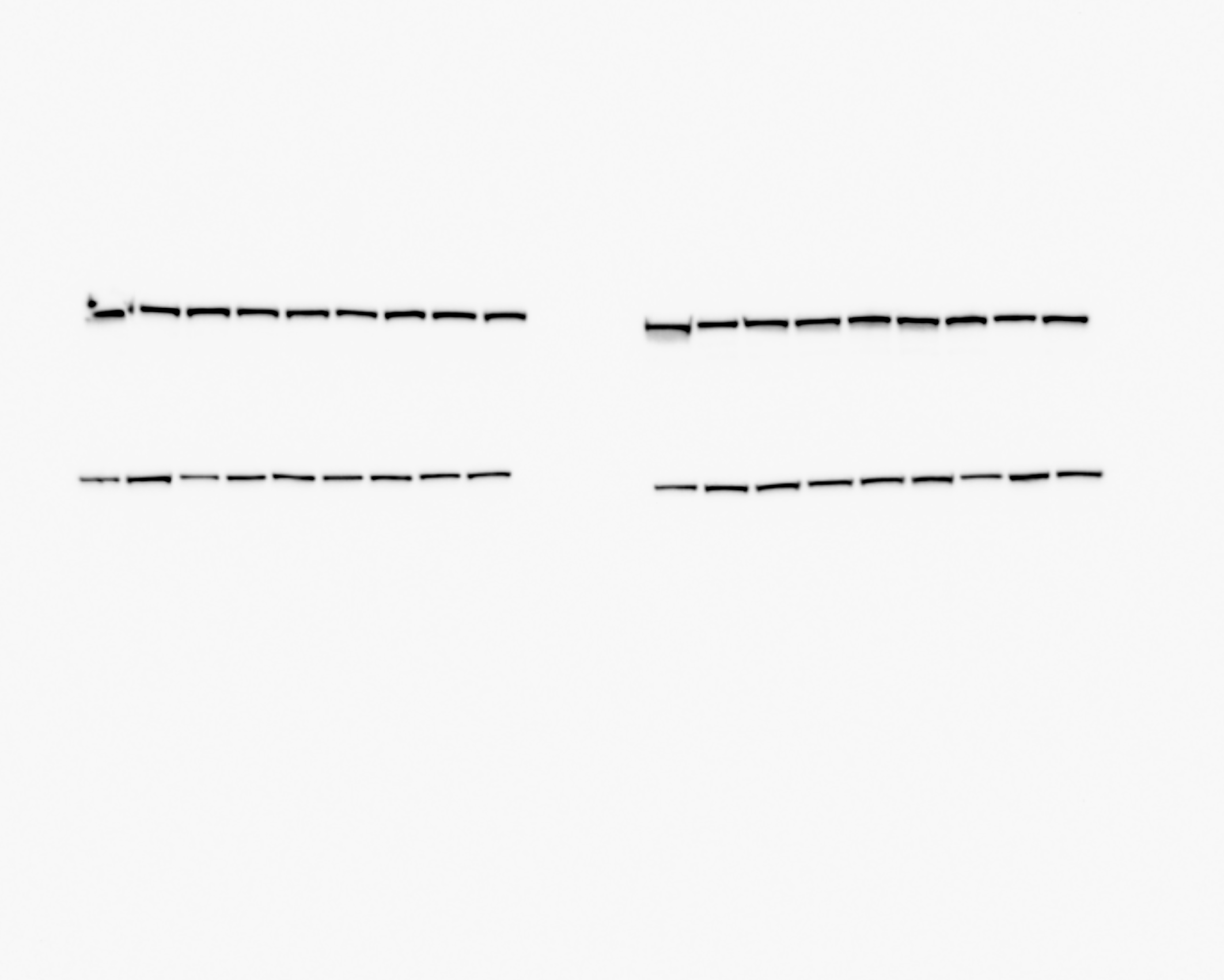

Supplement: Figure 3—figure supplement 1—source data 2. [file elife-98631-fig3-figsupp1-data2.zip › SFig3A Vinculin - dox time dependency.tif]

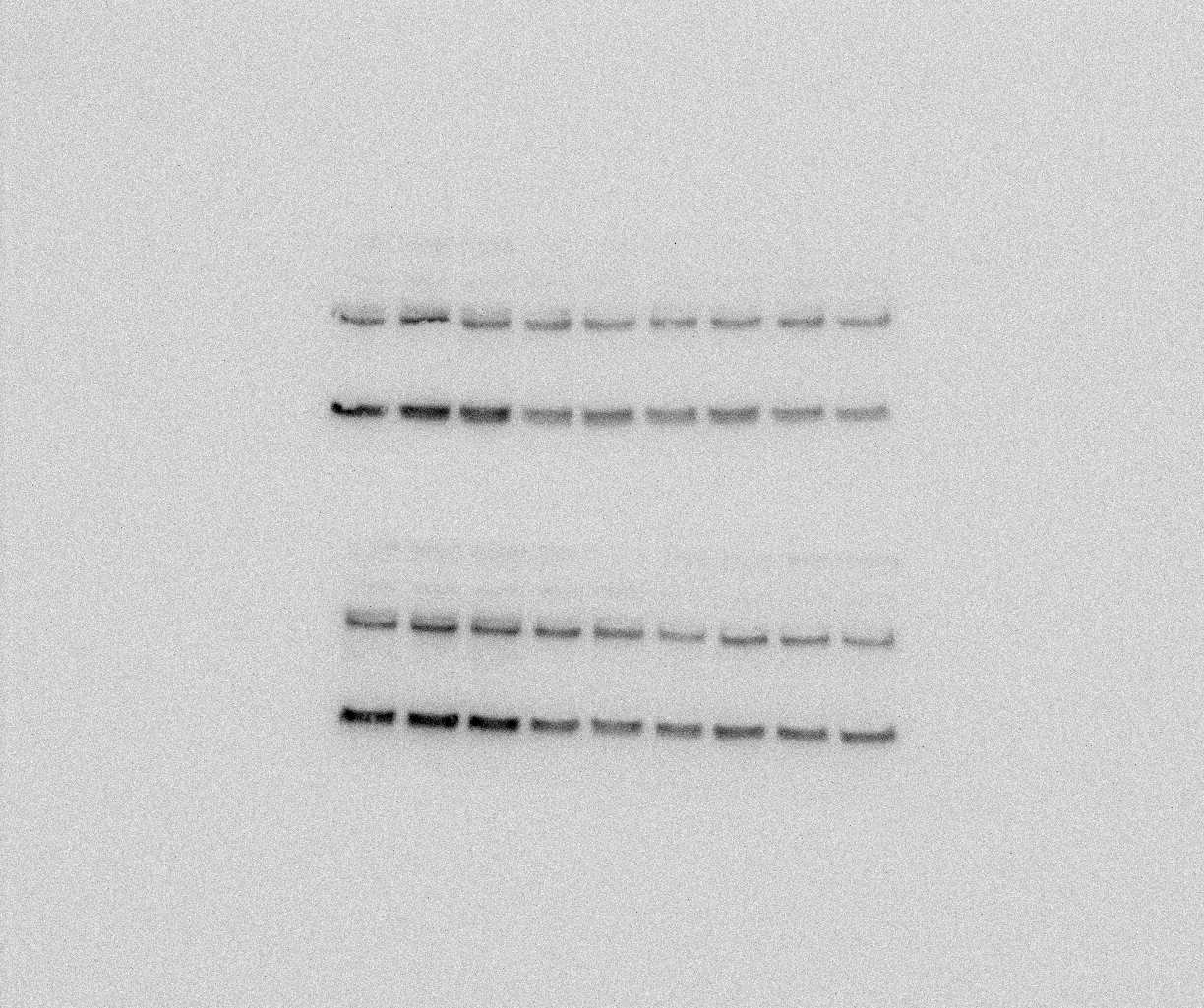

Supplement: Figure 3—figure supplement 1—source data 2. [file elife-98631-fig3-figsupp1-data2.zip › SFig3E FMR95xG.tif]

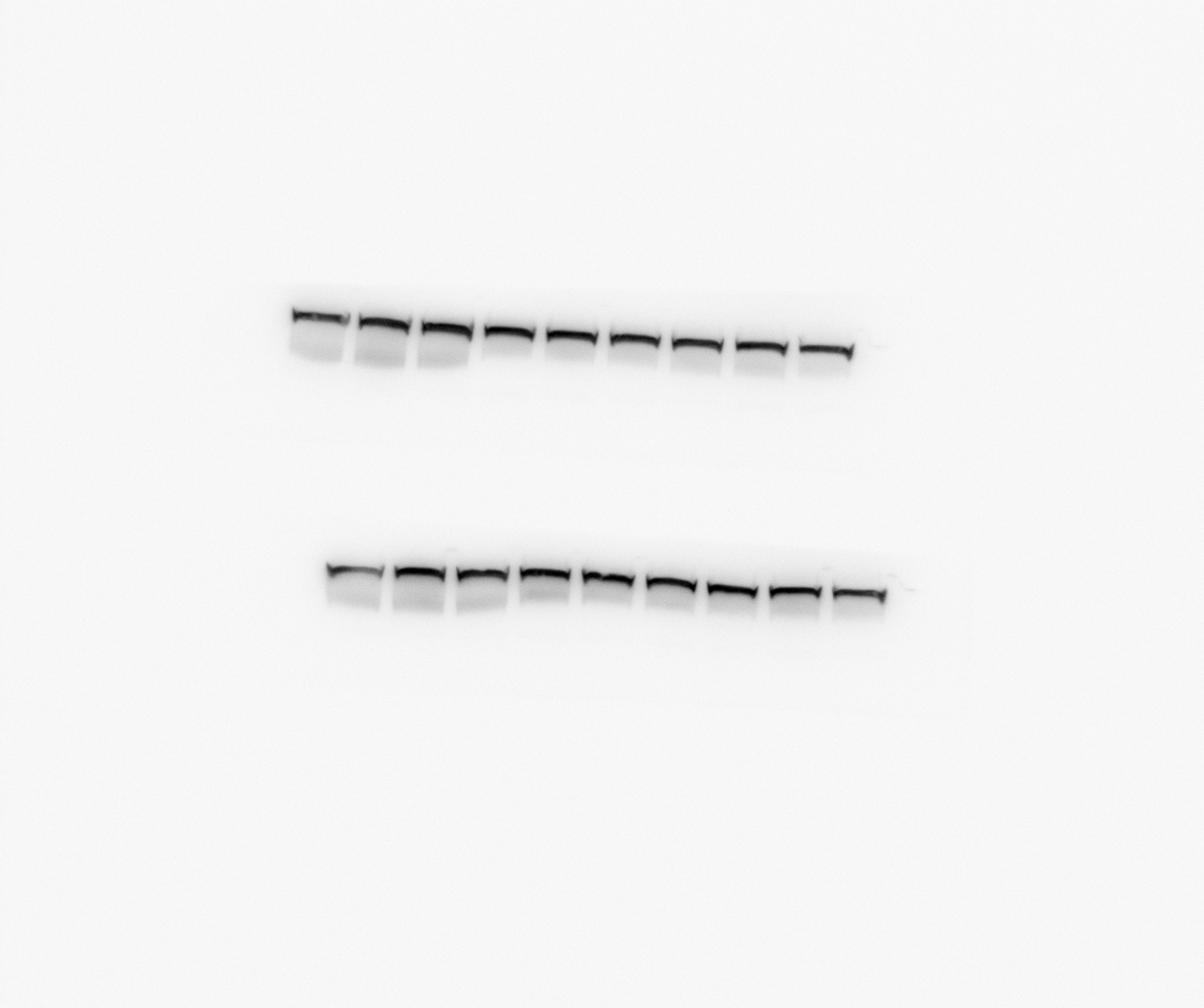

Supplement: Figure 3—figure supplement 1—source data 2. [file elife-98631-fig3-figsupp1-data2.zip › SFig3E Vinculin.tif]

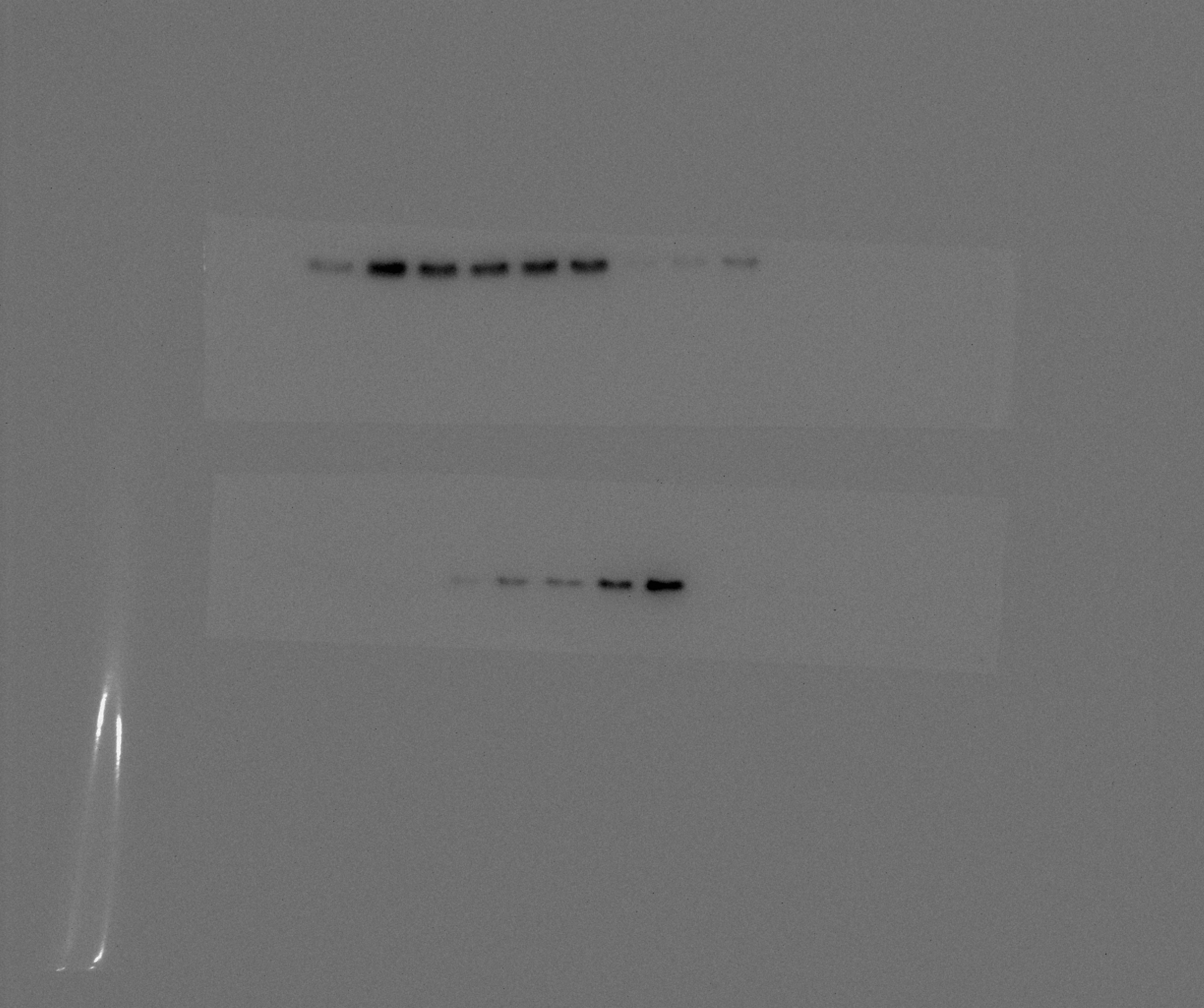

Supplement: Figure 3—figure supplement 1—source data 2. [file elife-98631-fig3-figsupp1-data2.zip › SFig3F H3.3.tif]

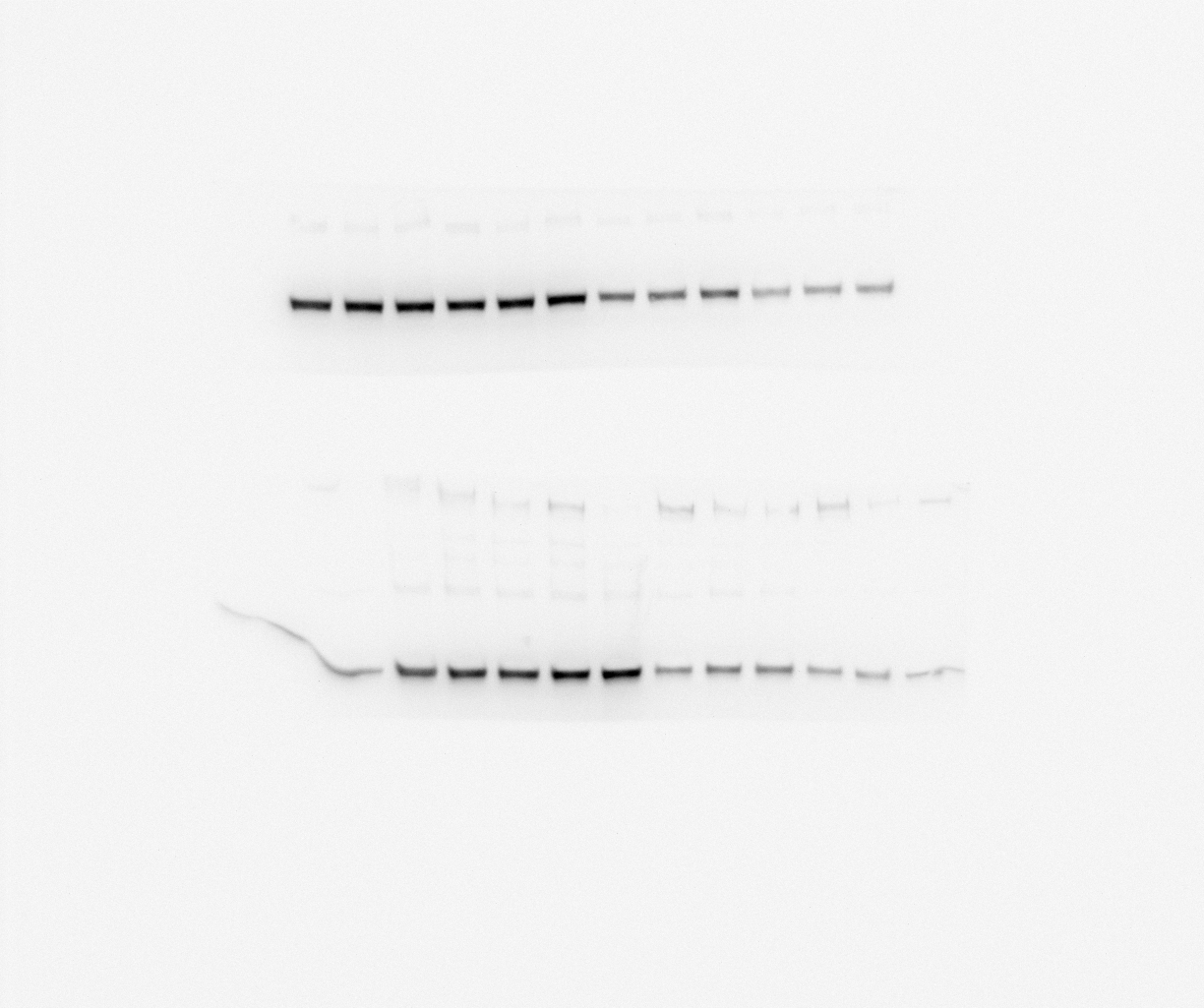

Supplement: Figure 3—figure supplement 1—source data 2. [file elife-98631-fig3-figsupp1-data2.zip › SFig3F SUZ12.tif]

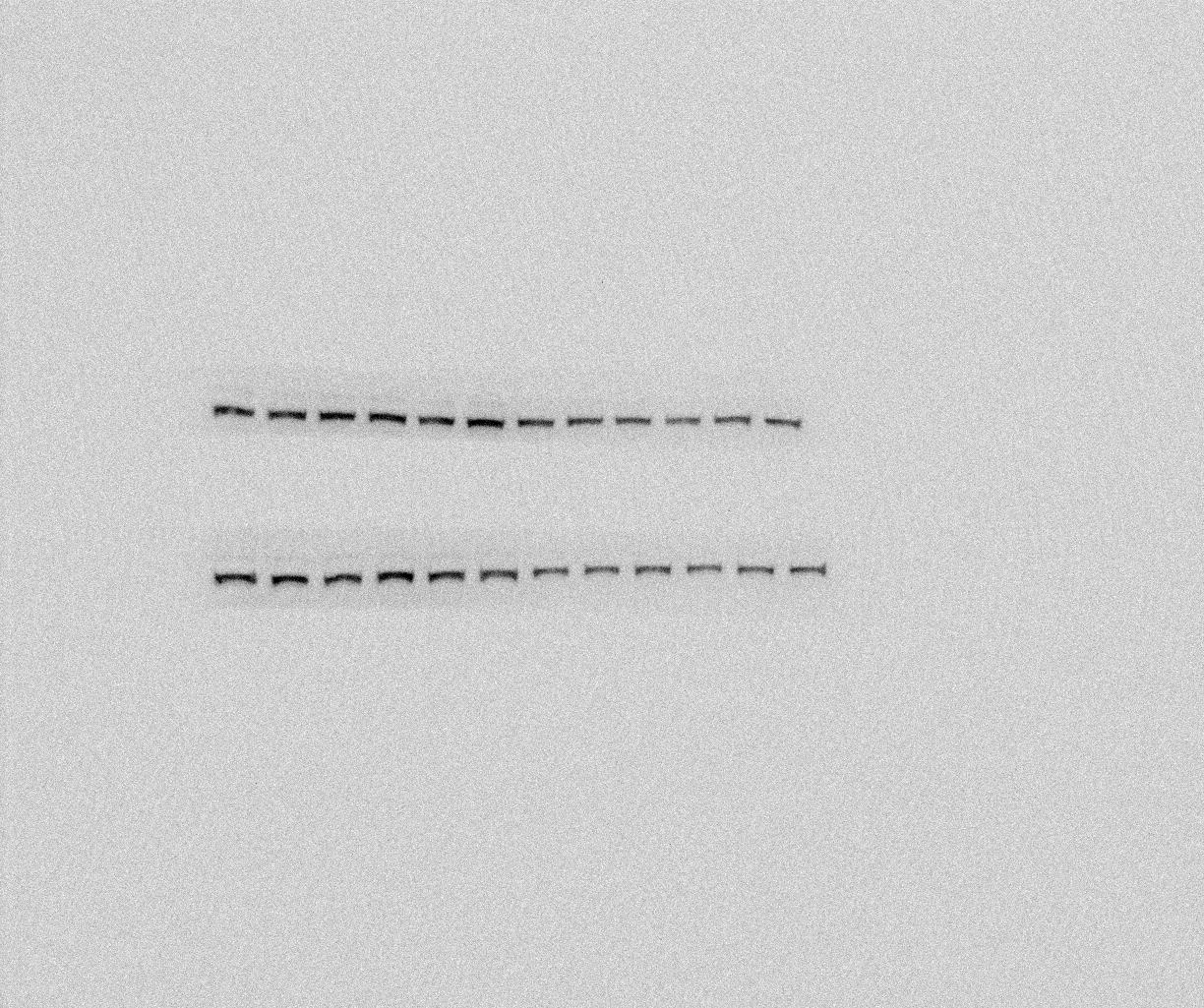

Supplement: Figure 3—figure supplement 1—source data 2. [file elife-98631-fig3-figsupp1-data2.zip › SFig3F Vinculin.tif]

Images corresponding to **Figure 4**:

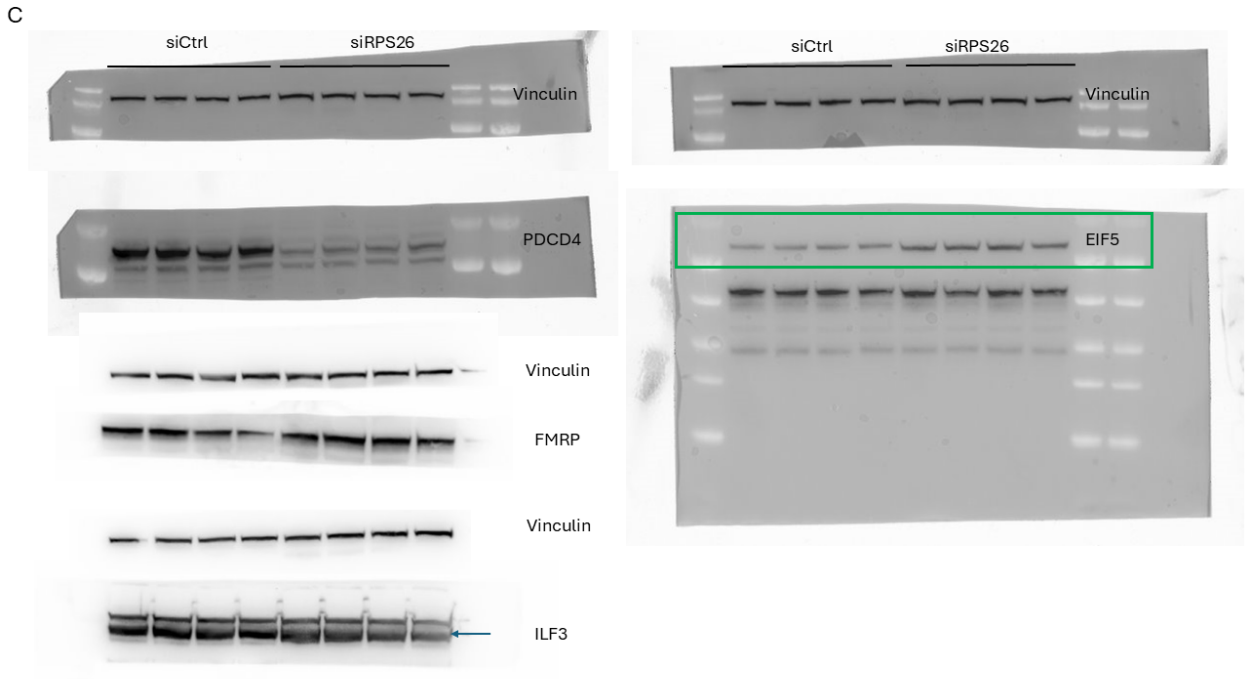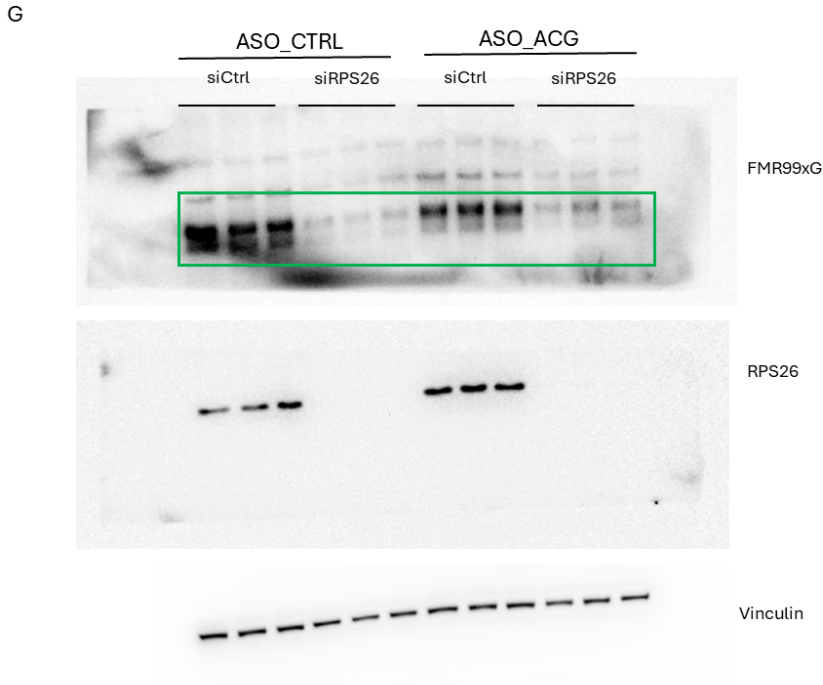

Supplement: Figure 4—source data 1. [file elife-98631-fig4-data1.zip › Figure 4 - source data 1.pdf]

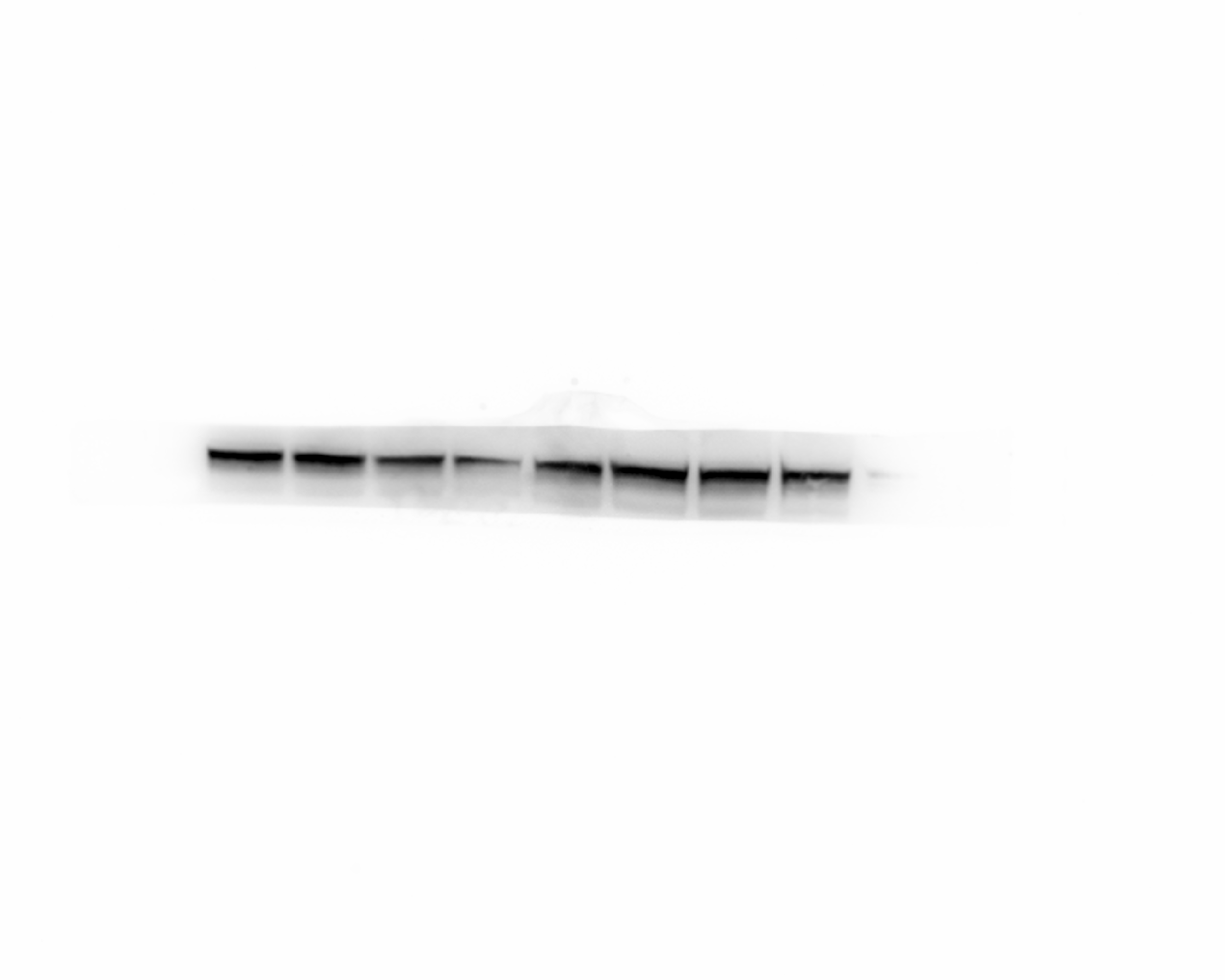

Supplement: Figure 4—source data 2. [file elife-98631-fig4-data2.zip › Figure 4 /4C FMRP.tif]

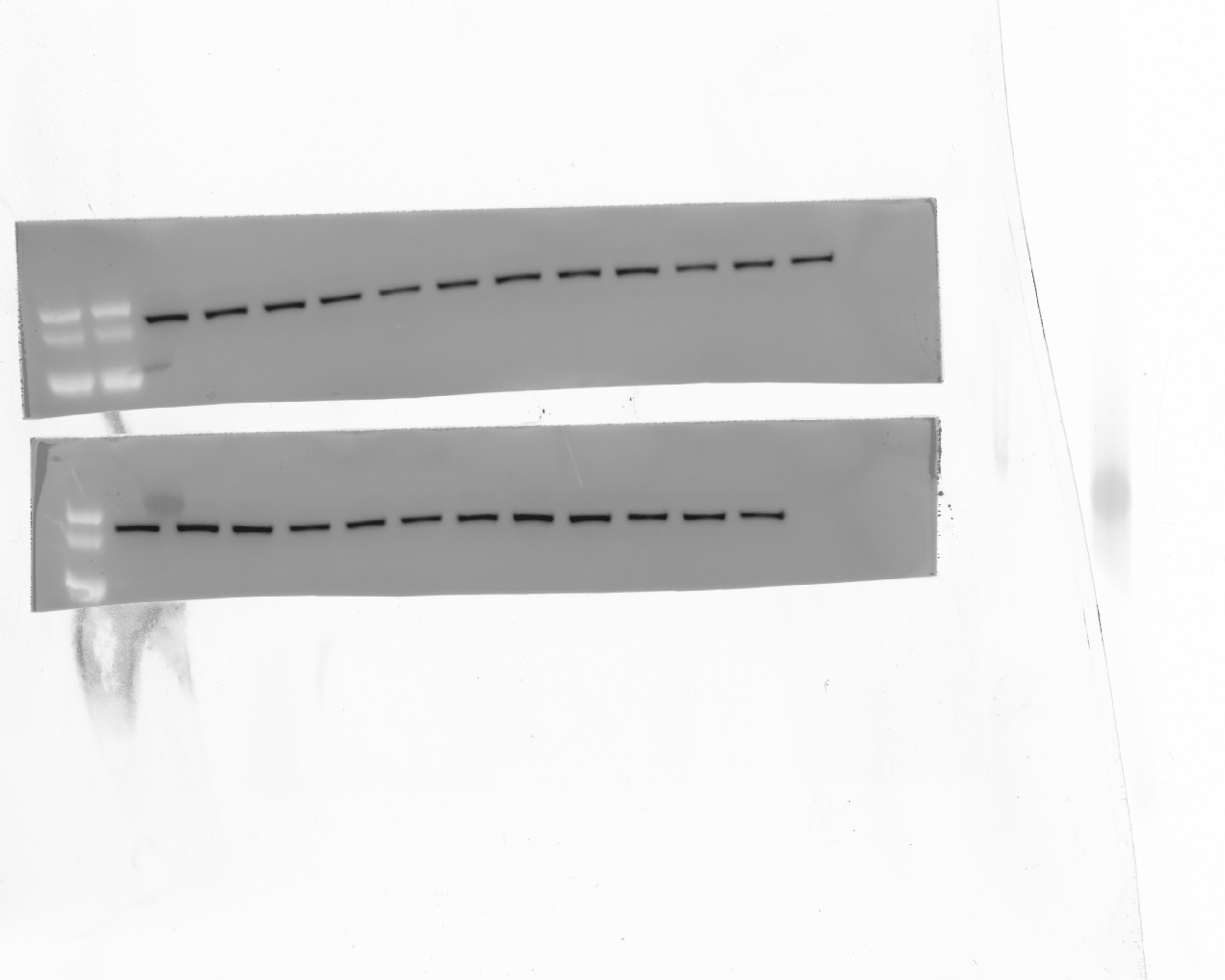

Supplement: Figure 4—source data 2. [file elife-98631-fig4-data2.zip › Figure 4 /4G Vinculin.tif]

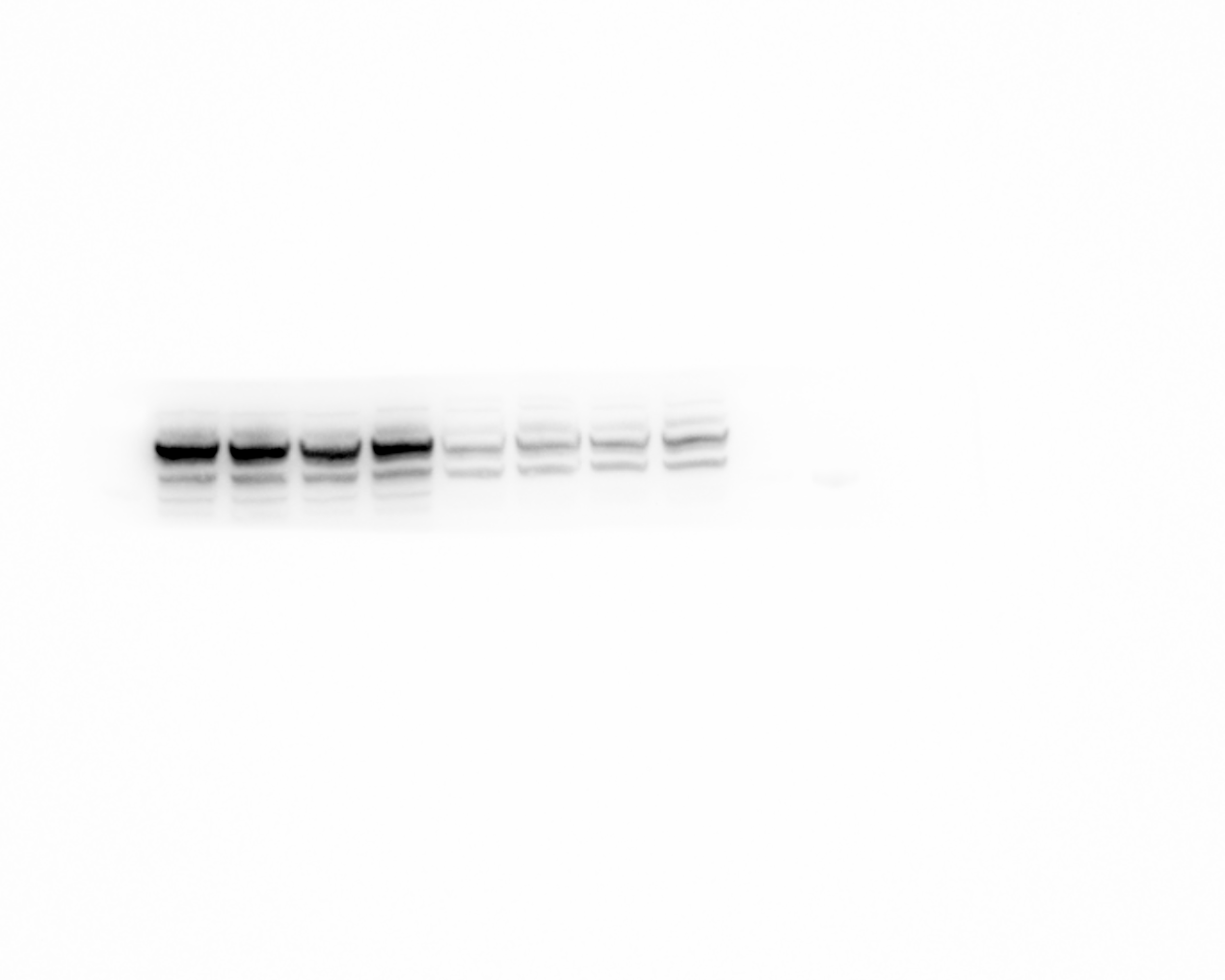

Supplement: Figure 4—source data 2. [file elife-98631-fig4-data2.zip › Figure 4 /4C PDCD4.tif]

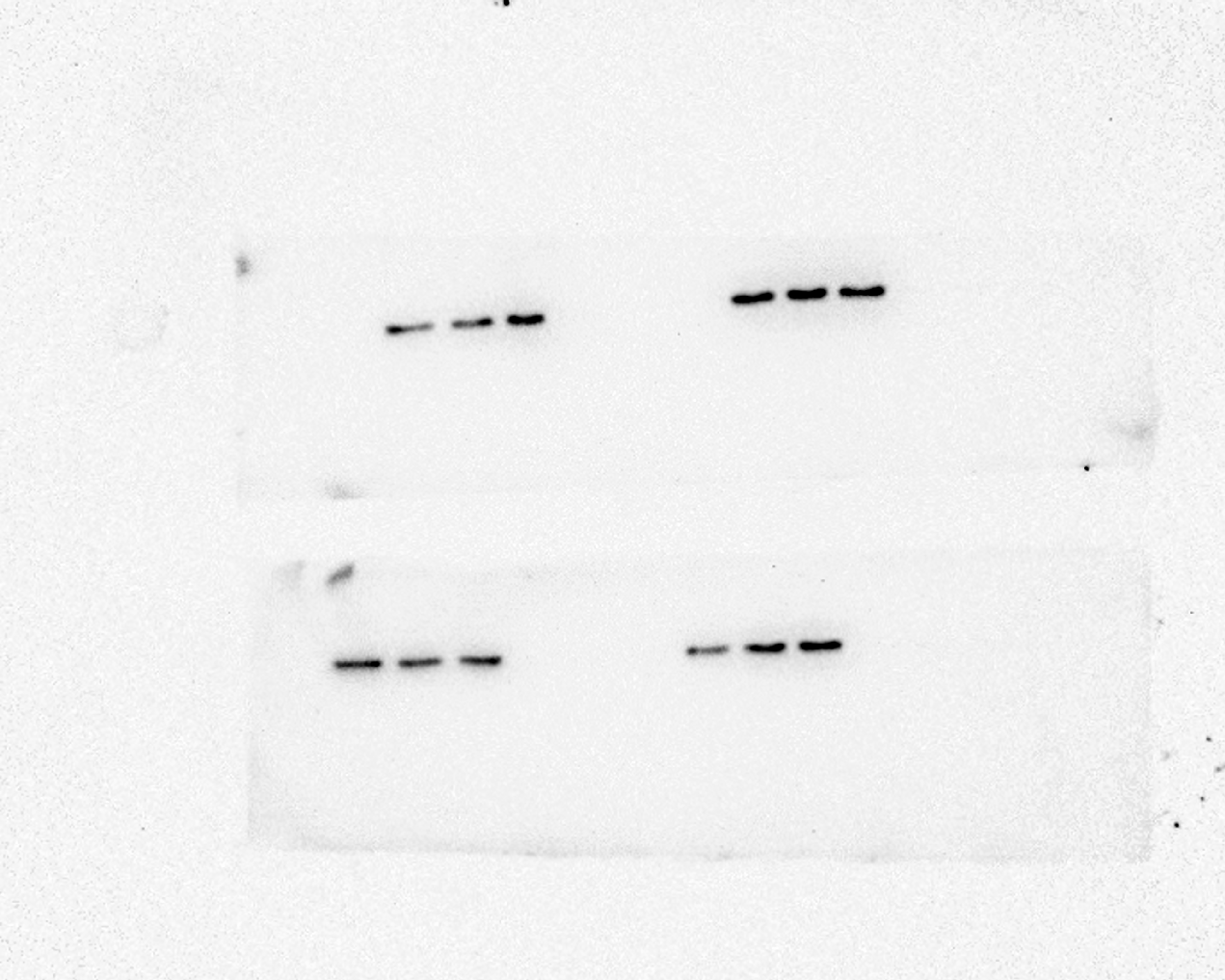

Supplement: Figure 4—source data 2. [file elife-98631-fig4-data2.zip › Figure 4 /4G RPS26.tif]

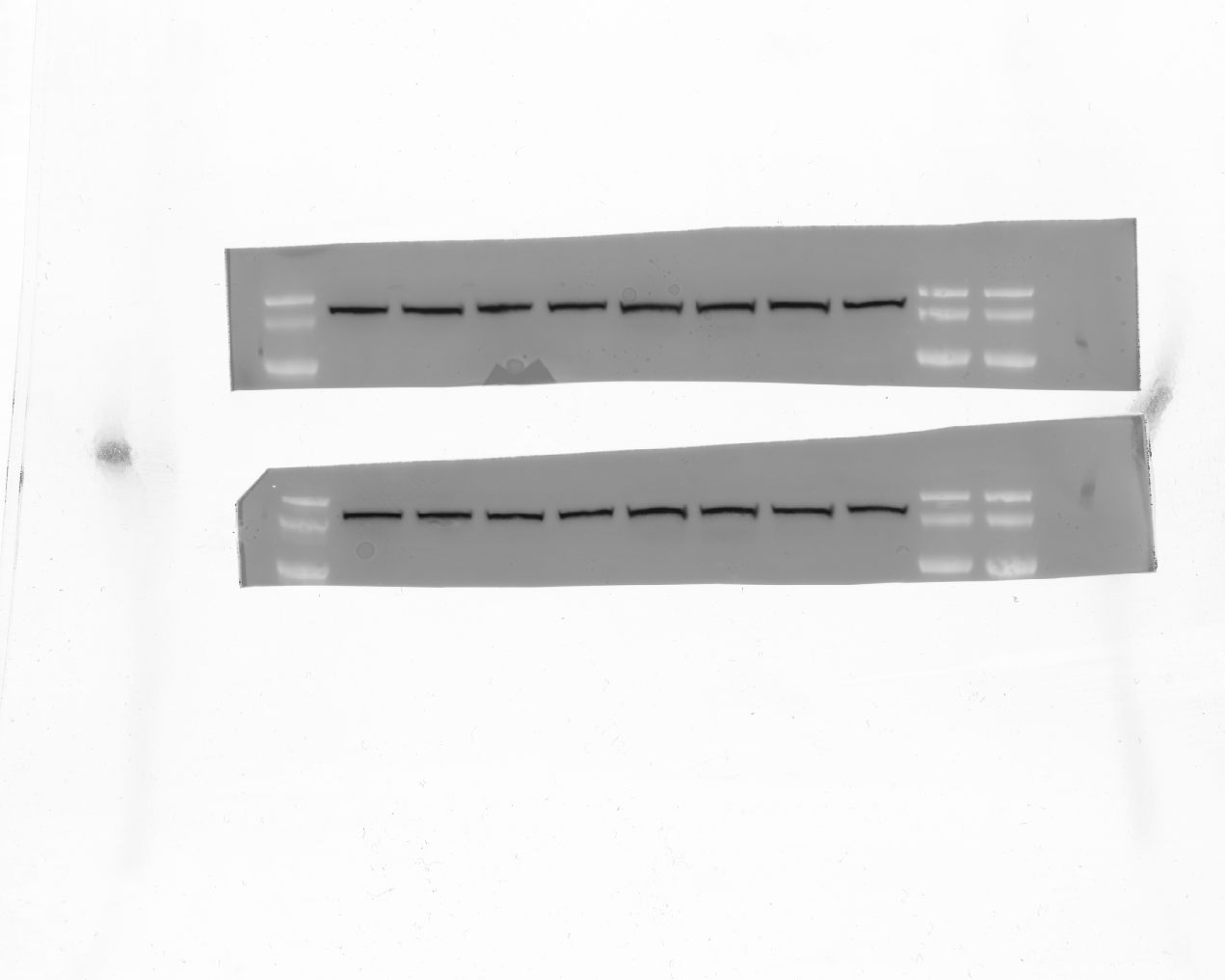

Supplement: Figure 4—source data 2. [file elife-98631-fig4-data2.zip › Figure 4 /4C Vinculin to PDCD4 and EIF5.tif]

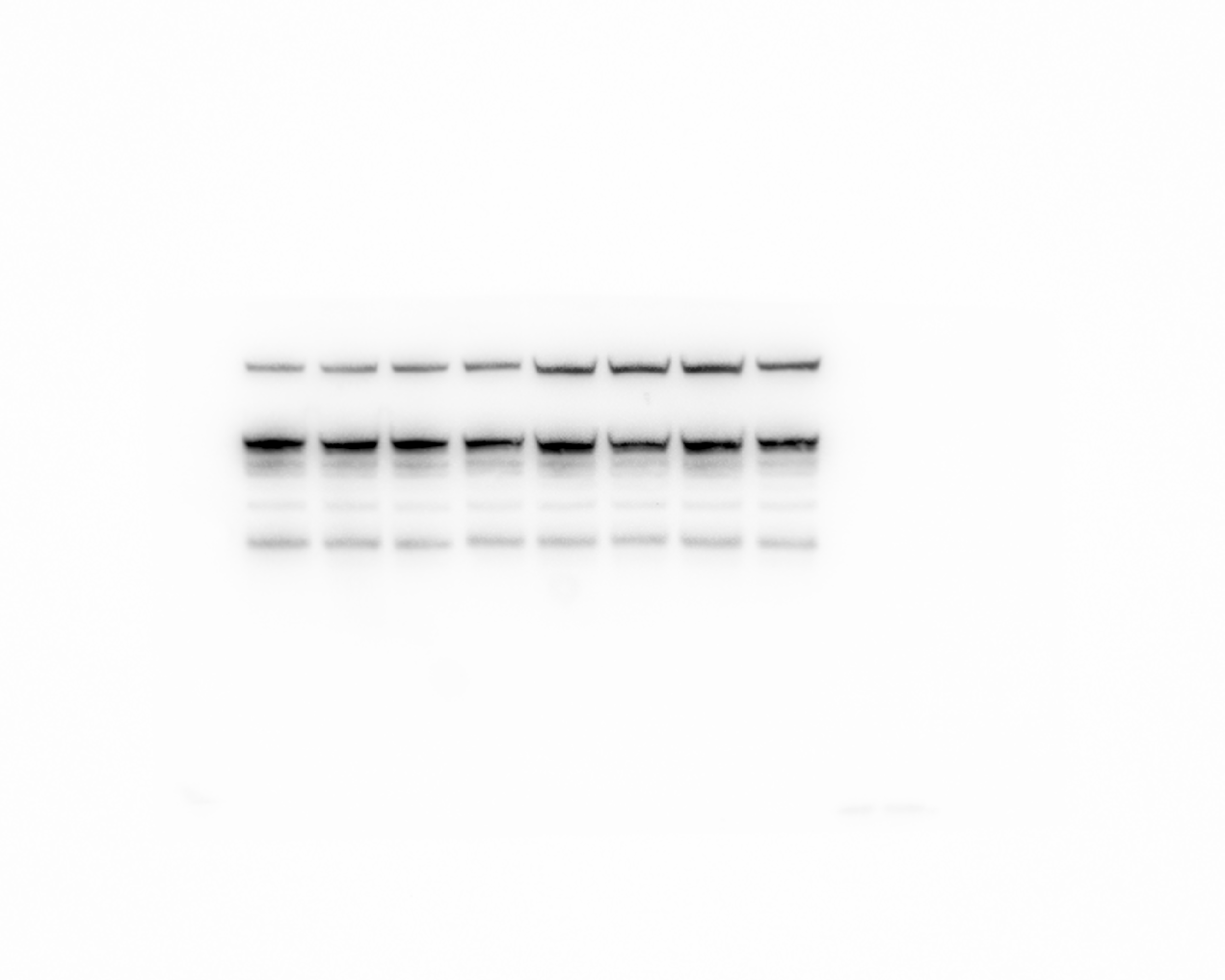

Supplement: Figure 4—source data 2. [file elife-98631-fig4-data2.zip › Figure 4 /4C EIF5.tif]

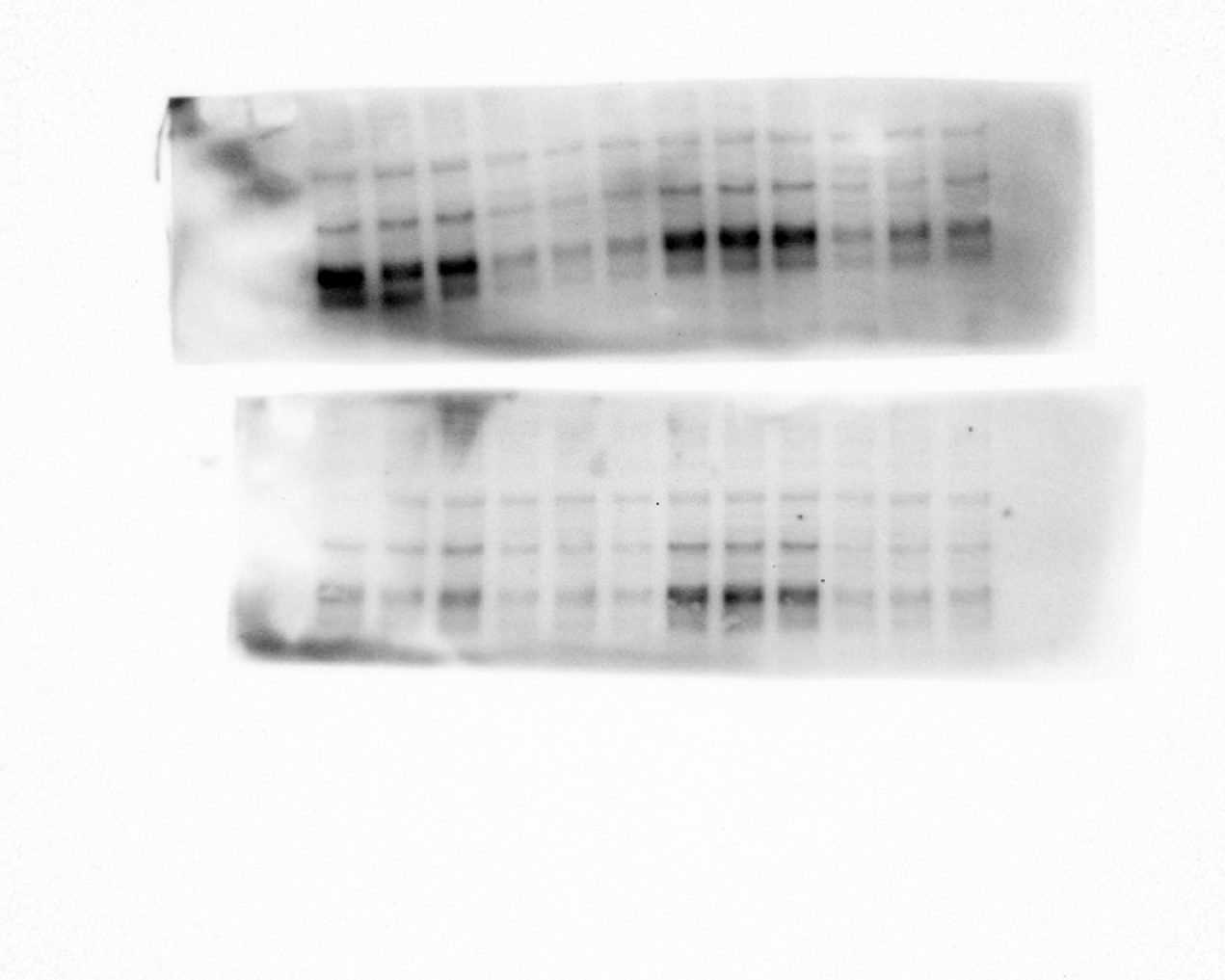

Supplement: Figure 4—source data 2. [file elife-98631-fig4-data2.zip › Figure 4 /4G FMR99xG.tif]

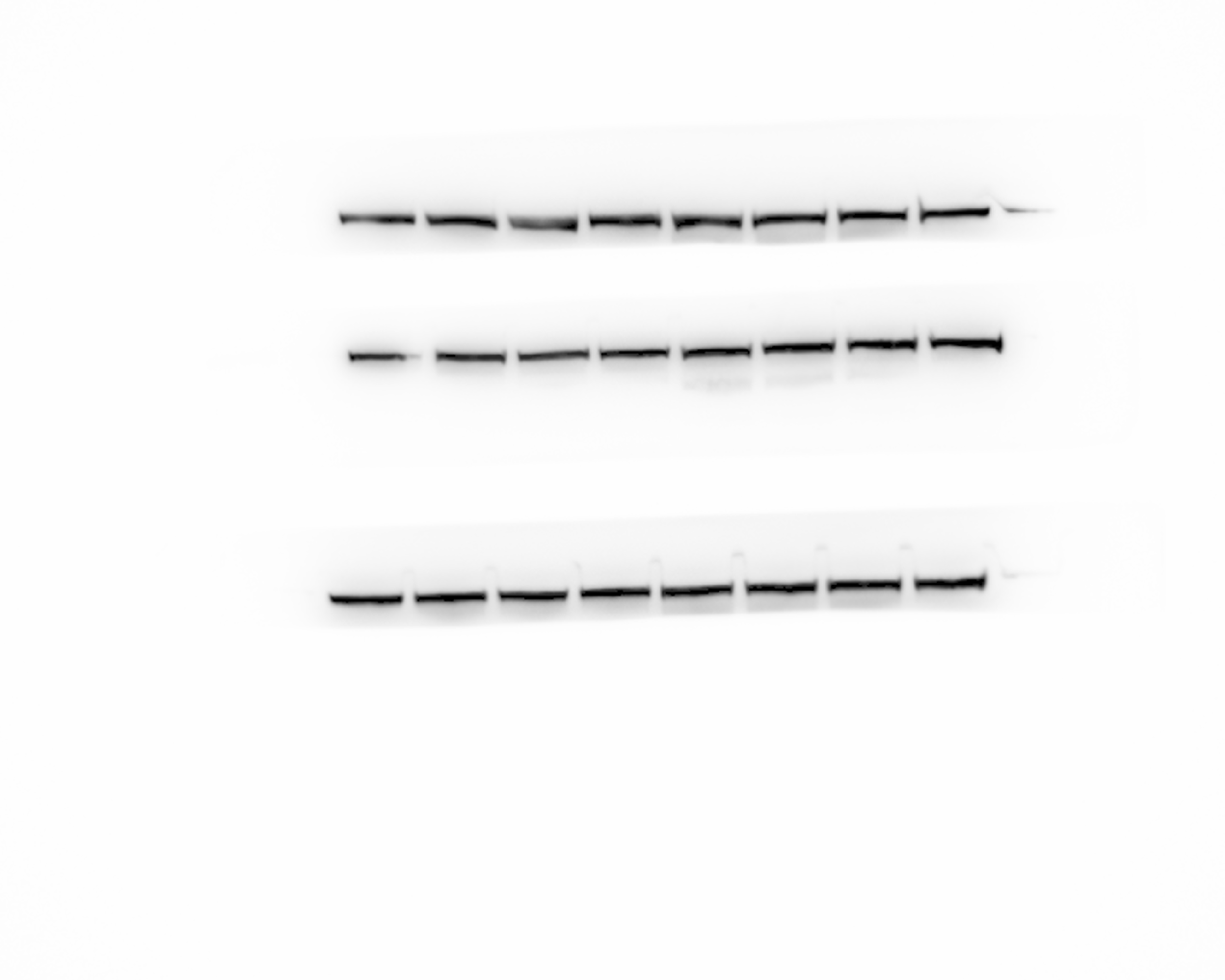

Supplement: Figure 4—source data 2. [file elife-98631-fig4-data2.zip › Figure 4 /4C Vinculin to FMRP and ILF3.tif]

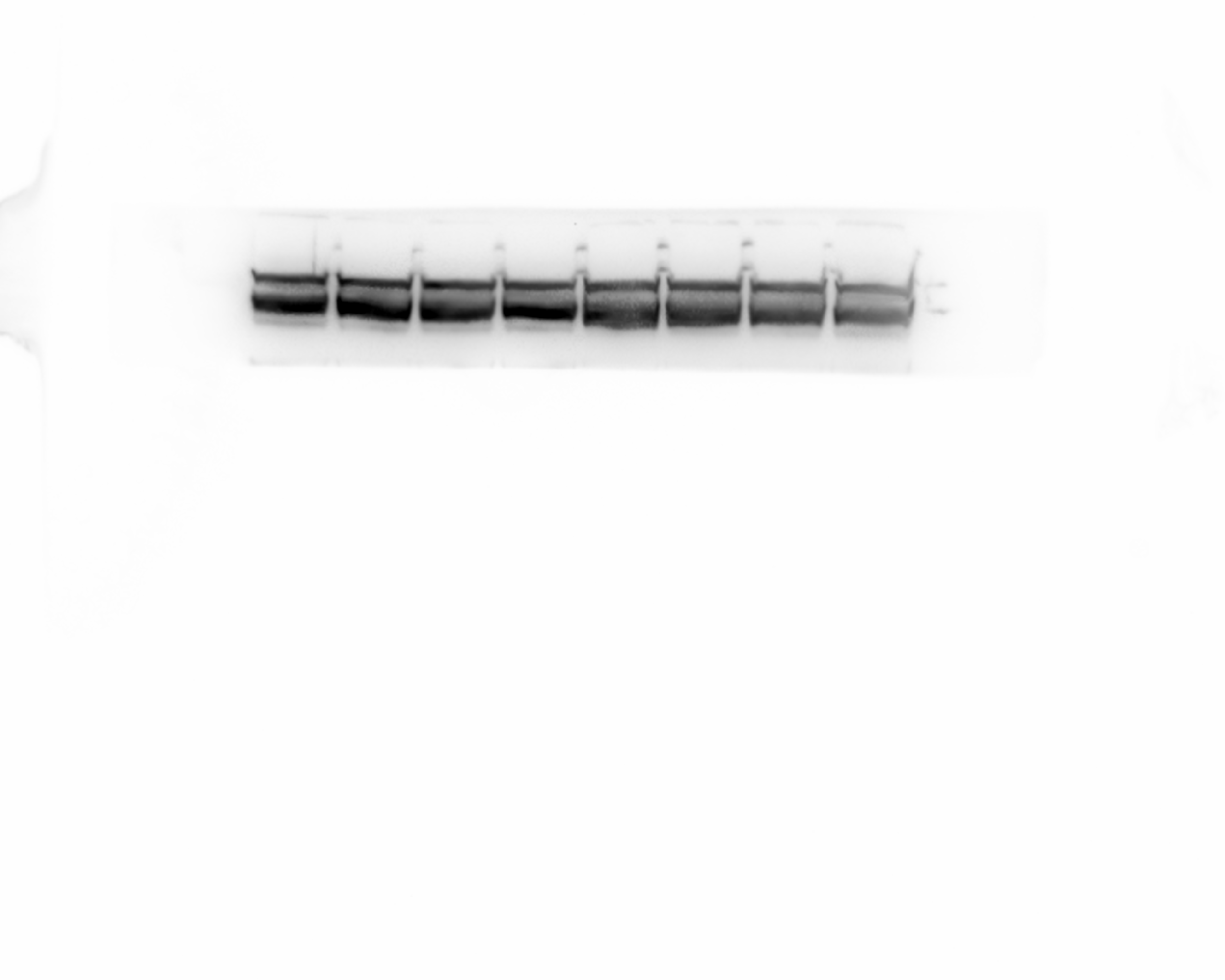

Supplement: Figure 4—source data 2. [file elife-98631-fig4-data2.zip › Figure 4 /4C ILF3.tif]

Images corresponding to **Figure 4 – figure supplement 1**:

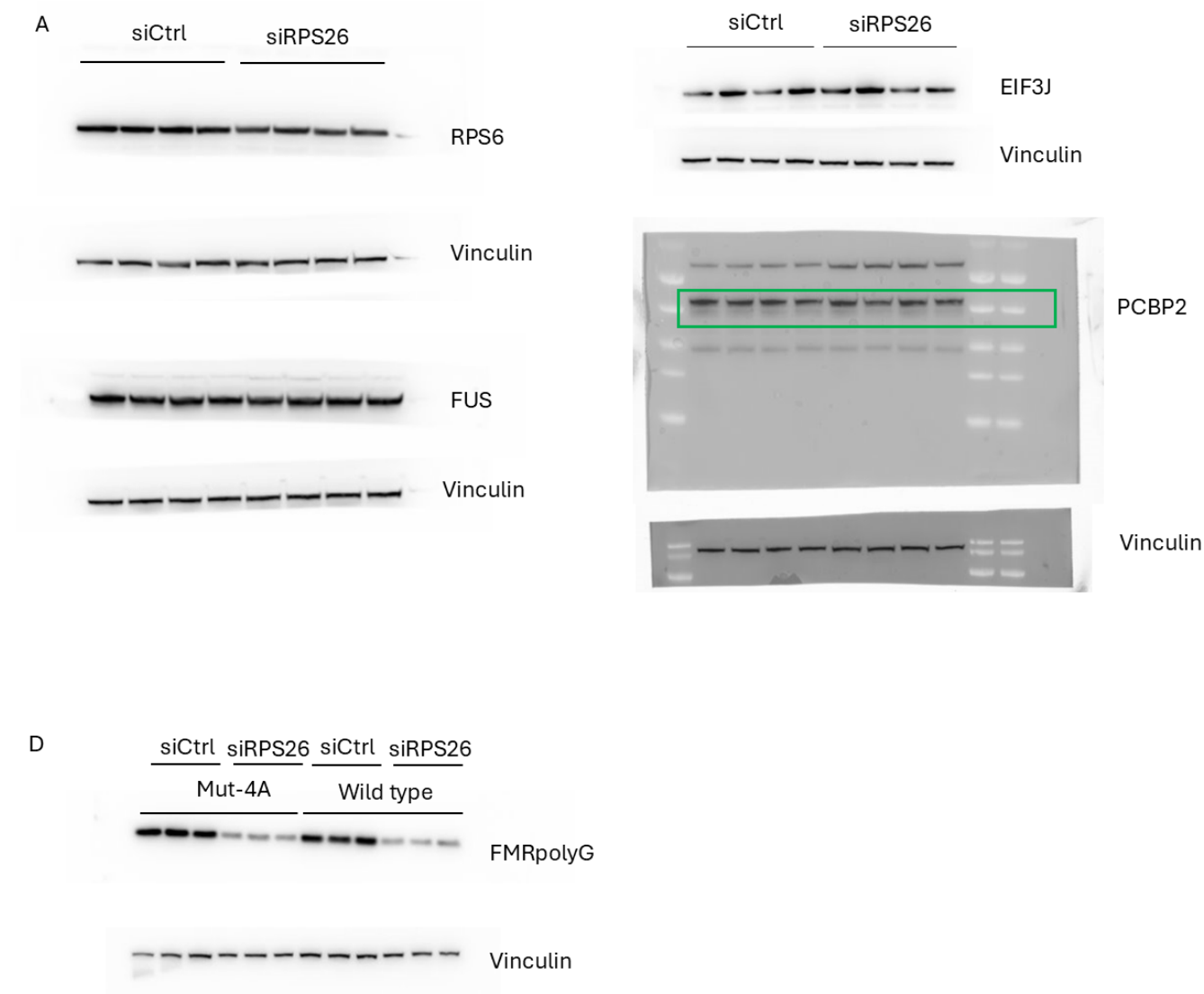

Supplement: Figure 4—figure supplement 1—source data 1. [file elife-98631-fig4-figsupp1-data1.zip › Images corresponding to Figure 4.pdf]

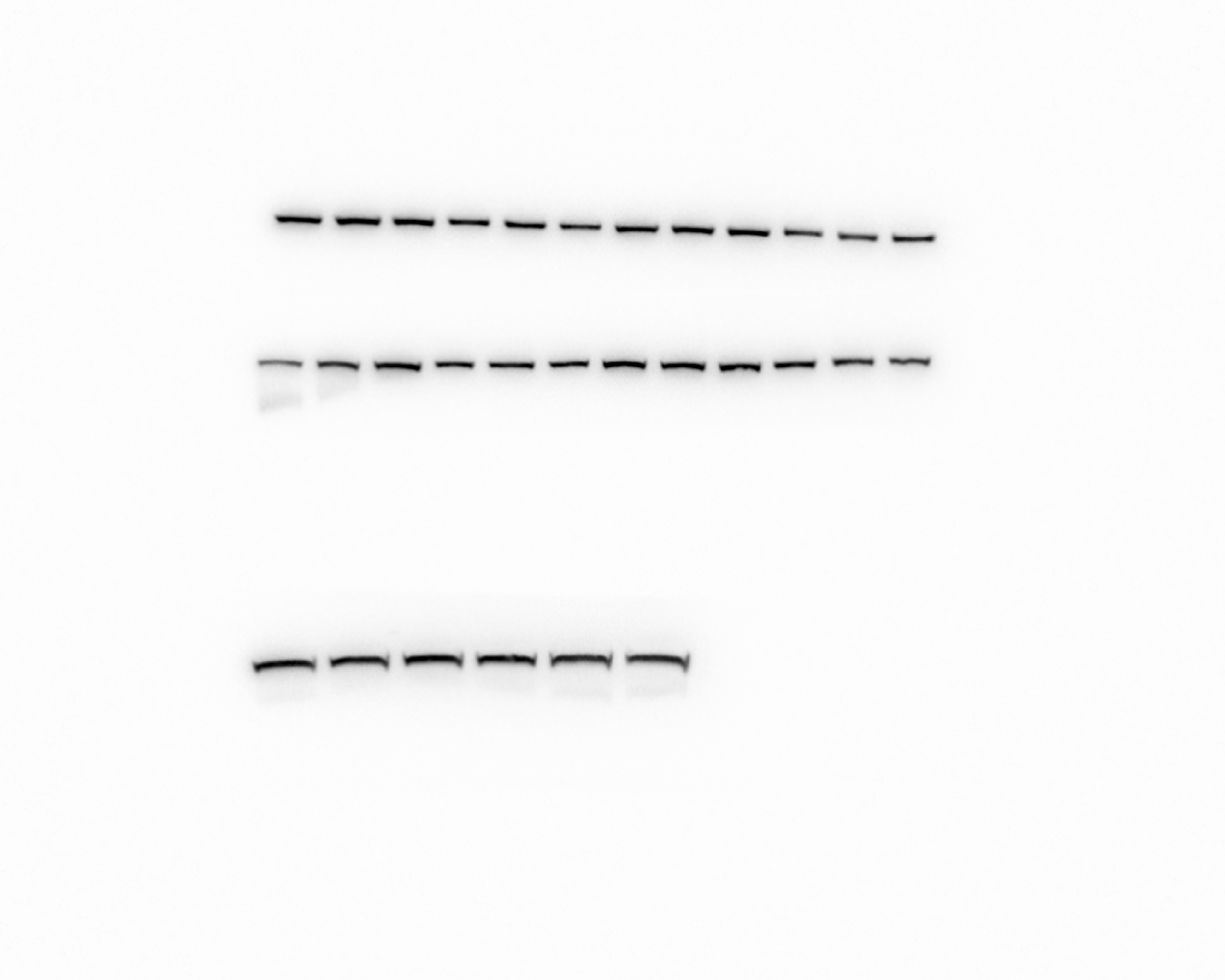

Supplement: Figure 4—figure supplement 1—source data 2. [file elife-98631-fig4-figsupp1-data2.zip › SFig4D Vinculin.tif]

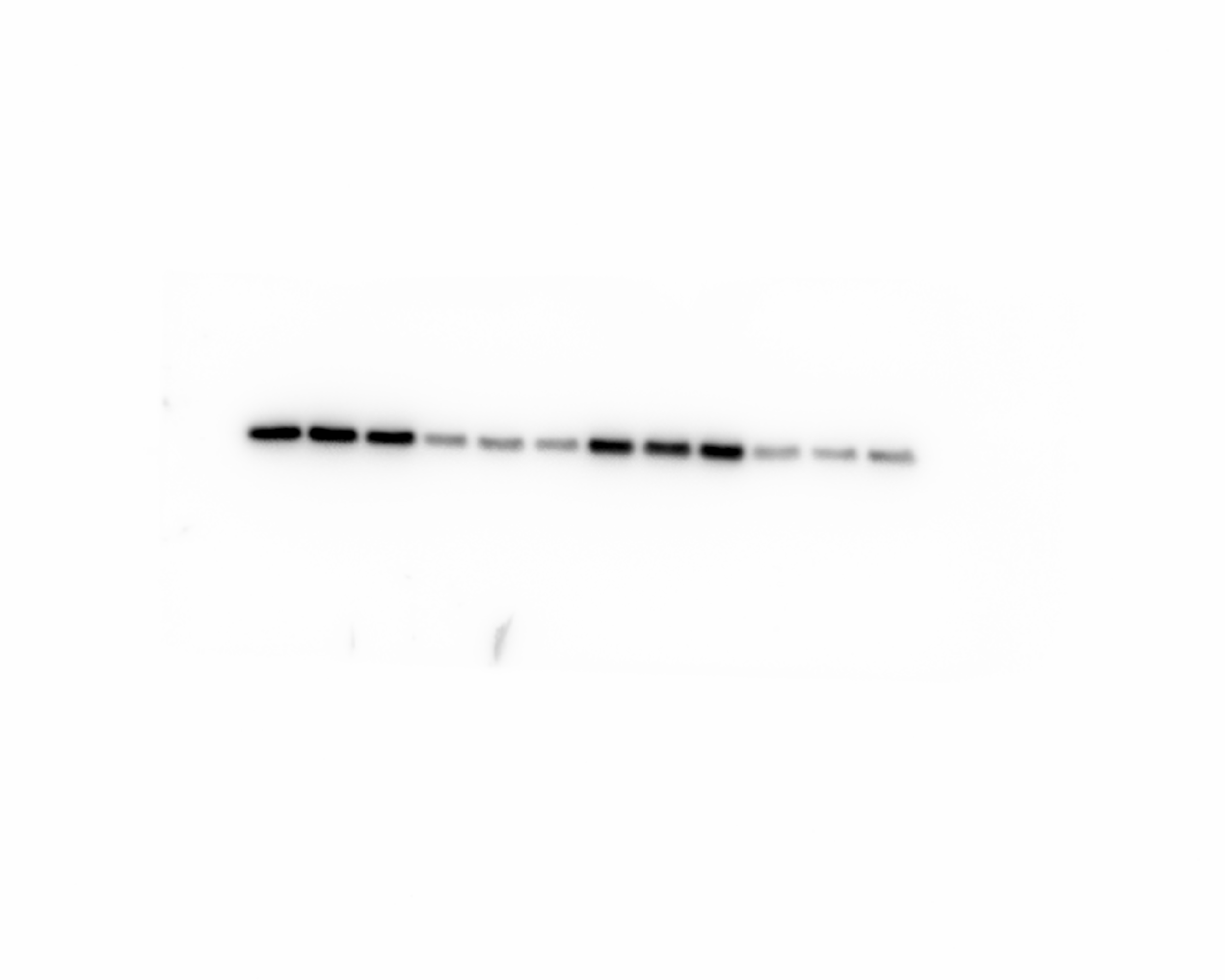

Supplement: Figure 4—figure supplement 1—source data 2. [file elife-98631-fig4-figsupp1-data2.zip › SFig4D FMRpolyG.tif]

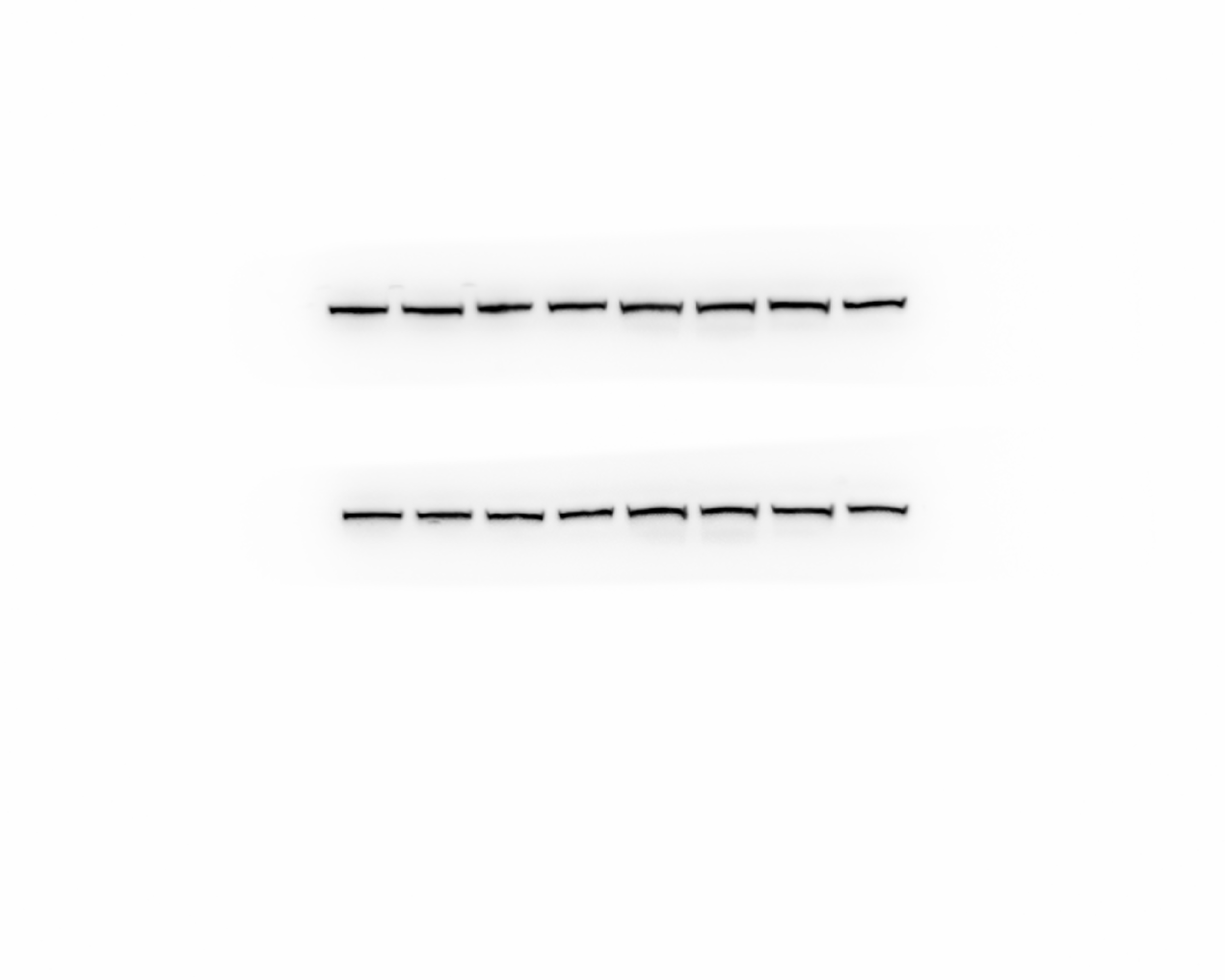

Supplement: Figure 4—figure supplement 1—source data 2. [file elife-98631-fig4-figsupp1-data2.zip › SFig4A Vinculin do PBC2B.tif]

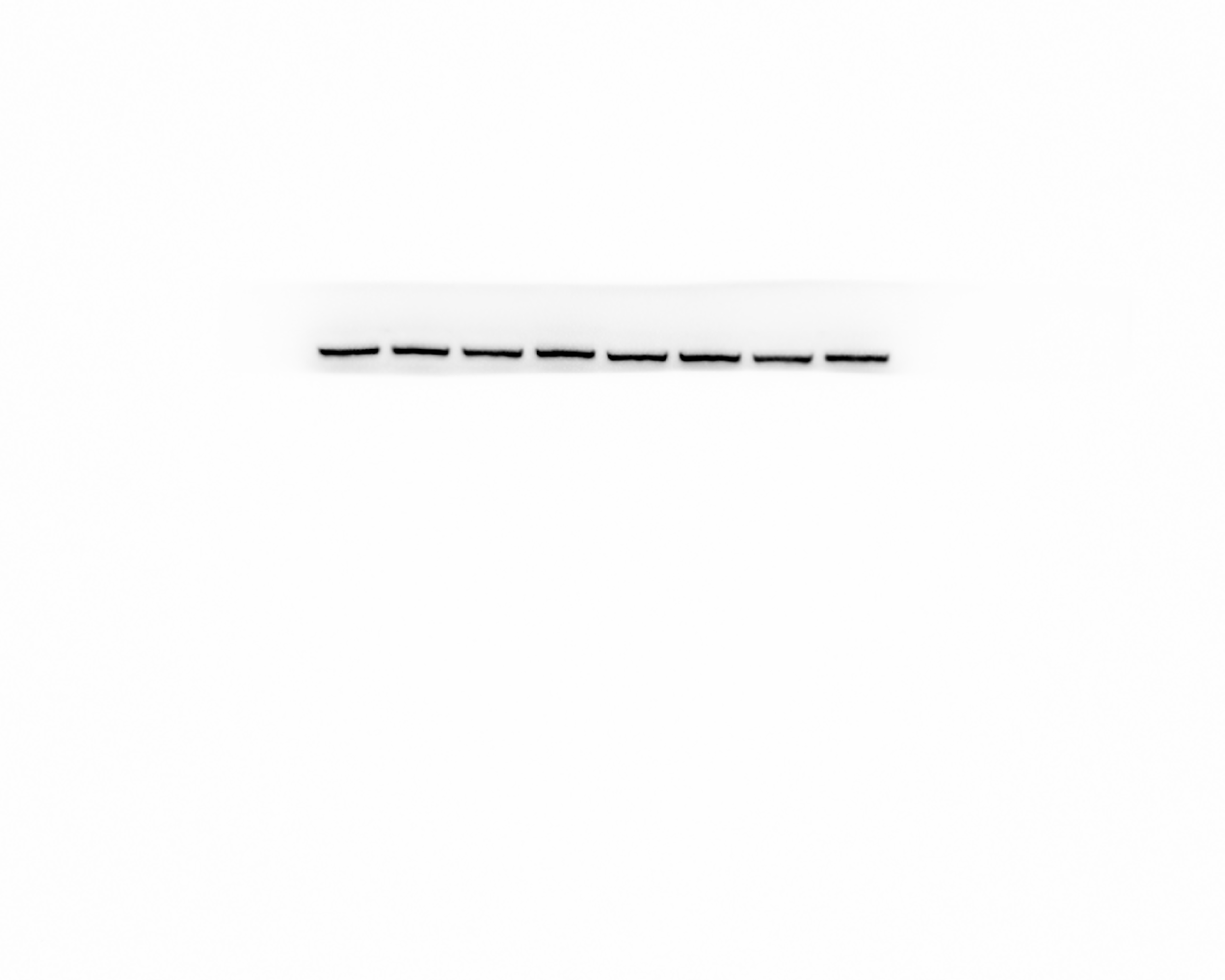

Supplement: Figure 4—figure supplement 1—source data 2. [file elife-98631-fig4-figsupp1-data2.zip › SFig4A Vinculin do EIF3J.tif]

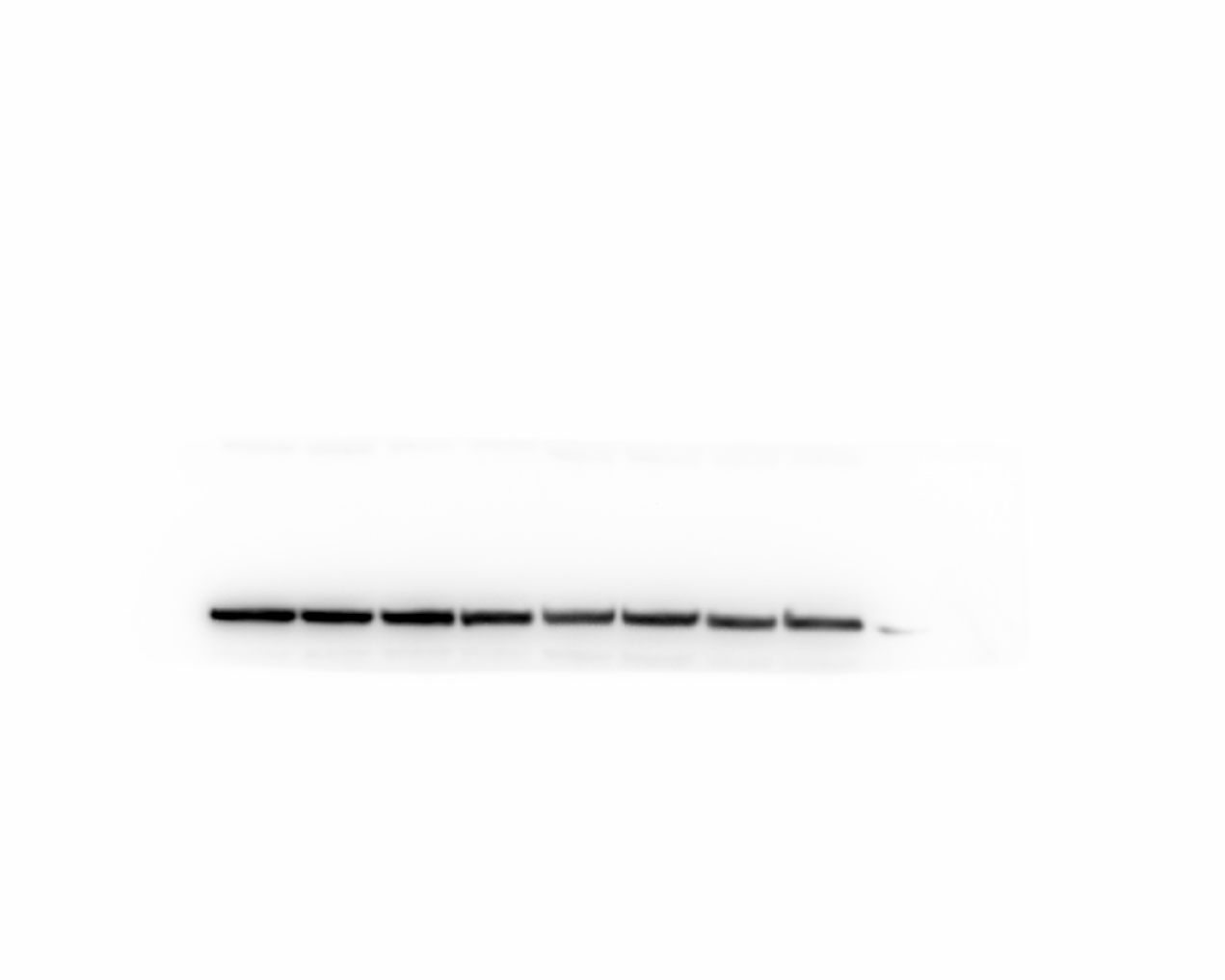

Supplement: Figure 4—figure supplement 1—source data 2. [file elife-98631-fig4-figsupp1-data2.zip › SFig4A RPS6.tif]

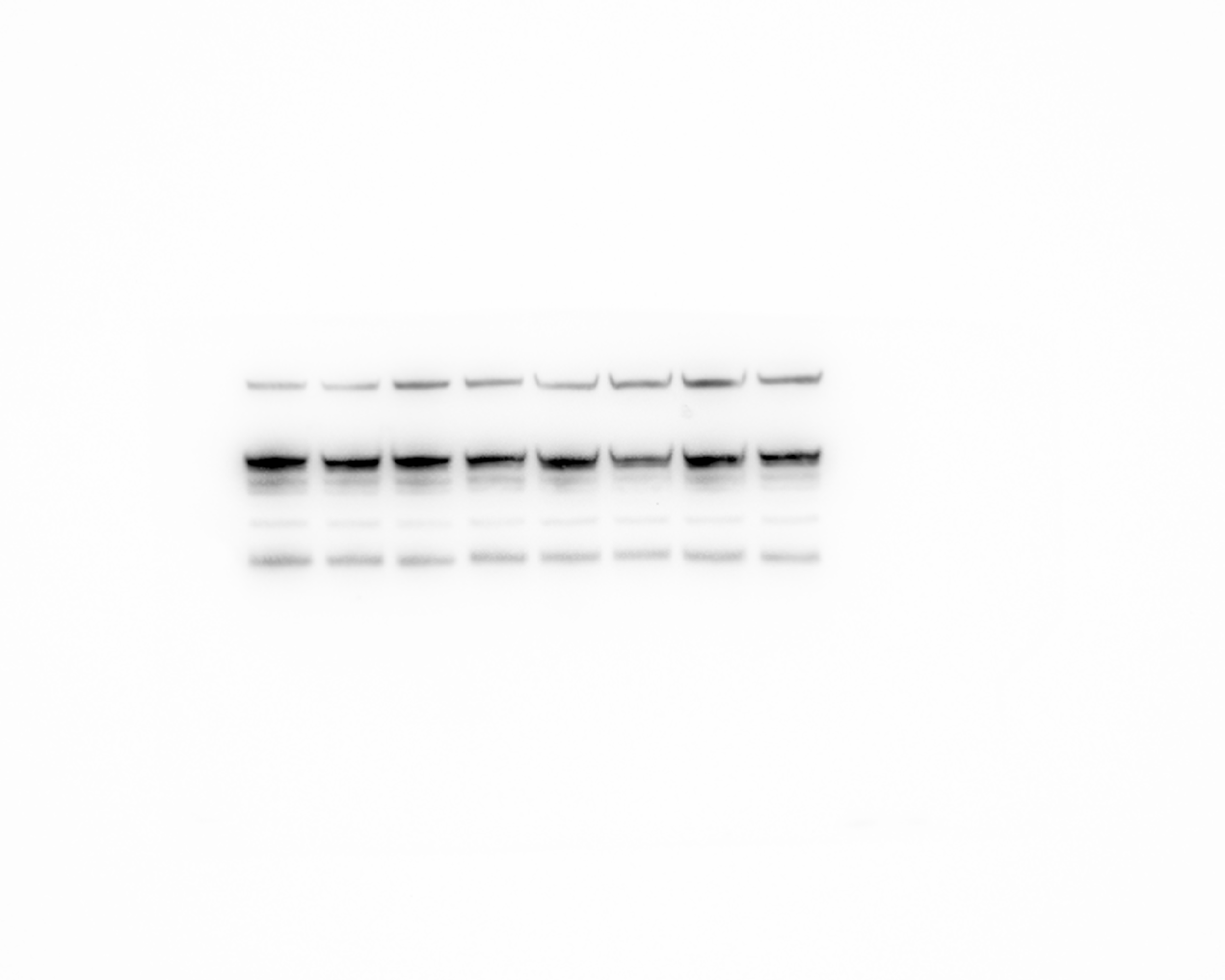

Supplement: Figure 4—figure supplement 1—source data 2. [file elife-98631-fig4-figsupp1-data2.zip › SFig4A PBC2B.tif]

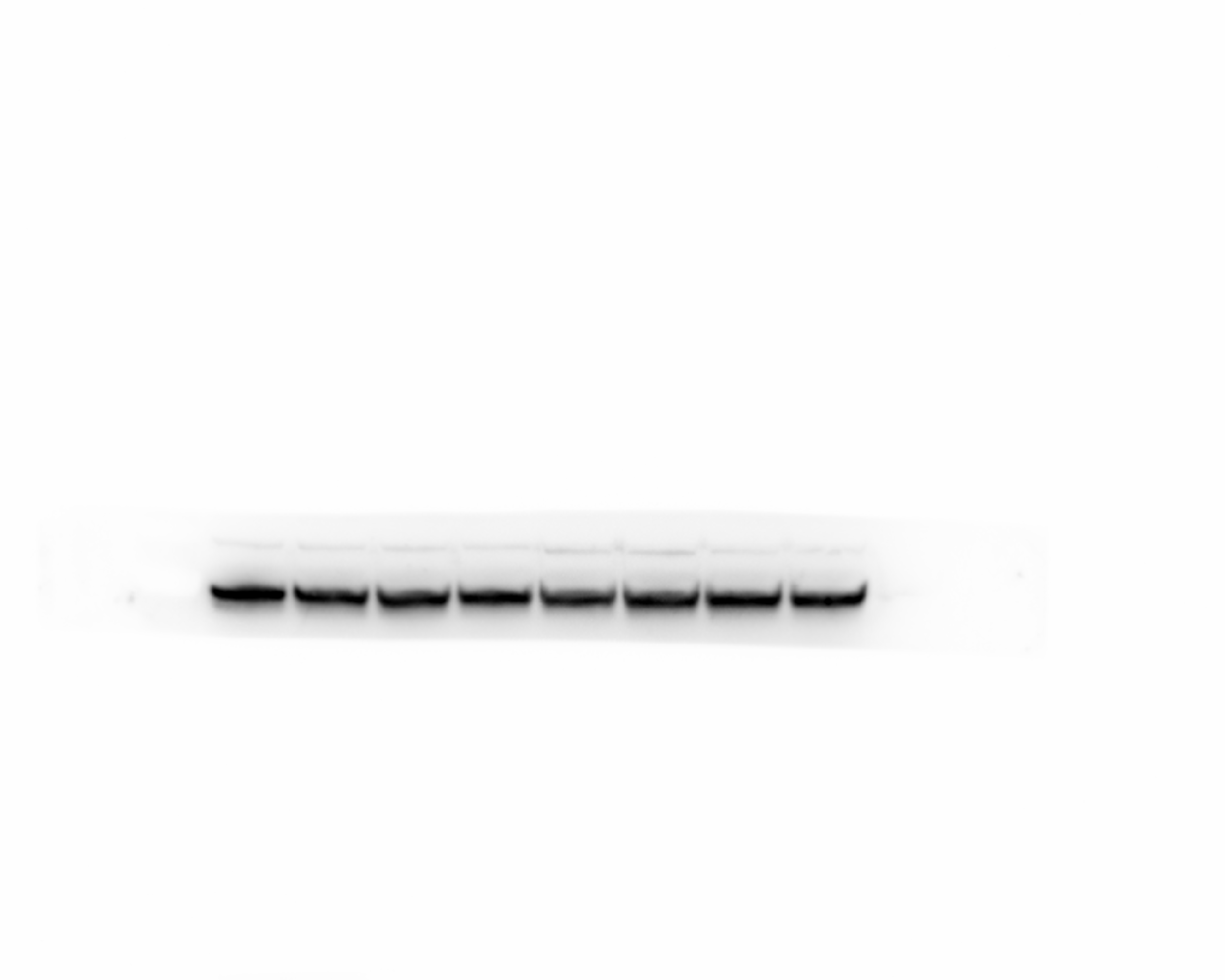

Supplement: Figure 4—figure supplement 1—source data 2. [file elife-98631-fig4-figsupp1-data2.zip › SFig4A FUS.tif]

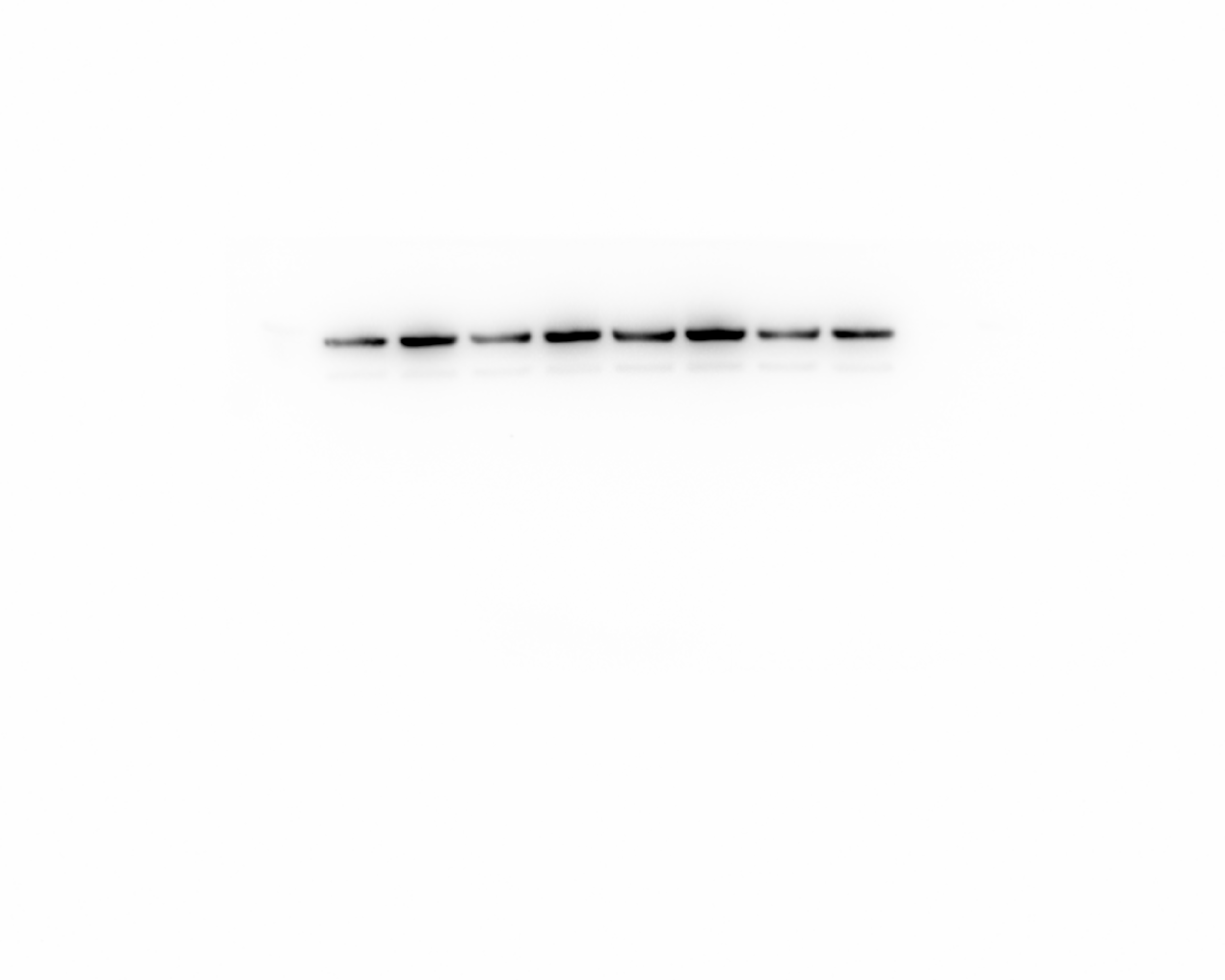

Supplement: Figure 4—figure supplement 1—source data 2. [file elife-98631-fig4-figsupp1-data2.zip › SFig4A EIF3J.tif]

Images corresponding to **Figure 5**:

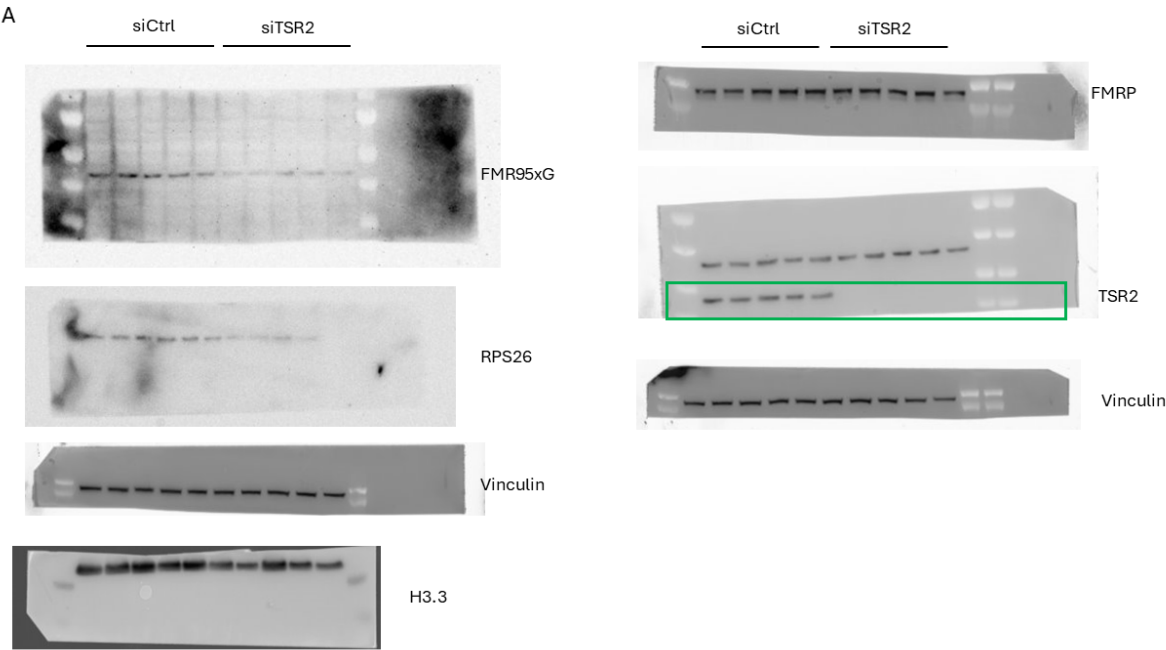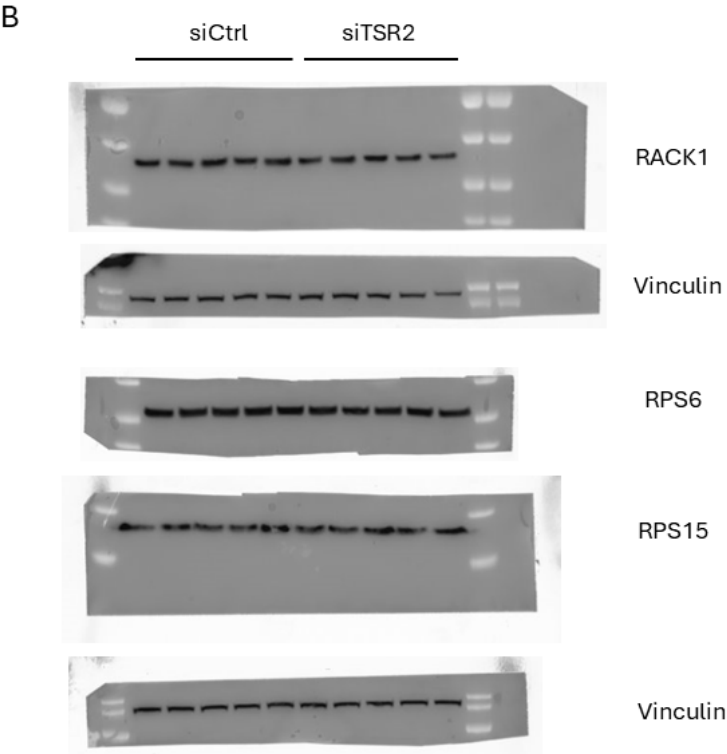

C

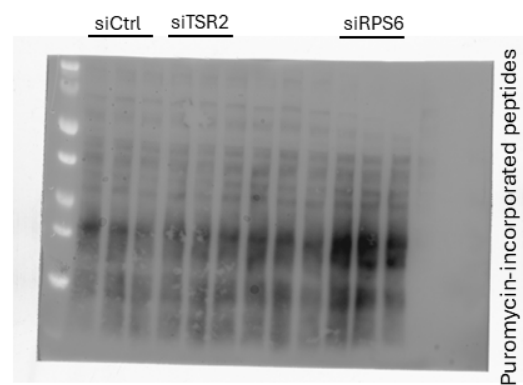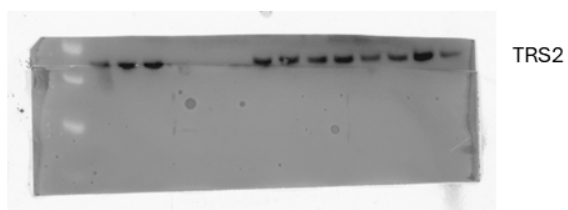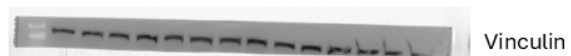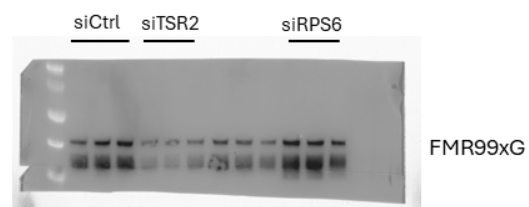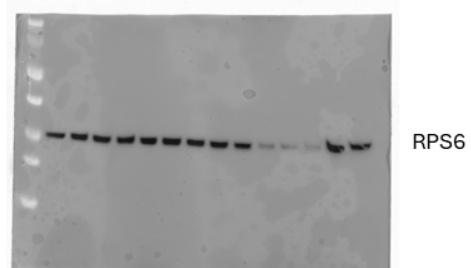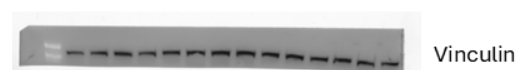

Supplement: Figure 5—source data 1. [file elife-98631-fig5-data1.zip › Figure 5 - source data 1.pdf]

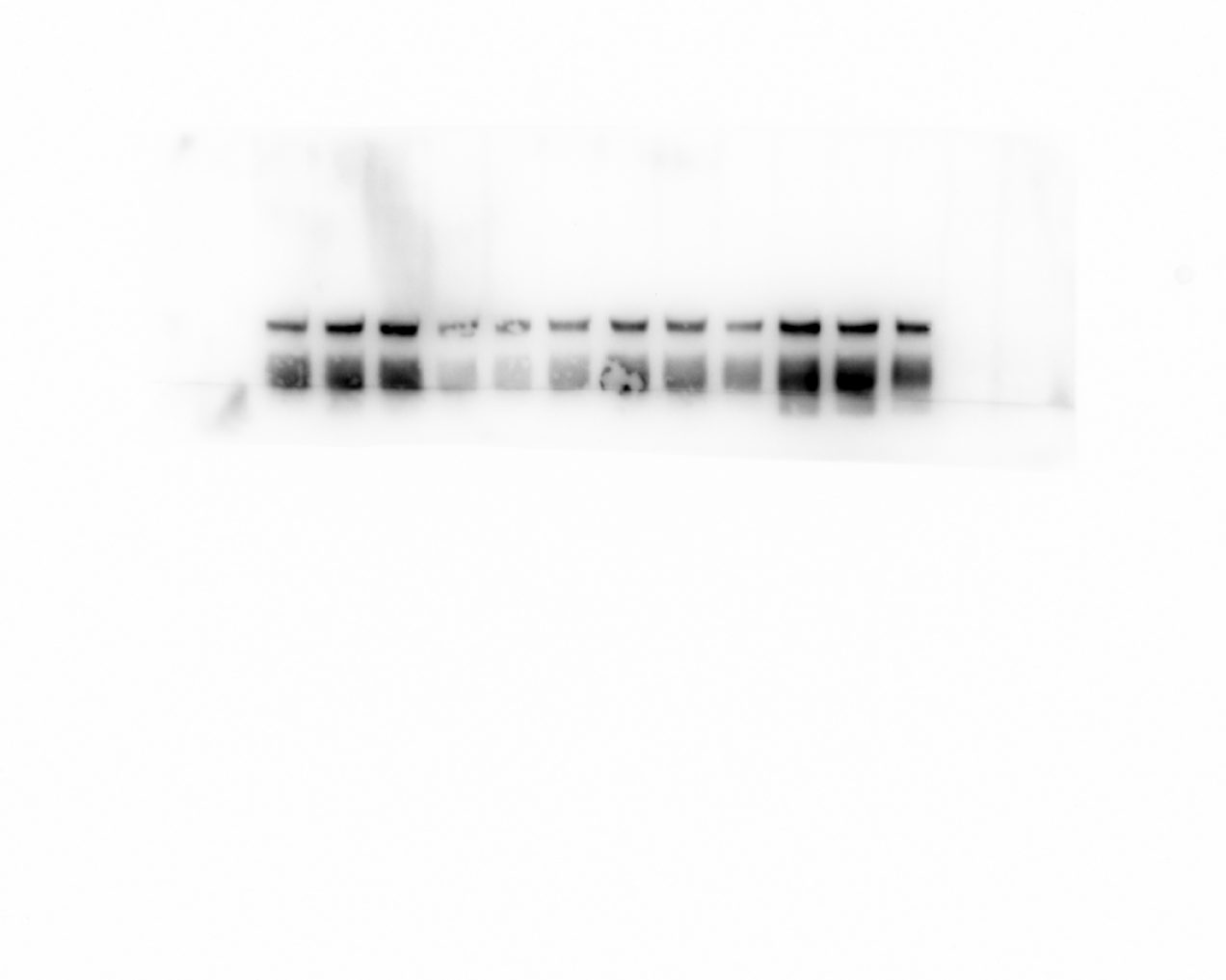

Supplement: Figure 5—source data 2. [file elife-98631-fig5-data2.zip › Figure 5/5C FMR99xG.tif]

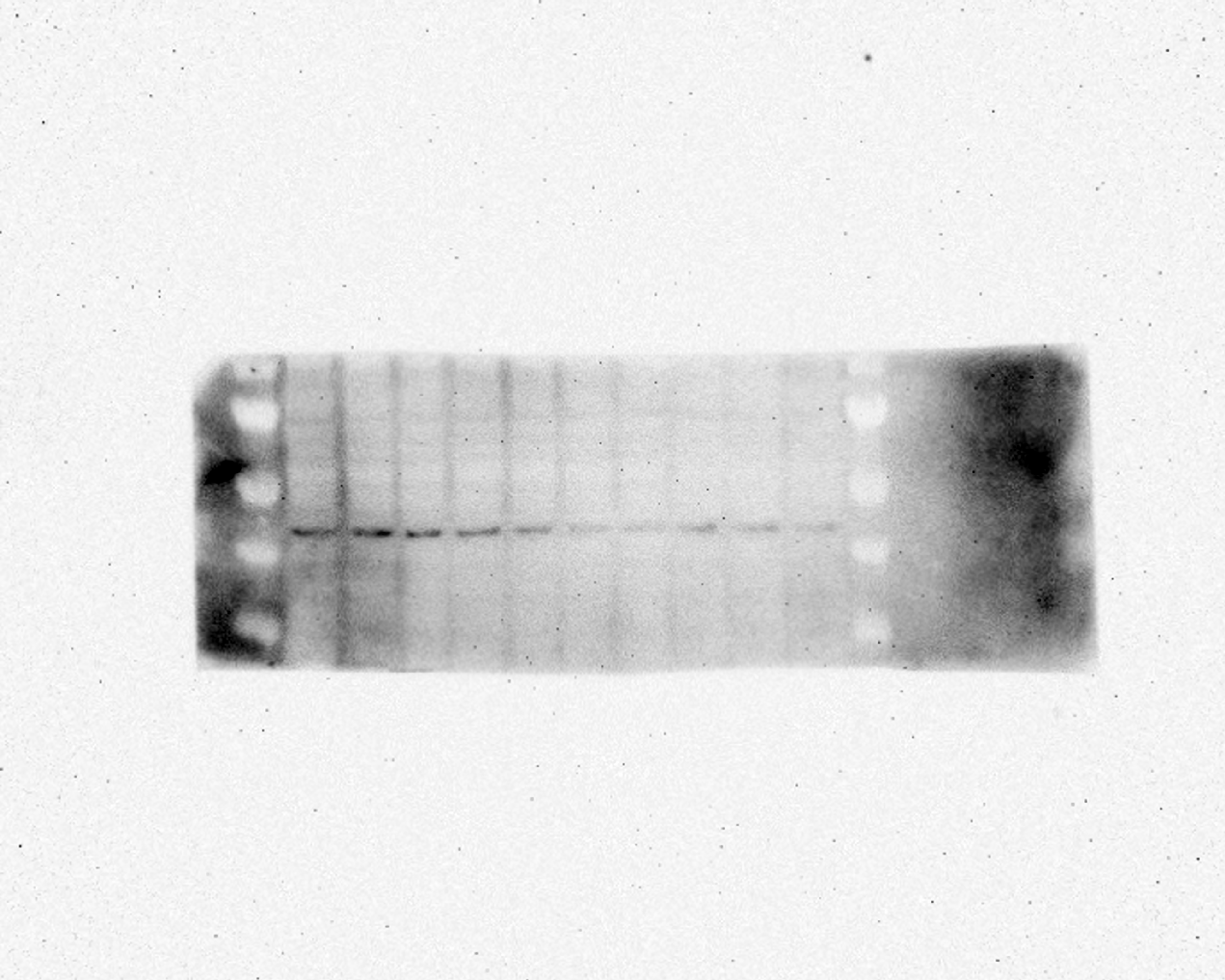

Supplement: Figure 5—source data 2. [file elife-98631-fig5-data2.zip › Figure 5/5A FMR95xG.tif]

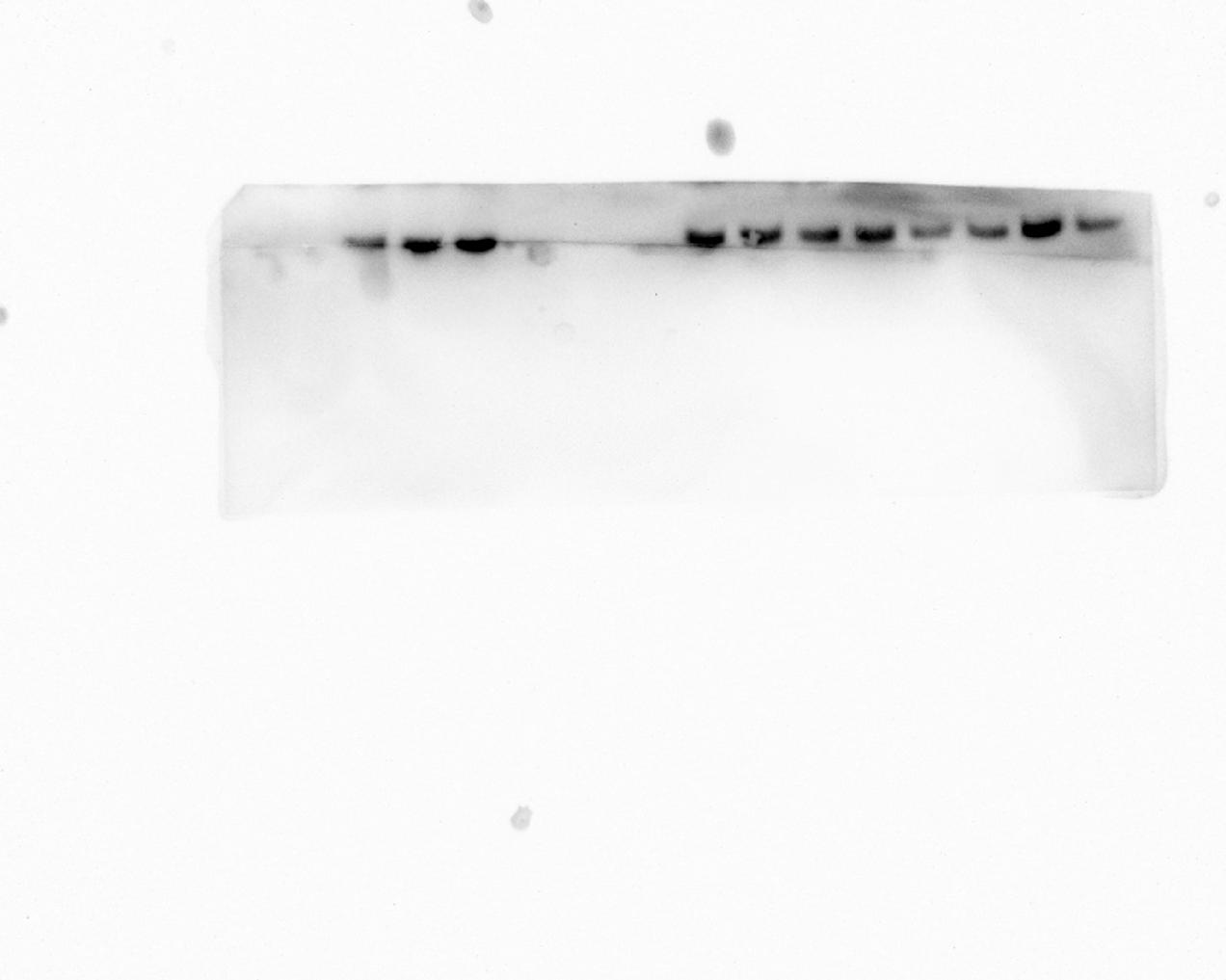

Supplement: Figure 5—source data 2. [file elife-98631-fig5-data2.zip › Figure 5/5C TSR2.tif]

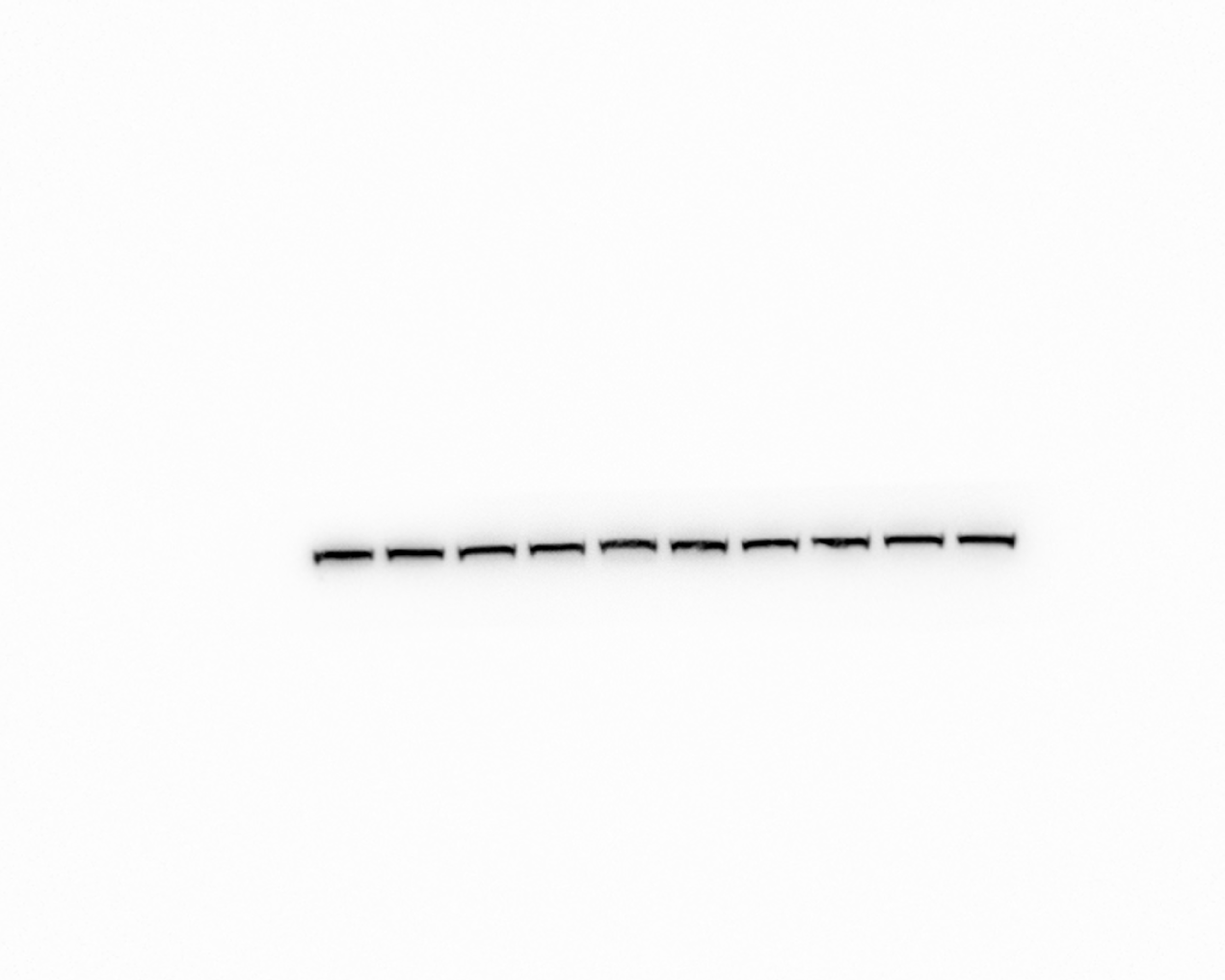

Supplement: Figure 5—source data 2. [file elife-98631-fig5-data2.zip › Figure 5/5B Vinculin to RPS6 and RPS15 and H3.3 from 5A.tif]

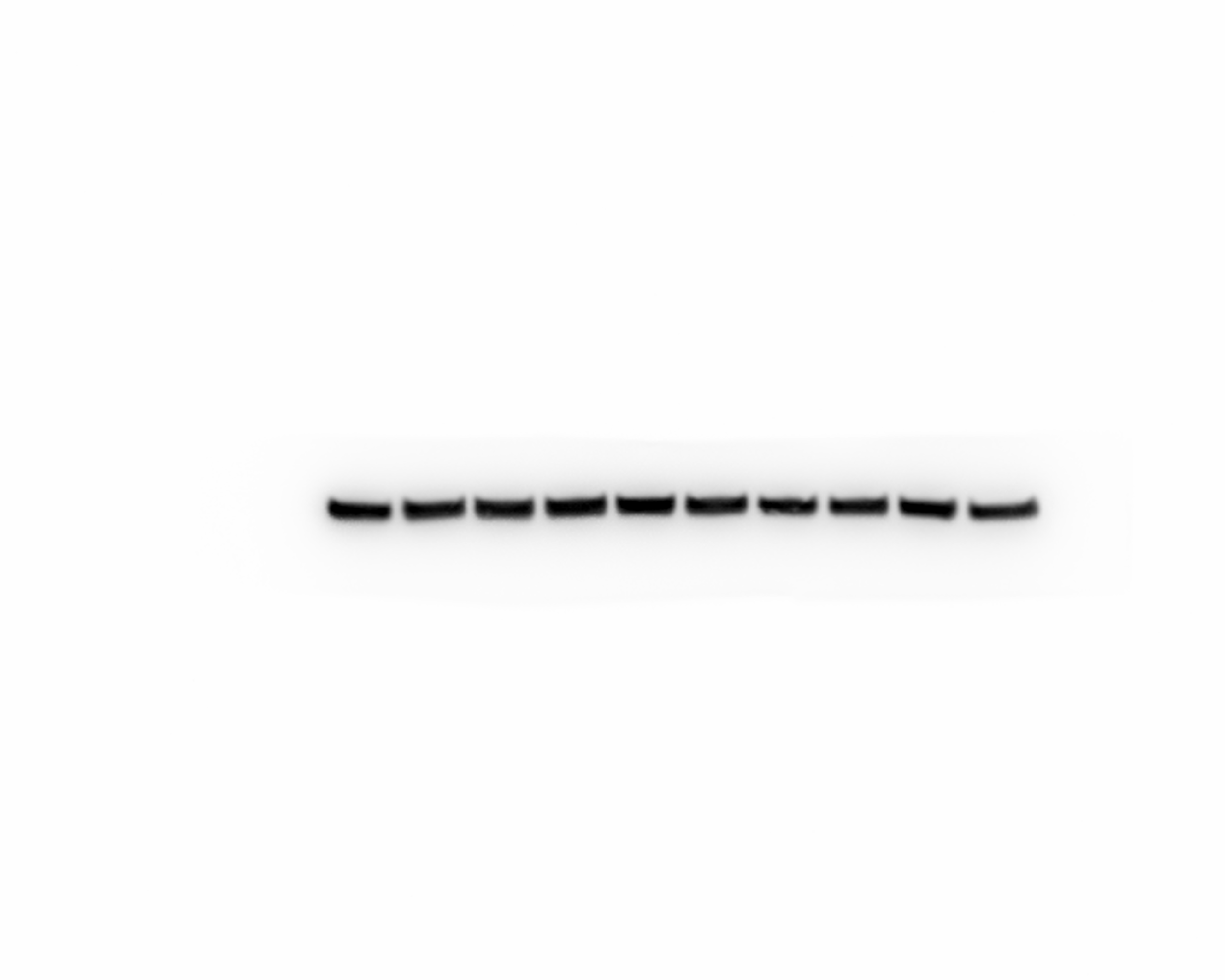

Supplement: Figure 5—source data 2. [file elife-98631-fig5-data2.zip › Figure 5/5B RPS6.tif]

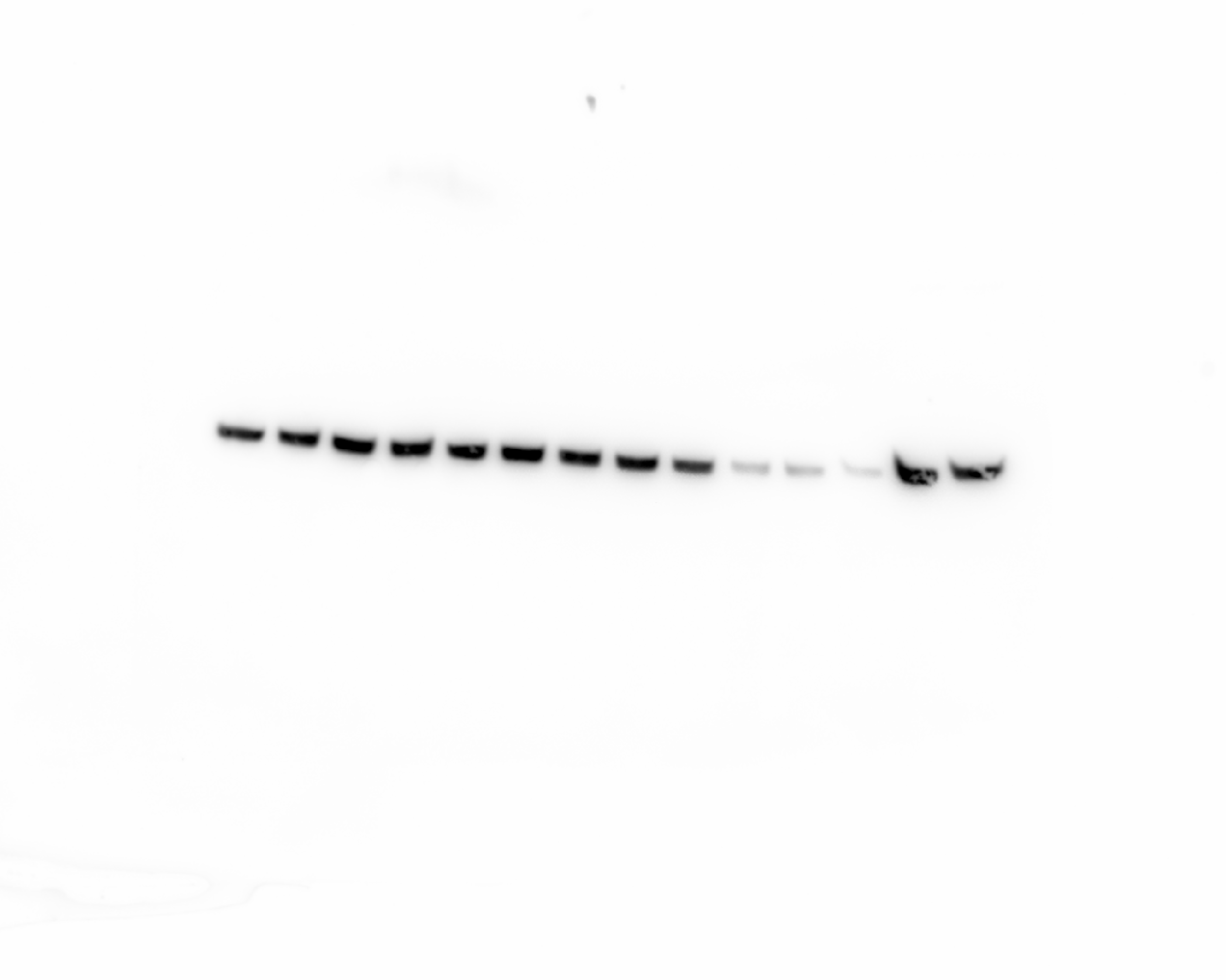

Supplement: Figure 5—source data 2. [file elife-98631-fig5-data2.zip › Figure 5/5C RPS6.tif]

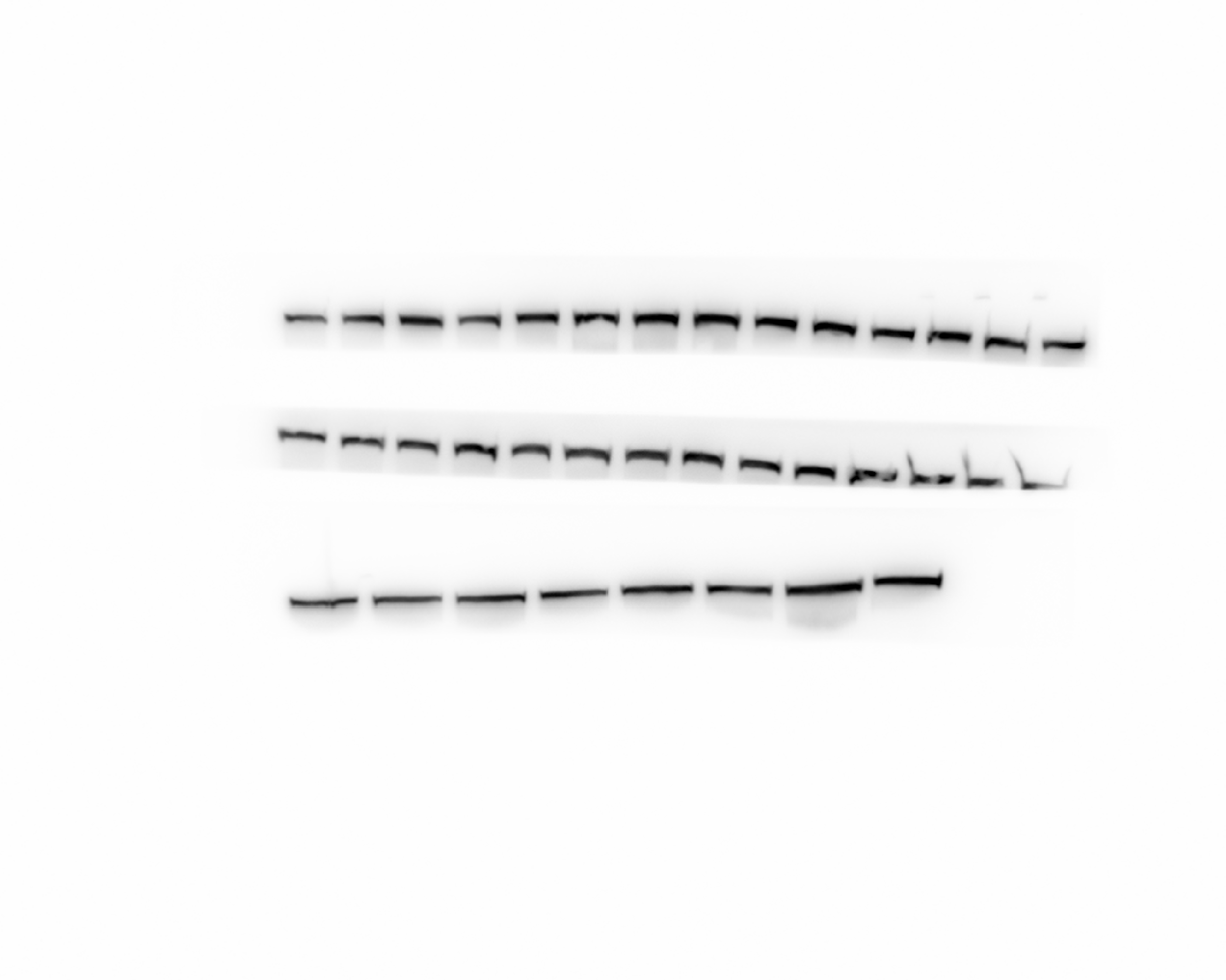

Supplement: Figure 5—source data 2. [file elife-98631-fig5-data2.zip › Figure 5/5C Vinuclin.tif]

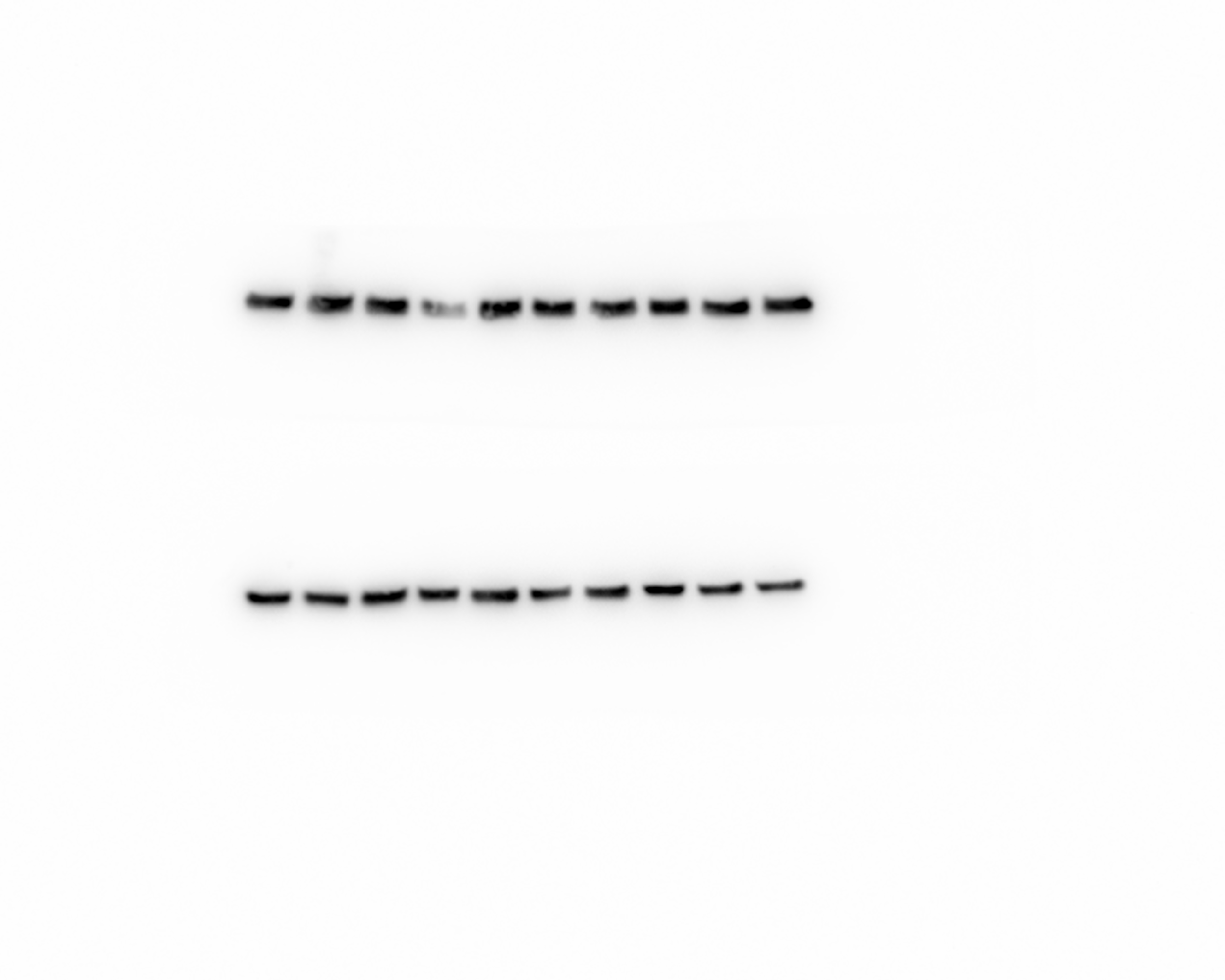

Supplement: Figure 5—source data 2. [file elife-98631-fig5-data2.zip › Figure 5/5B RACK1.tif]

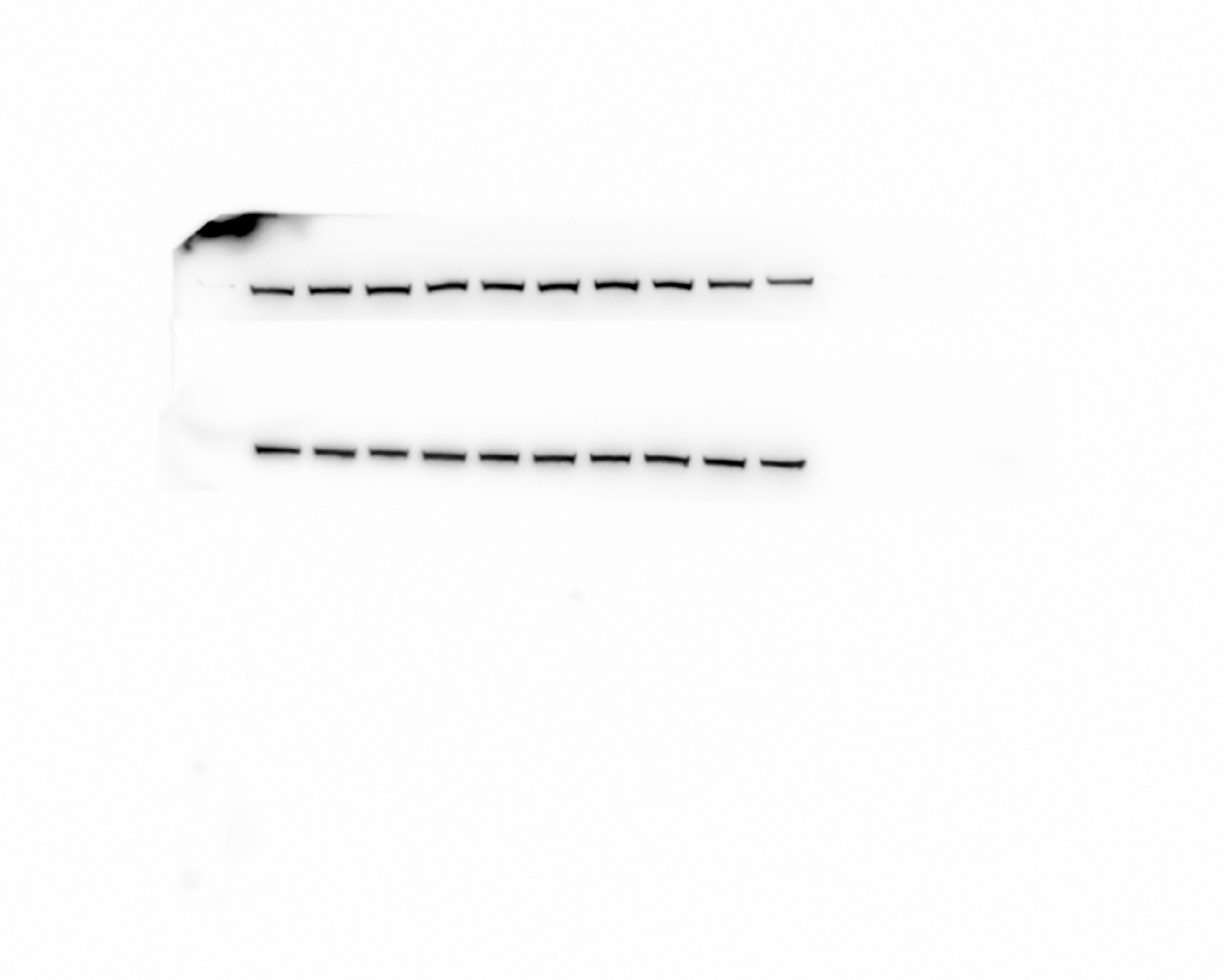

Supplement: Figure 5—source data 2. [file elife-98631-fig5-data2.zip › Figure 5/5A Vinuclin and 5B Vinculin to RACK1.tif]

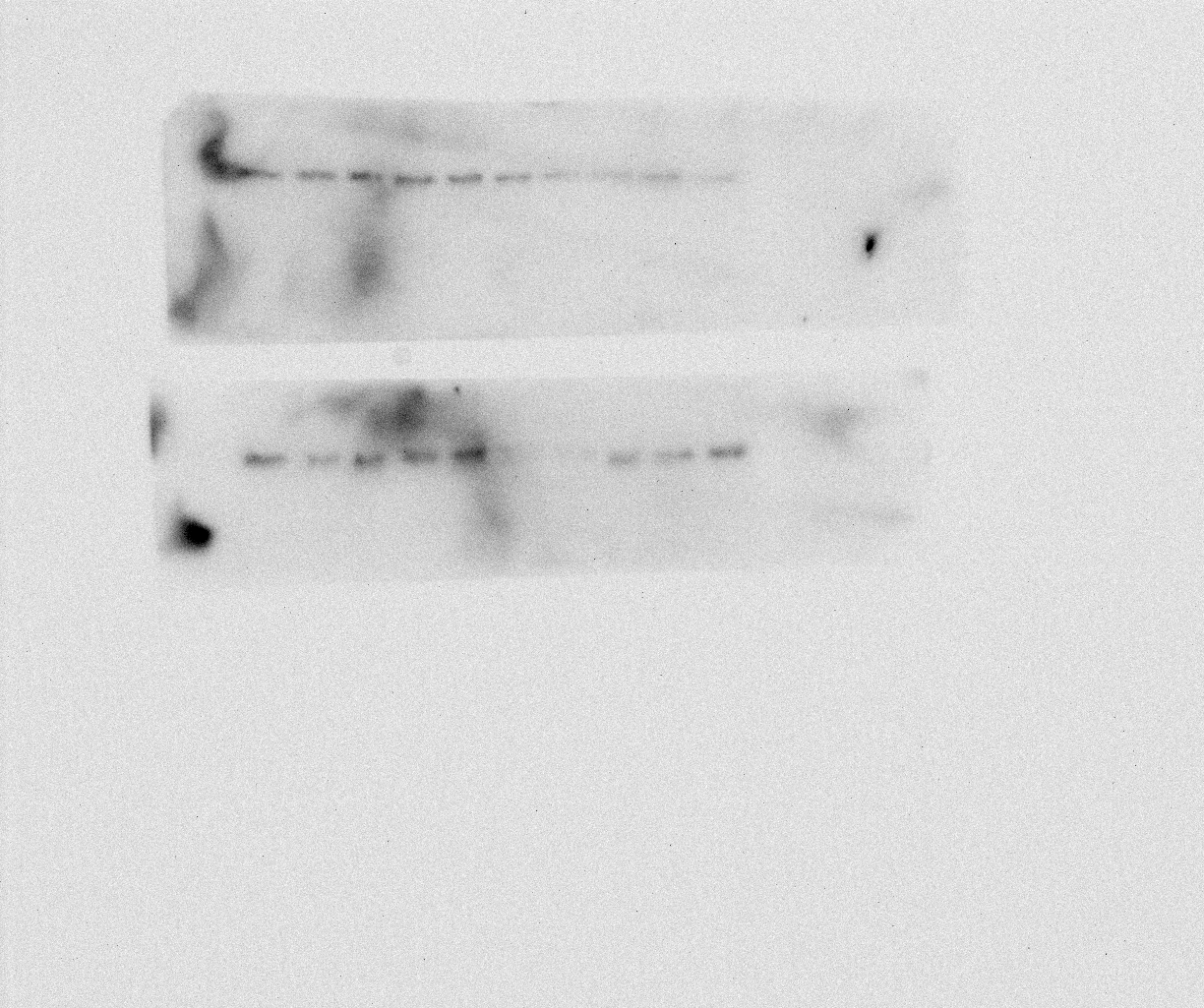

Supplement: Figure 5—source data 2. [file elife-98631-fig5-data2.zip › Figure 5/5A RPS26.tif]

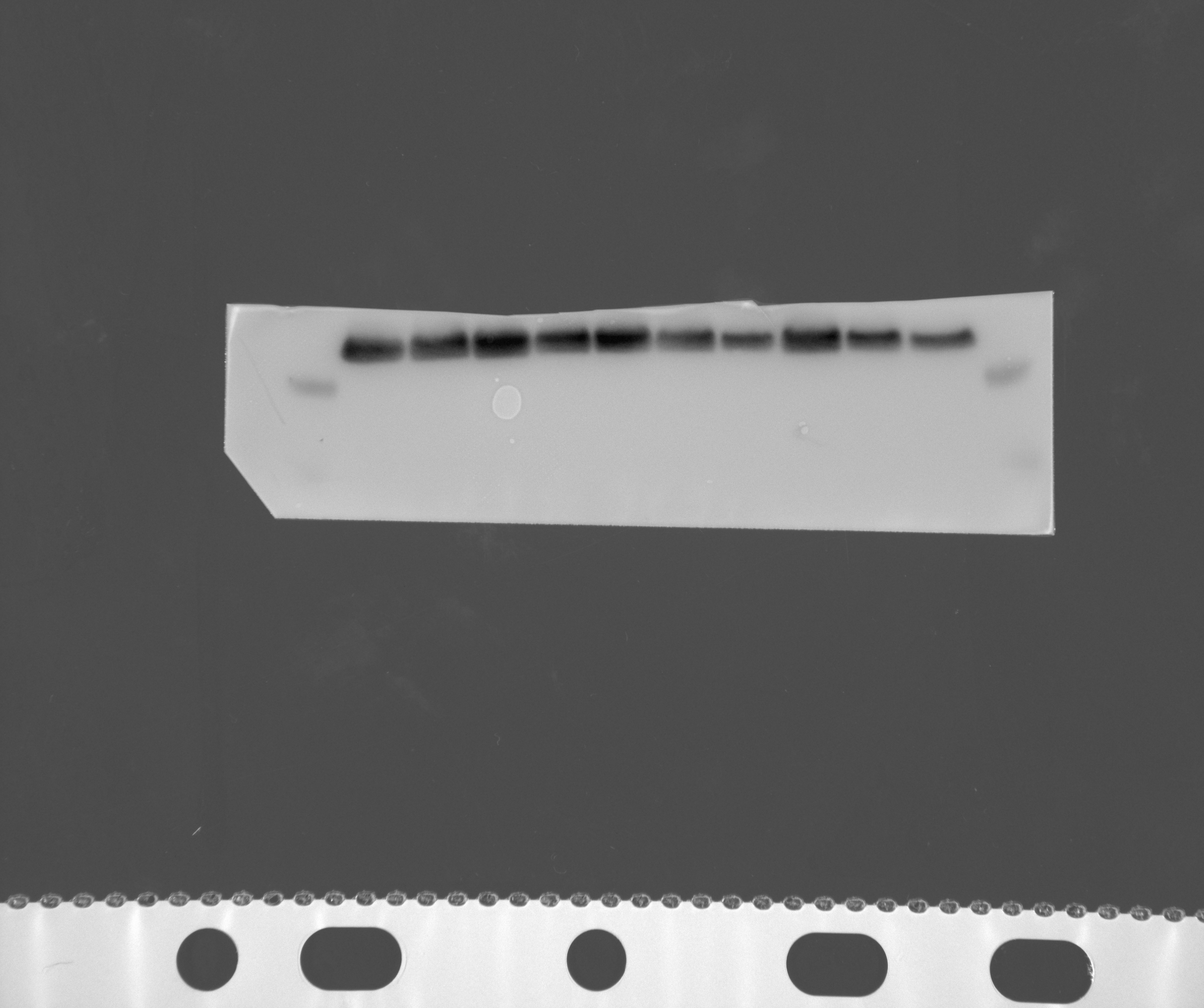

Supplement: Figure 5—source data 2. [file elife-98631-fig5-data2.zip › Figure 5/5A H3.3.tif]

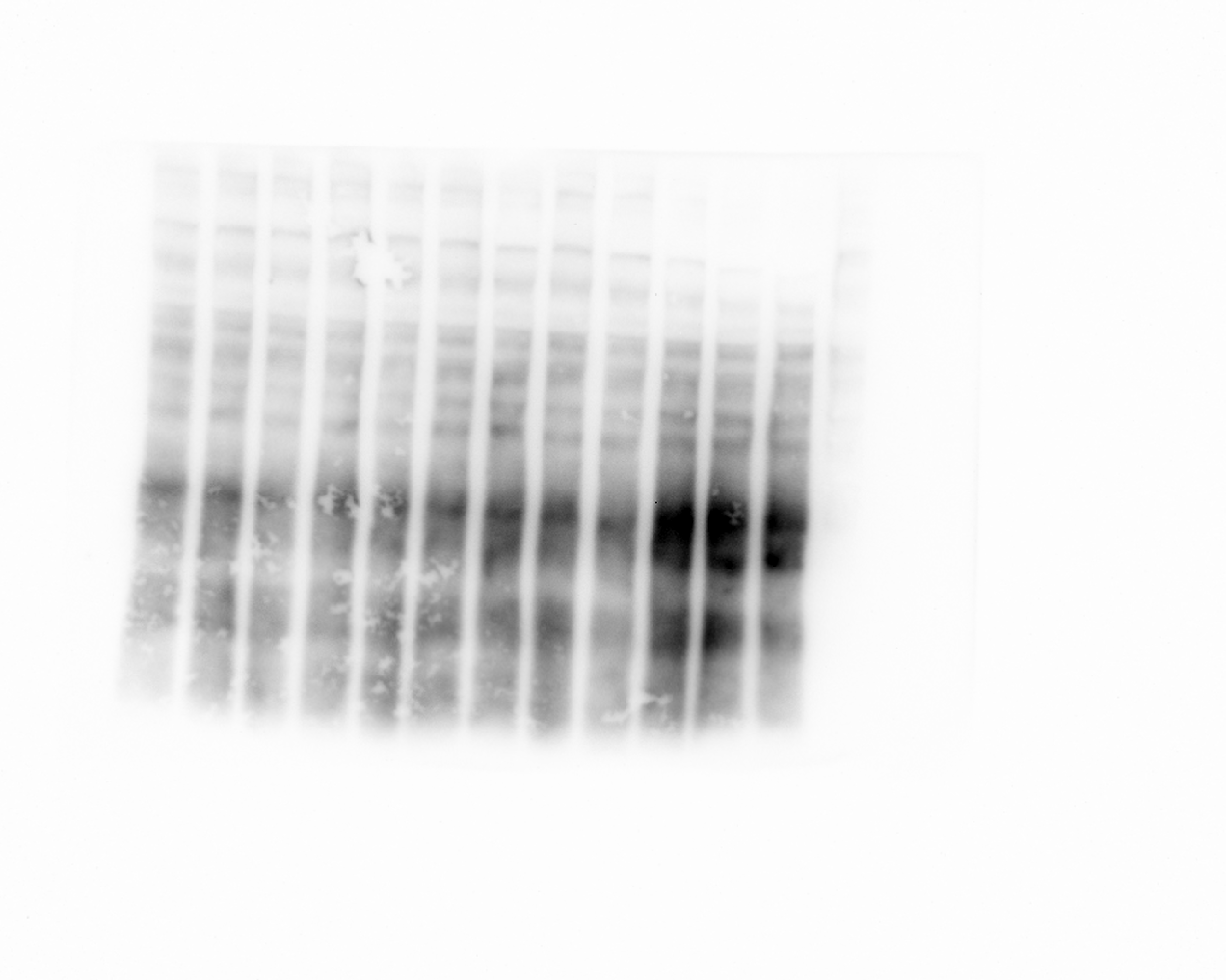

Supplement: Figure 5—source data 2. [file elife-98631-fig5-data2.zip › Figure 5/5C PURO.tif]

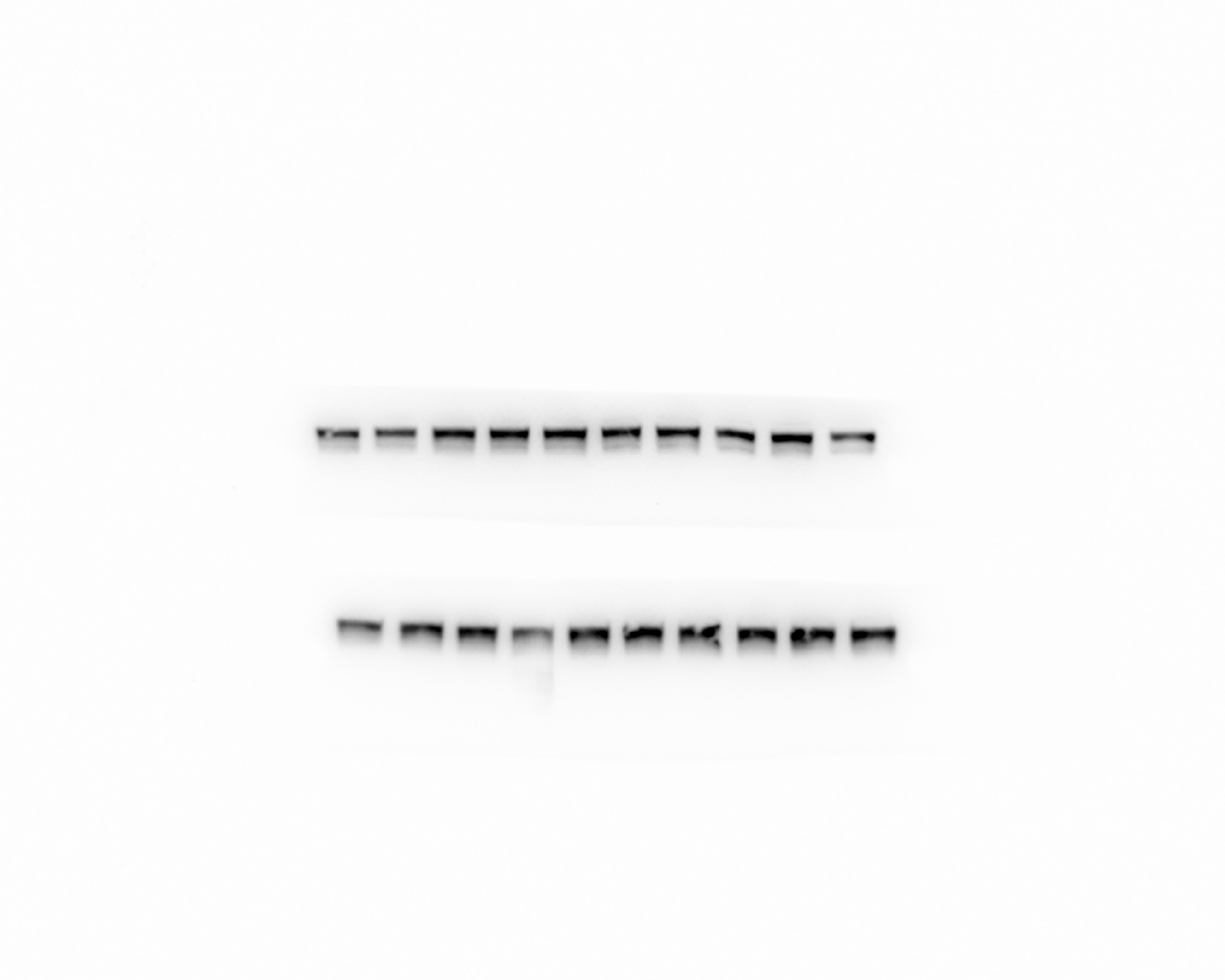

Supplement: Figure 5—source data 2. [file elife-98631-fig5-data2.zip › Figure 5/5A FMRP.tif]

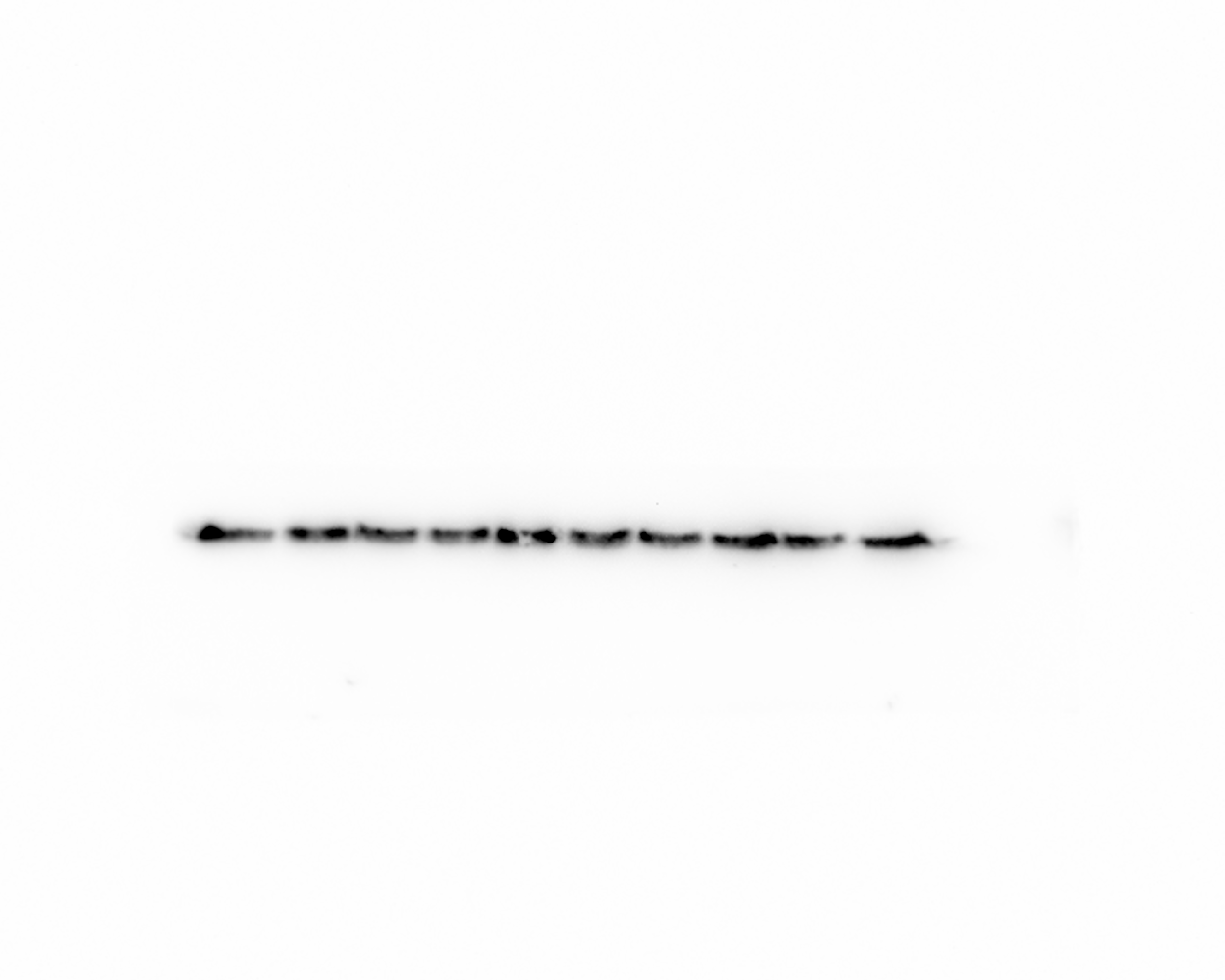

Supplement: Figure 5—source data 2. [file elife-98631-fig5-data2.zip › Figure 5/5B RPS15.tif]

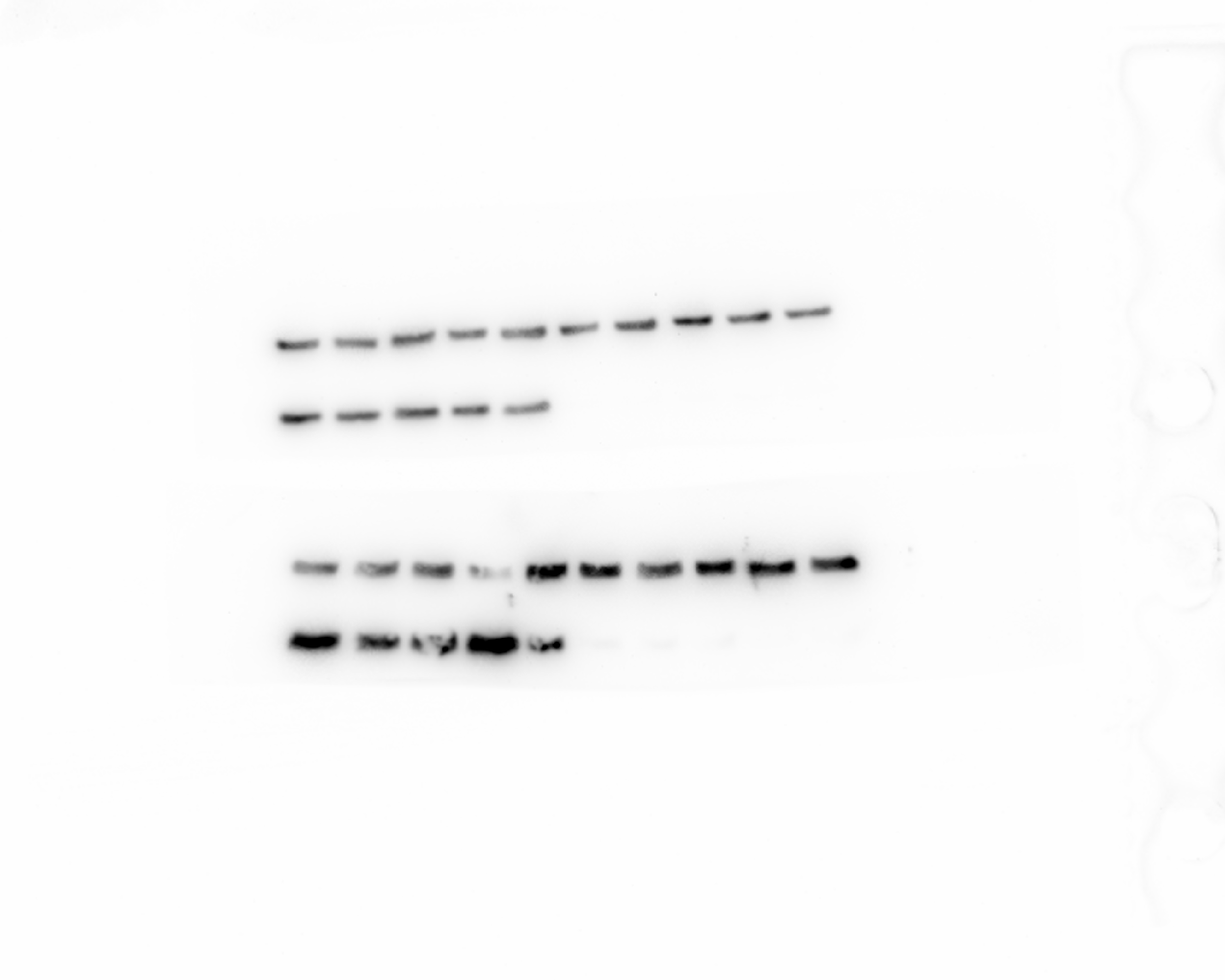

Supplement: Figure 5—source data 2. [file elife-98631-fig5-data2.zip › Figure 5/5A TSR2.tif]

Images corresponding to **Figure 5 – figure supplement 1:**

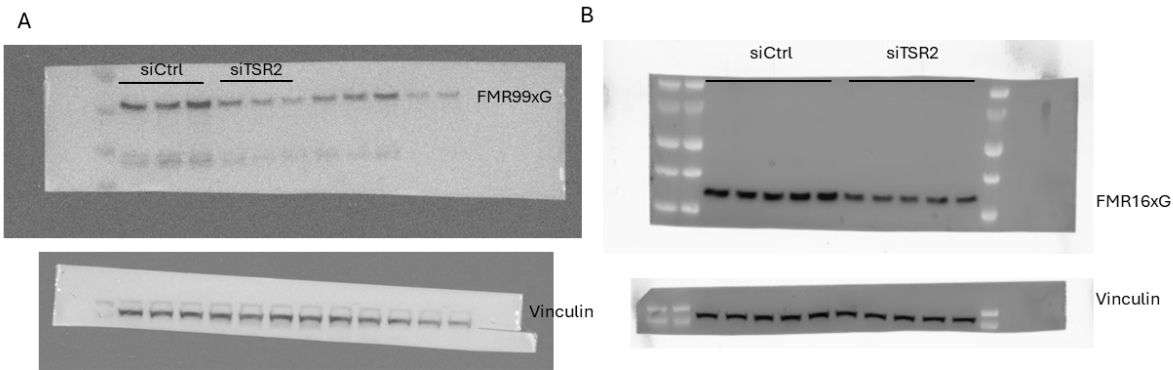

Supplement: Figure 5—figure supplement 1—source data 1. [file elife-98631-fig5-figsupp1-data1.zip › Figure 5 - figure supplement 1 - source data 1.pdf]

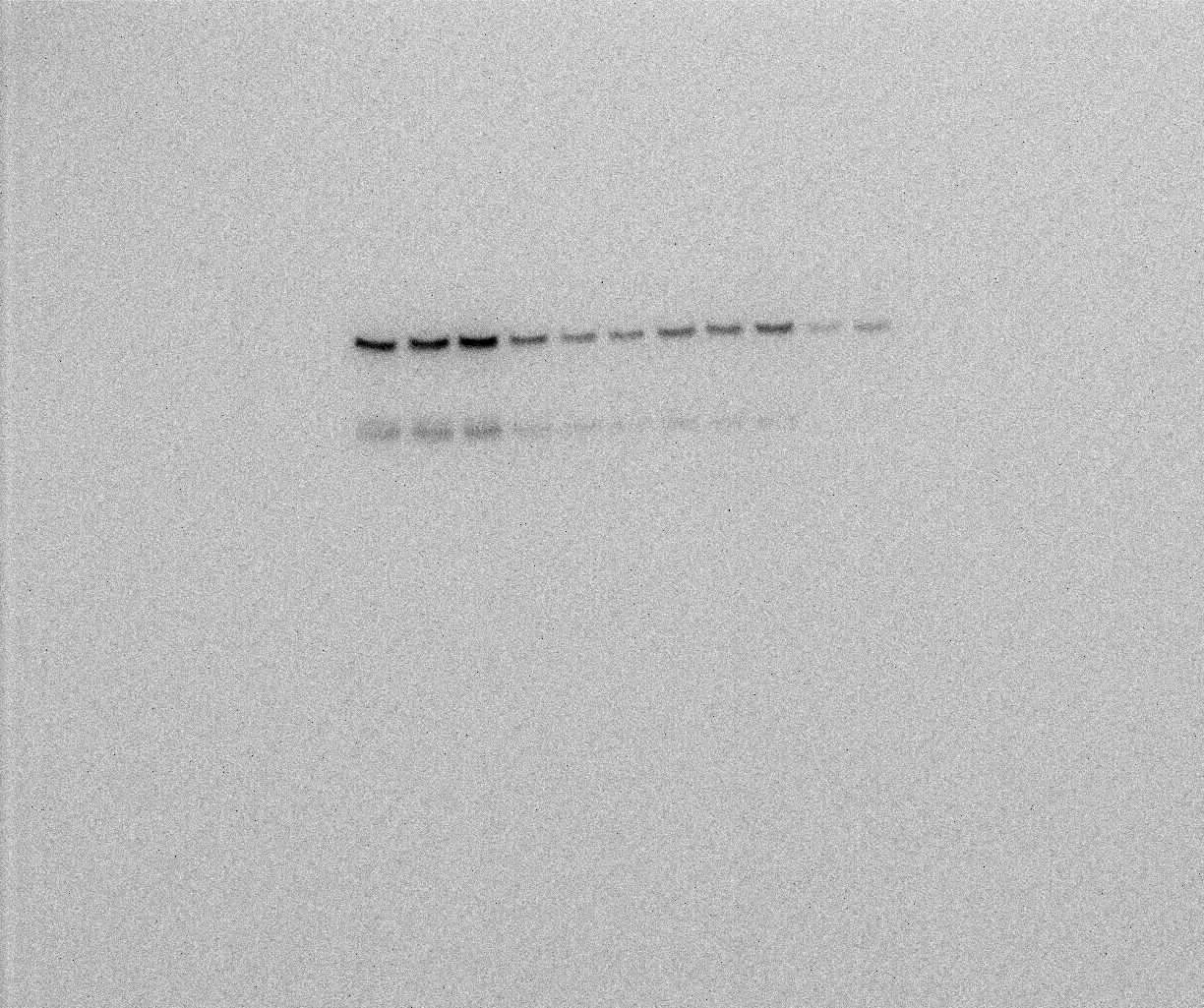

Supplement: Figure 5—figure supplement 1—source data 2. [file elife-98631-fig5-figsupp1-data2.zip › SFig5A FMR99xG.tif]

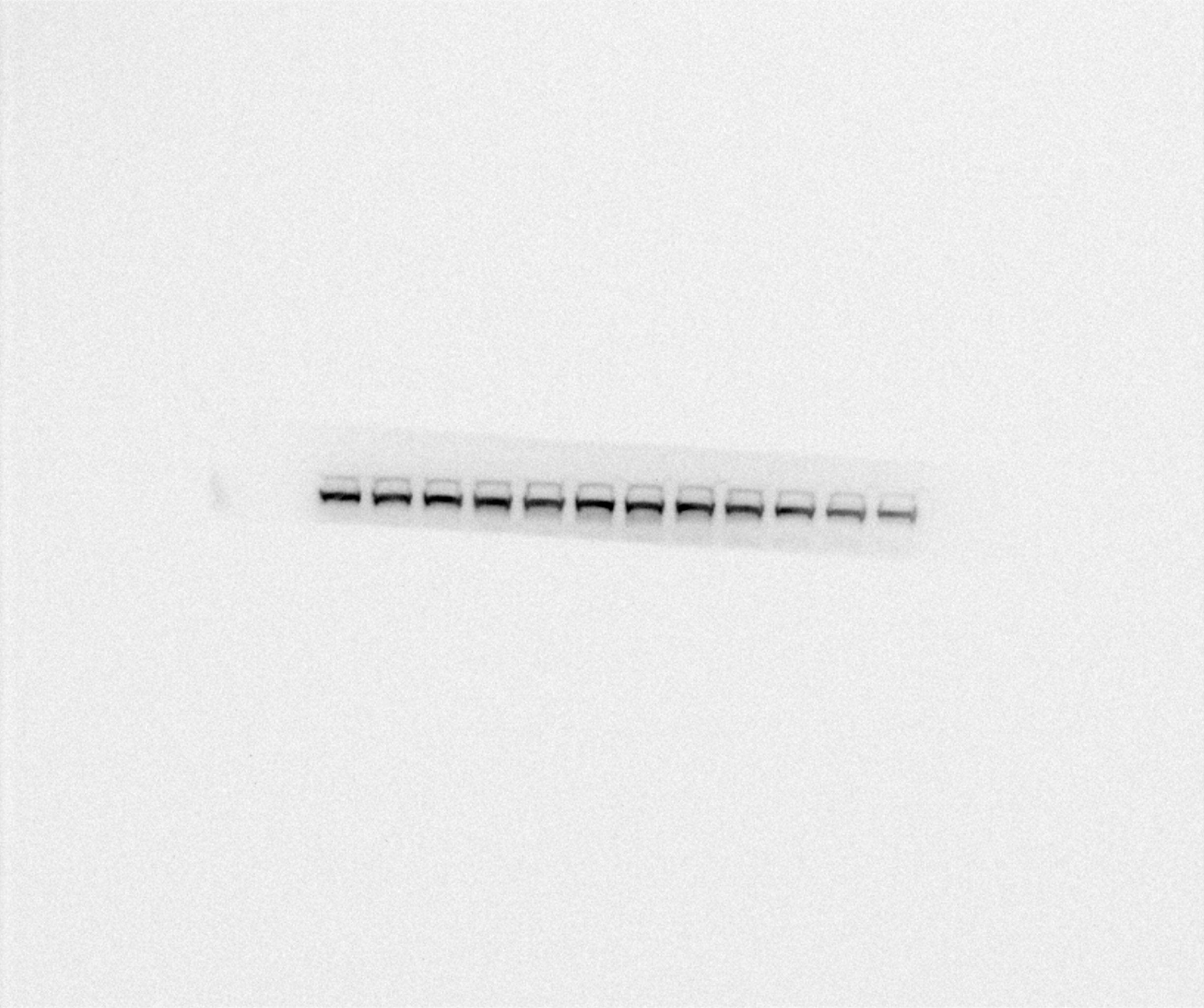

Supplement: Figure 5—figure supplement 1—source data 2. [file elife-98631-fig5-figsupp1-data2.zip › SFig5A Vinculin.tif]

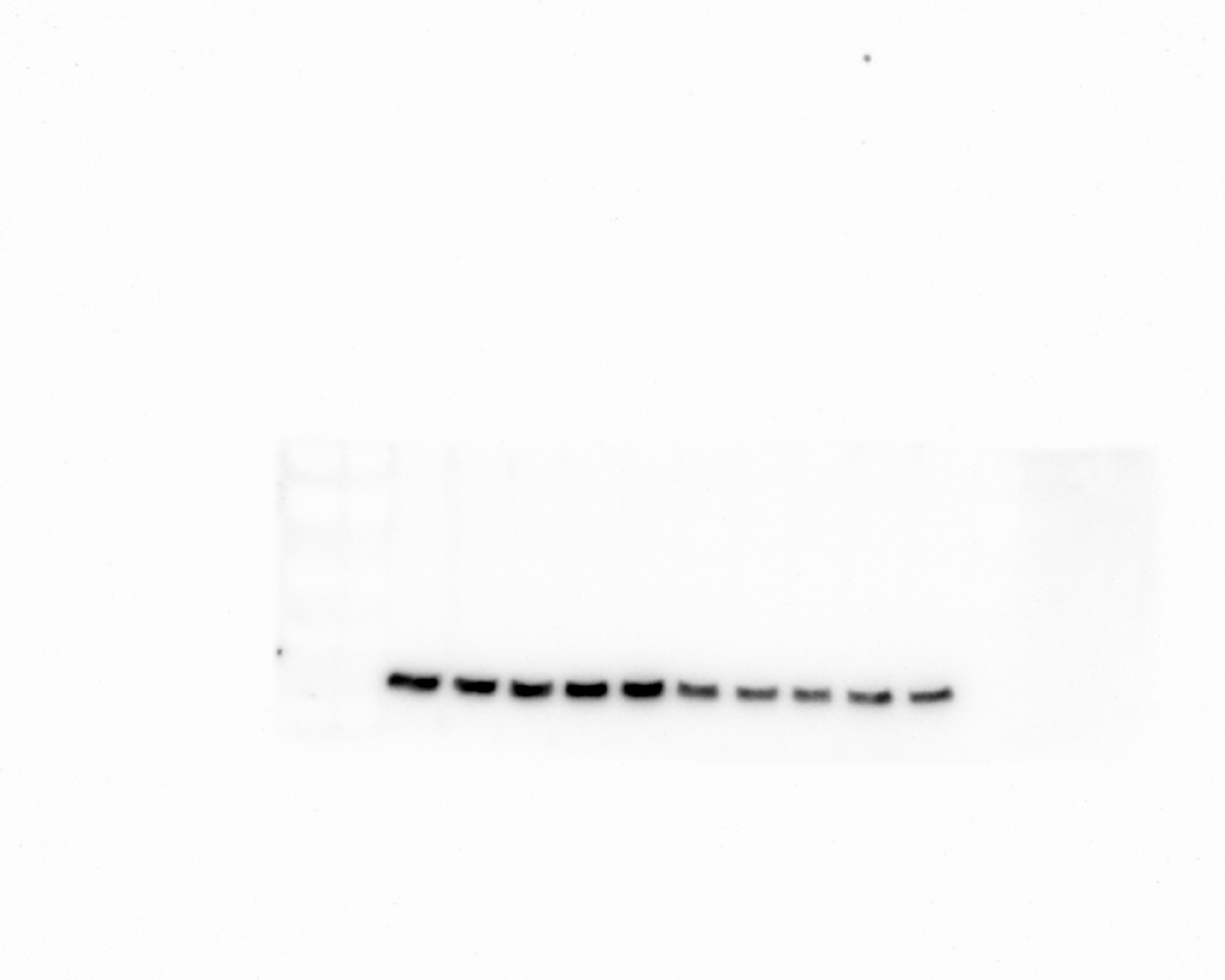

Supplement: Figure 5—figure supplement 1—source data 2. [file elife-98631-fig5-figsupp1-data2.zip › SFig5B FMR16xG.tif]

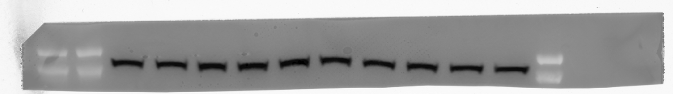

Supplement: Figure 5—figure supplement 1—source data 2. [file elife-98631-fig5-figsupp1-data2.zip › SFig5B Vinculin.tiff]

Images corresponding to **Figure 6**:

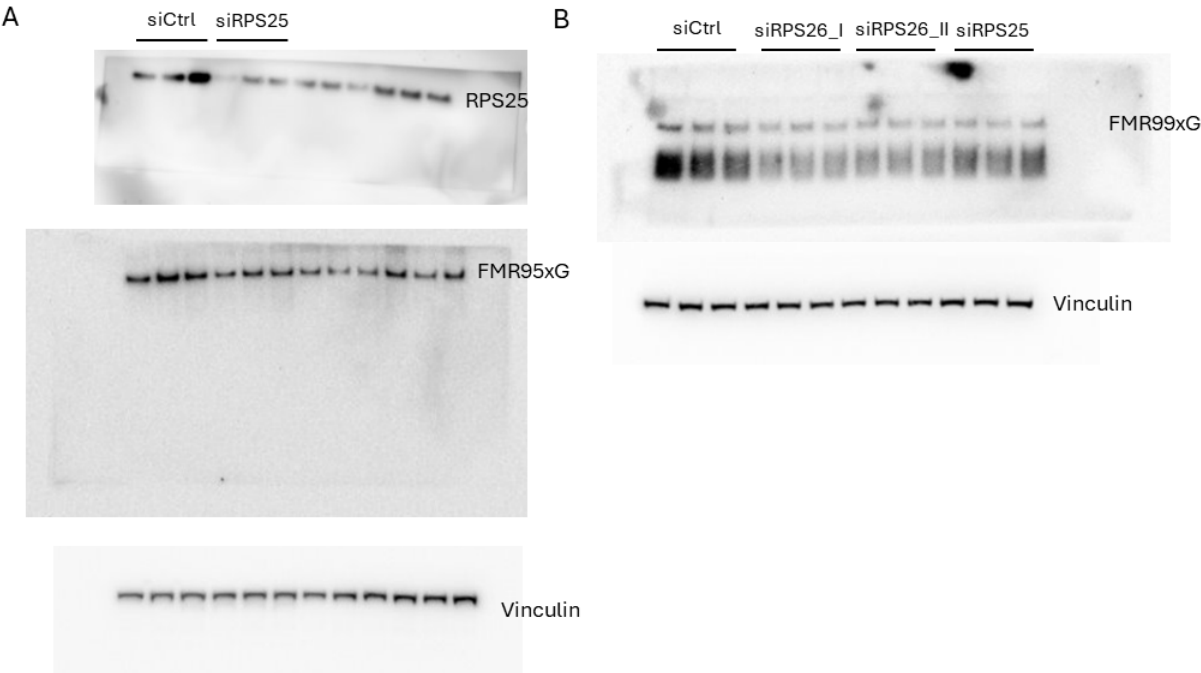

Supplement: Figure 6—source data 1. [file elife-98631-fig6-data1.zip › Figure 6 - source data 1.pdf]

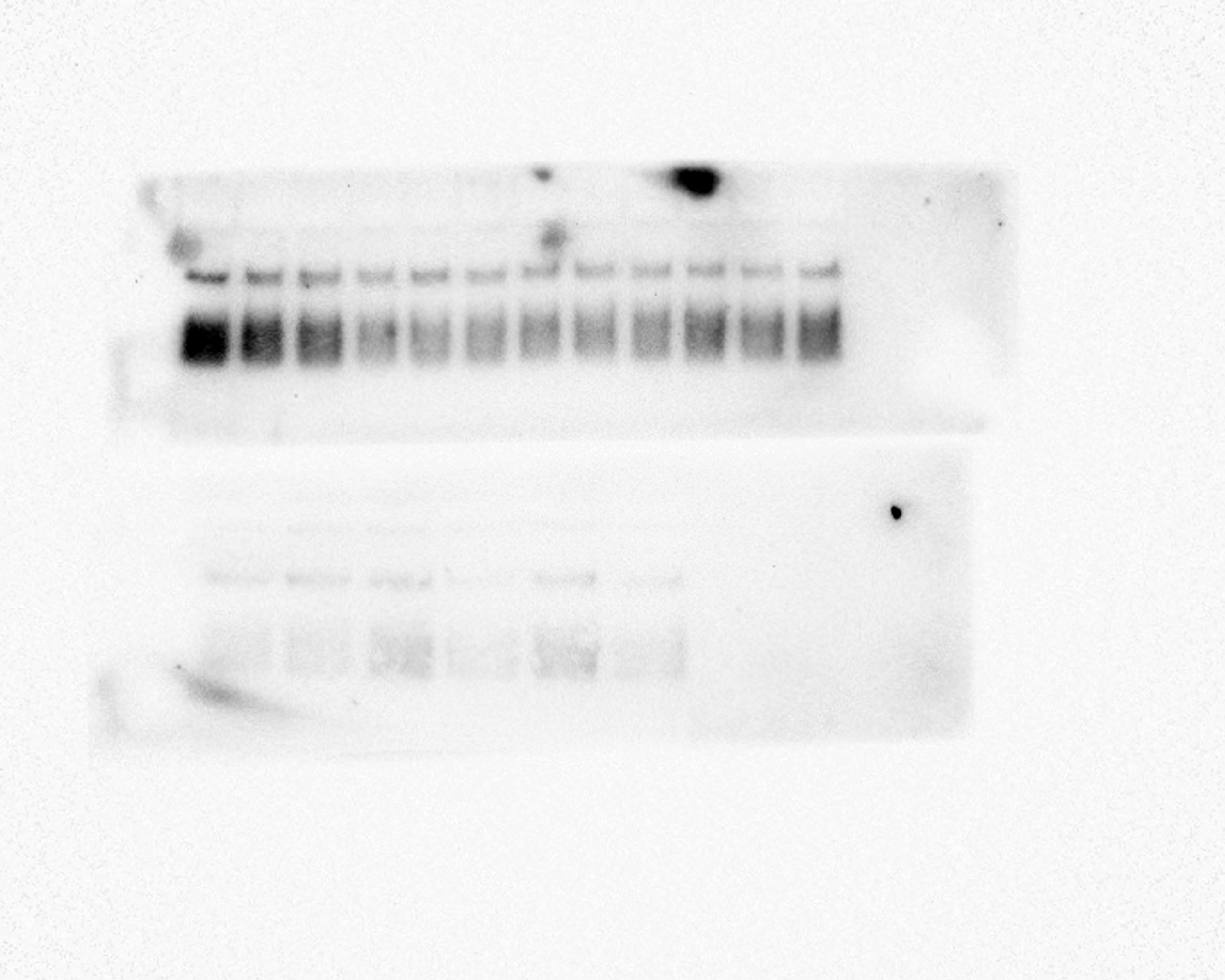

Supplement: Figure 6—source data 2. [file elife-98631-fig6-data2.zip › Figure 6 /6D FMR99xG.tif]

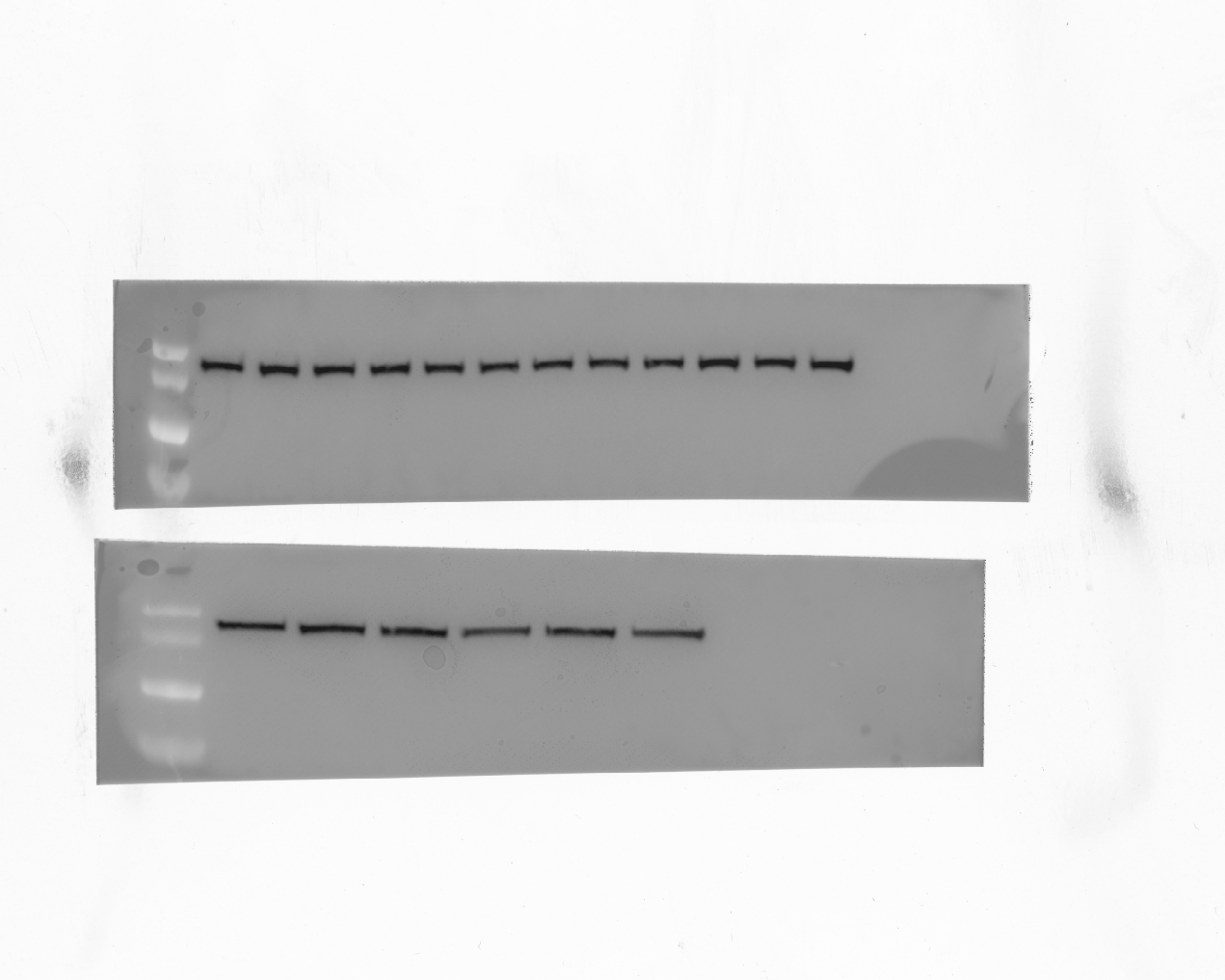

Supplement: Figure 6—source data 2. [file elife-98631-fig6-data2.zip › Figure 6 /6D Vinculin.tif]

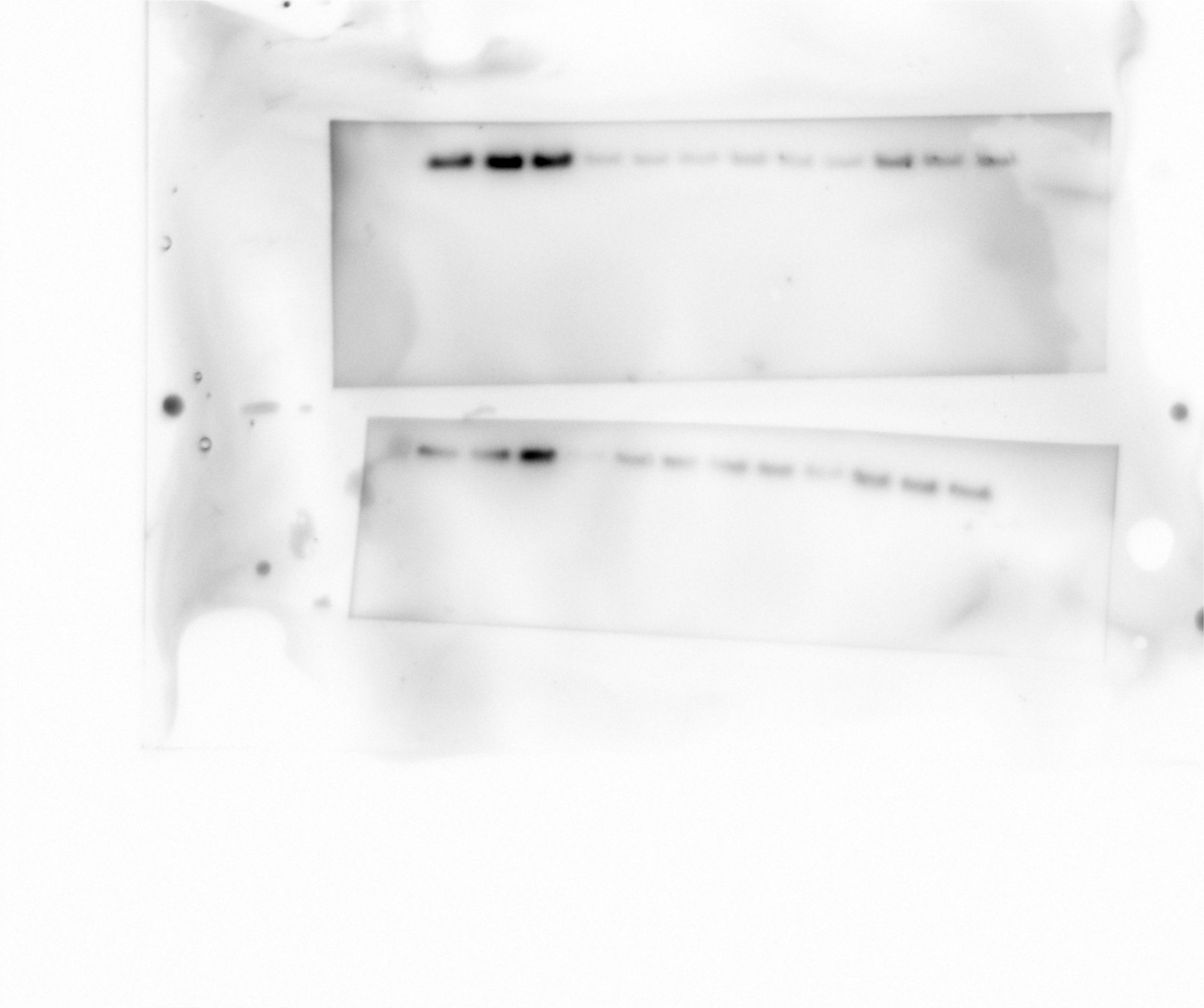

Supplement: Figure 6—source data 2. [file elife-98631-fig6-data2.zip › Figure 6 /6A RPS25.tif]

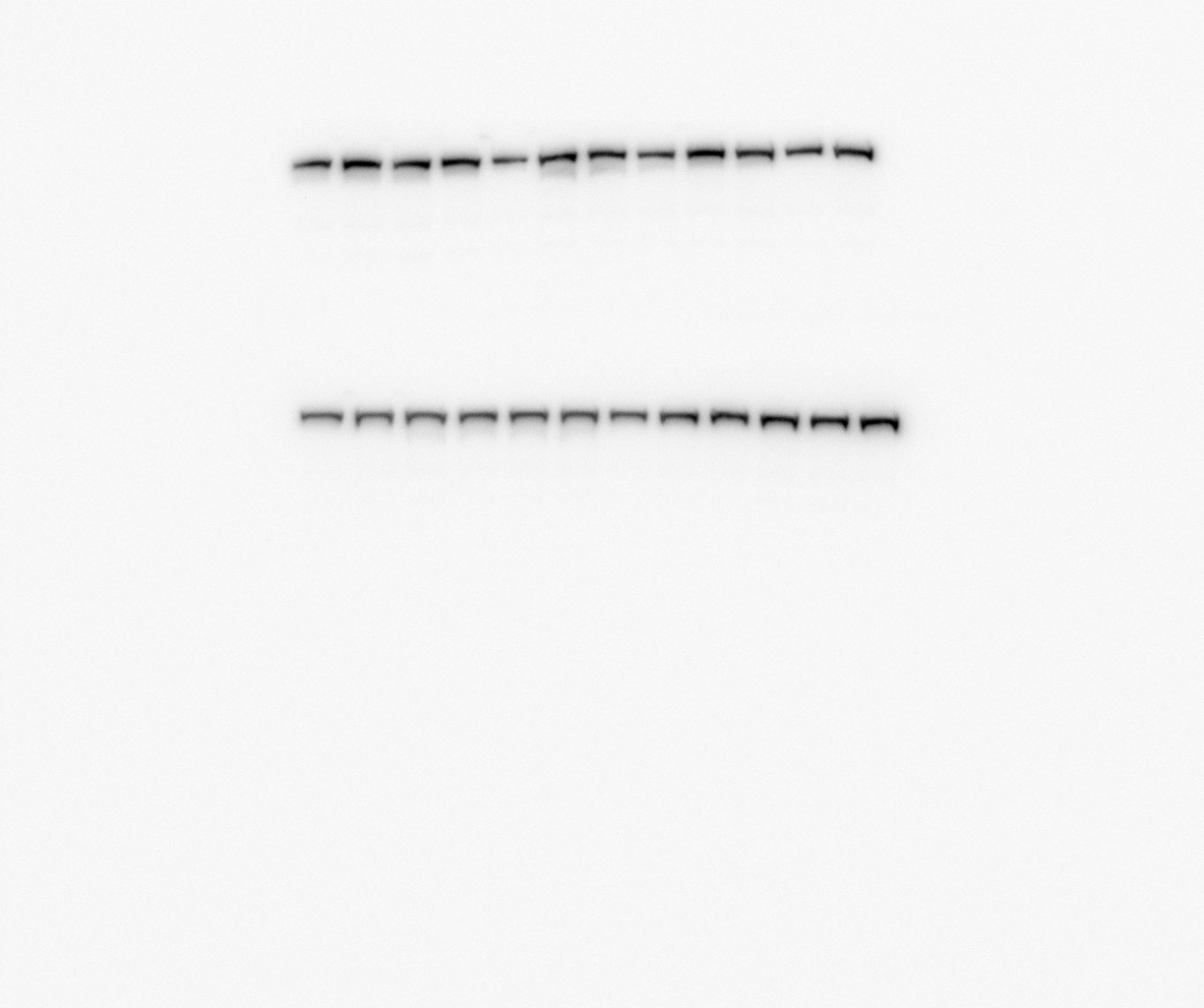

Supplement: Figure 6—source data 2. [file elife-98631-fig6-data2.zip › Figure 6 /6A Vinculin.tif]

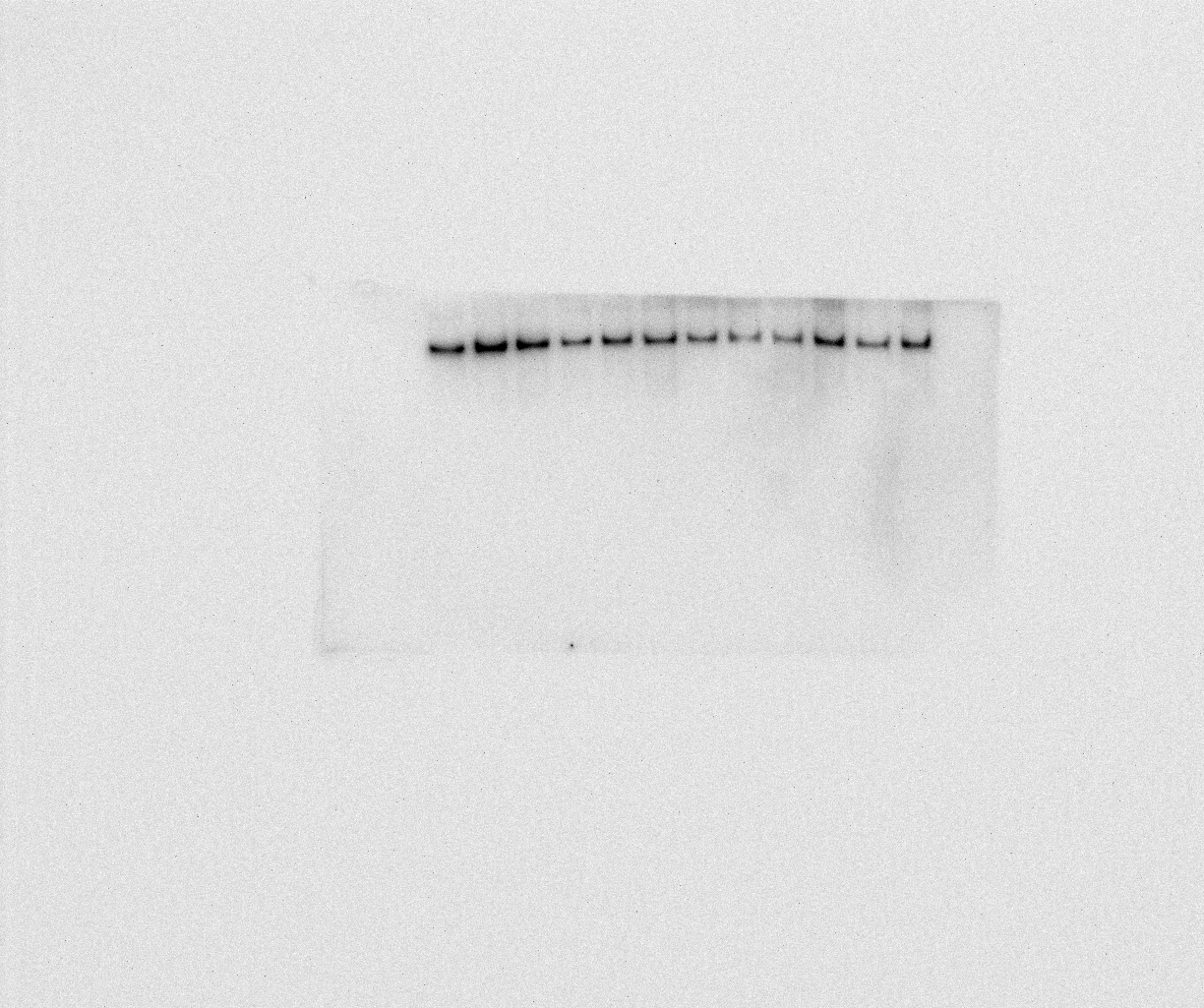

Supplement: Figure 6—source data 2. [file elife-98631-fig6-data2.zip › Figure 6 /6A FMR95xG.tif]

Images corresponding to **Figure 6 – figure supplement 1**:

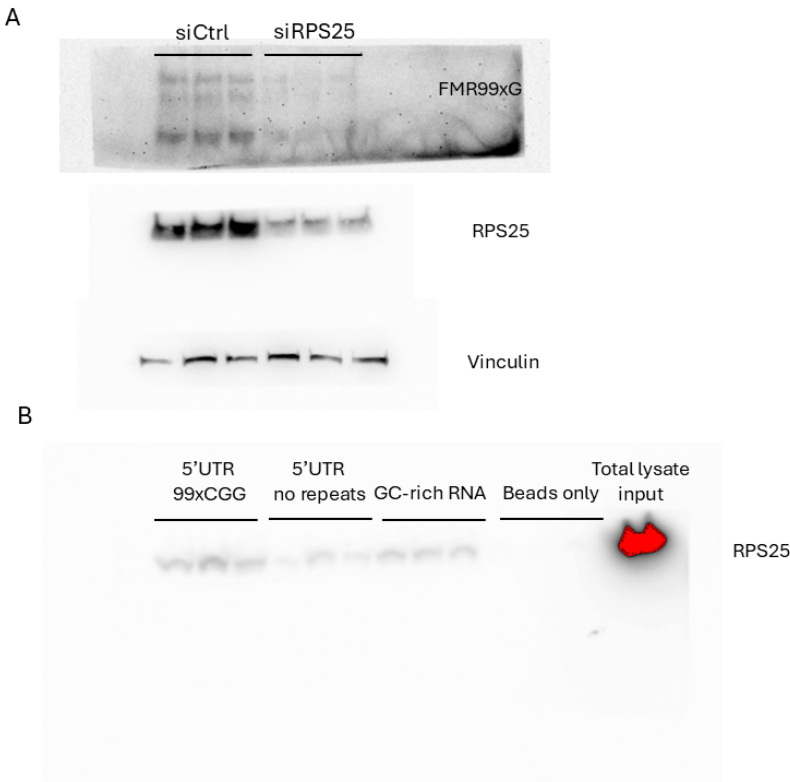

Supplement: Figure 6—figure supplement 1—source data 1. [file elife-98631-fig6-figsupp1-data1.zip › Figure 6 - figure supplement 1 - source data 1.pdf]

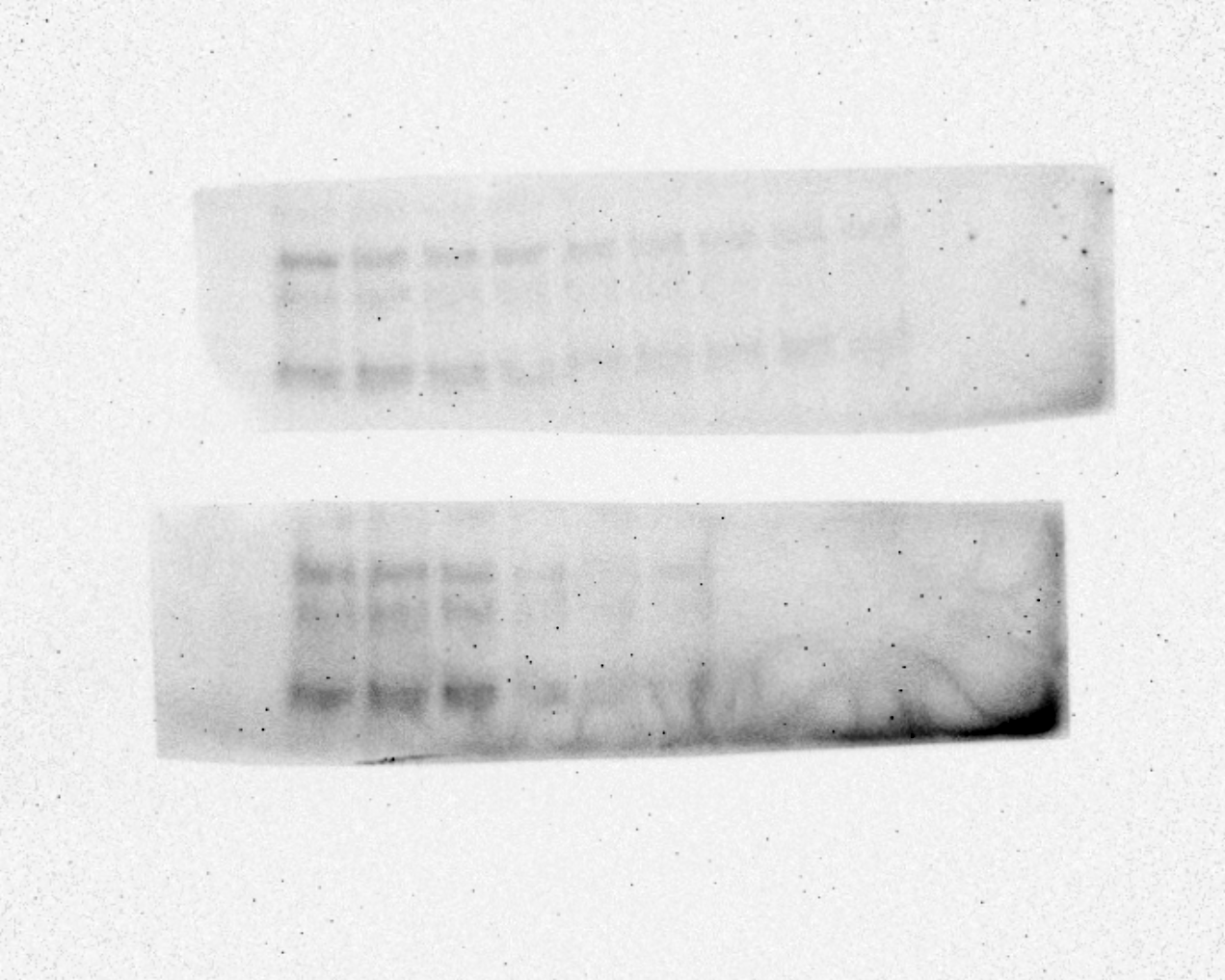

Supplement: Figure 6—figure supplement 1—source data 2. [file elife-98631-fig6-figsupp1-data2.zip › SFig6A FMR99xG.tif]

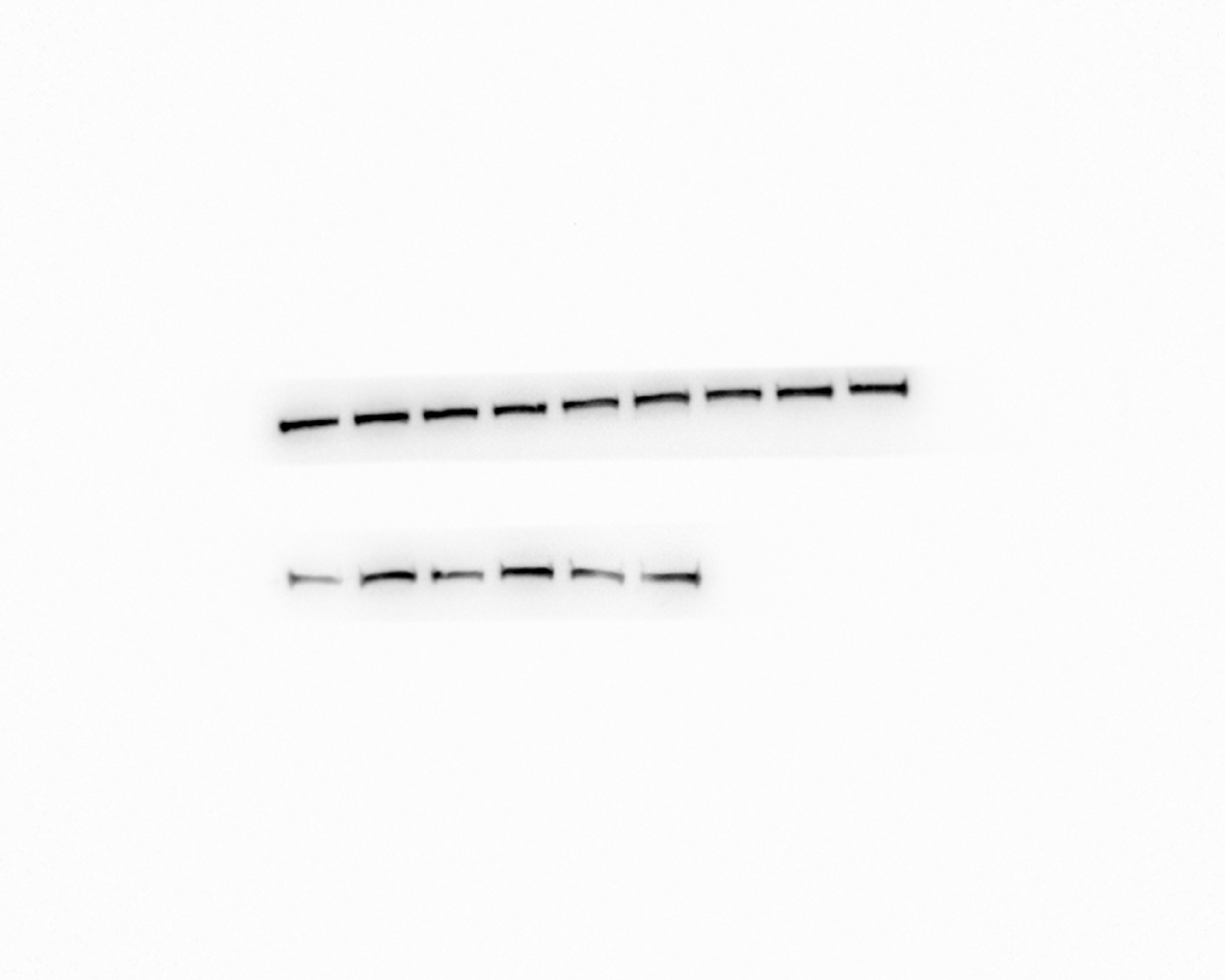

Supplement: Figure 6—figure supplement 1—source data 2. [file elife-98631-fig6-figsupp1-data2.zip › SFig6A Vinculin.tif]

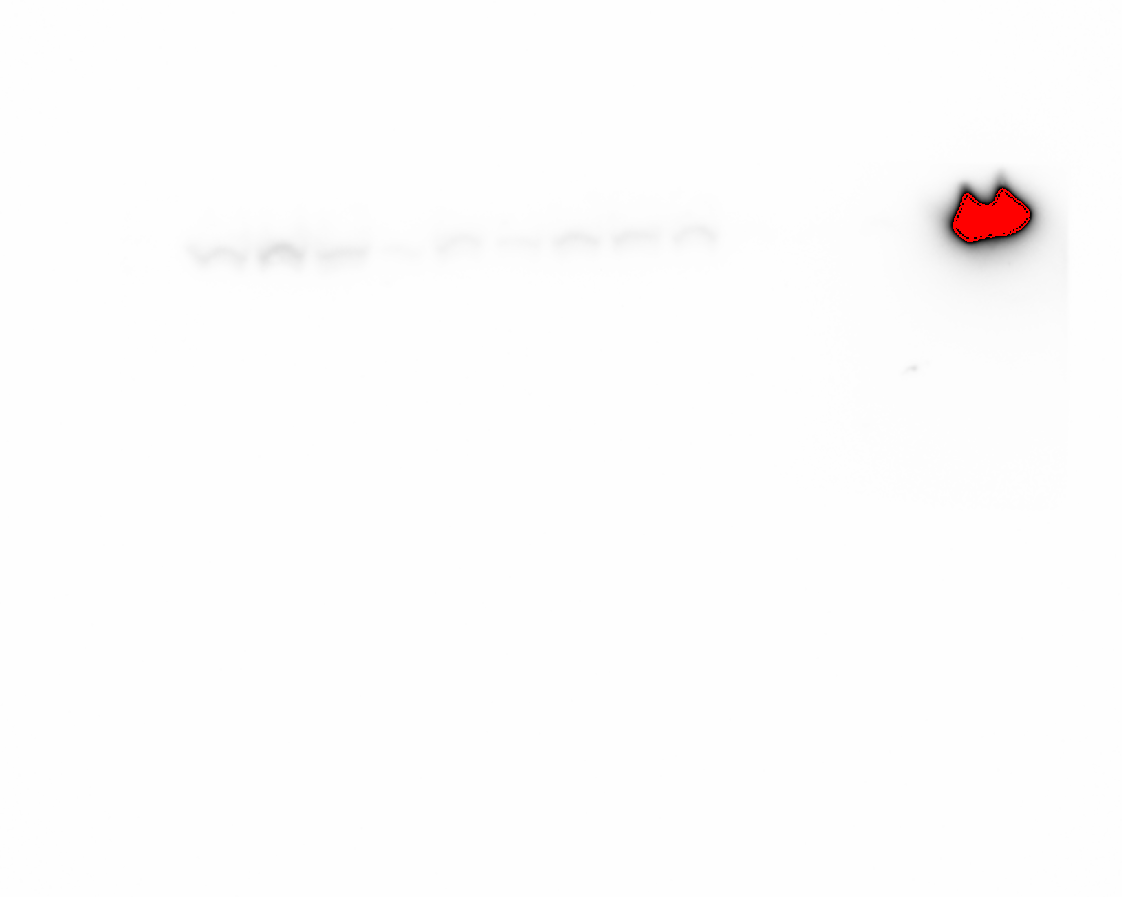

Supplement: Figure 6—figure supplement 1—source data 2. [file elife-98631-fig6-figsupp1-data2.zip › SFig6B RPS25.tif]

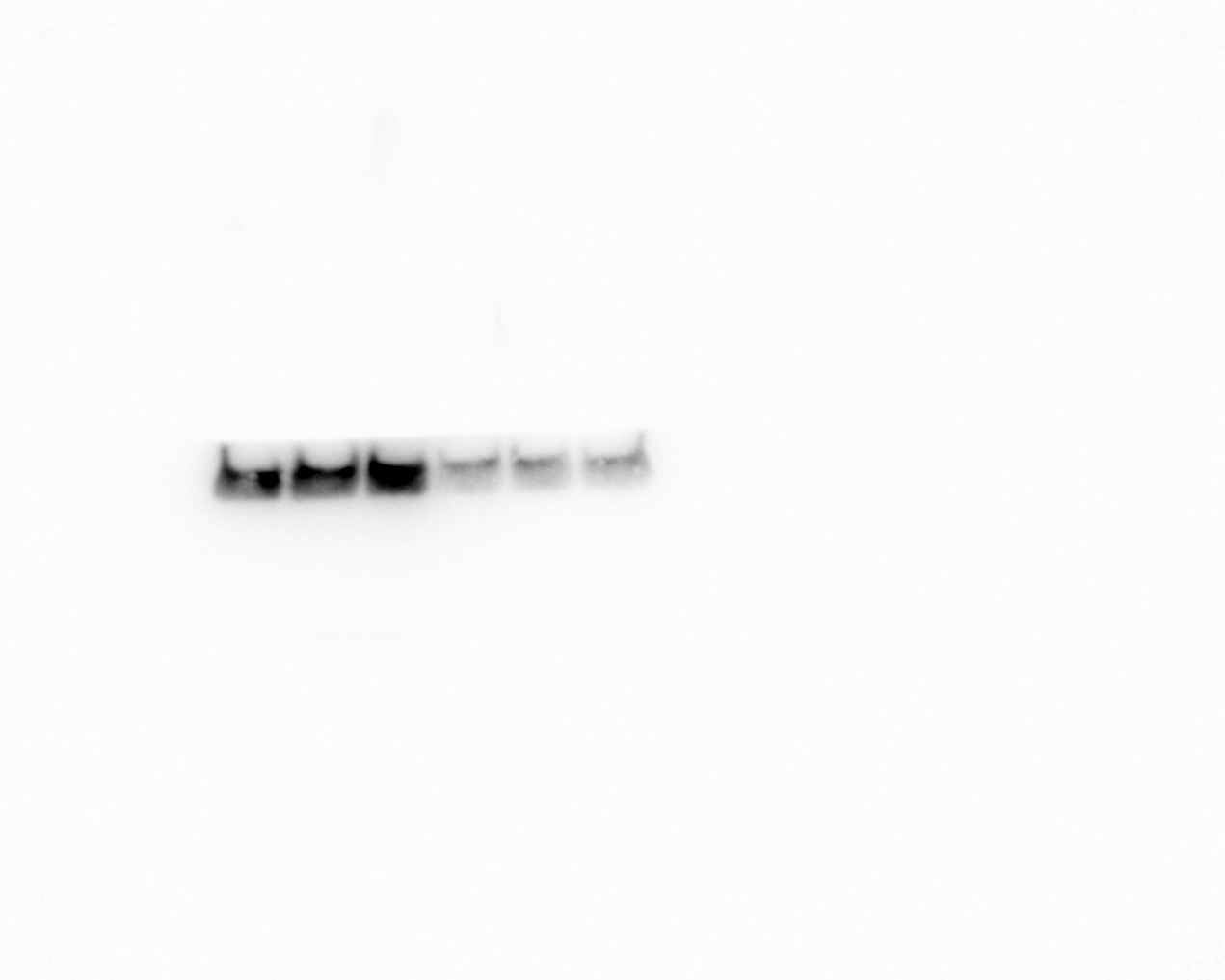

Supplement: Figure 6—figure supplement 1—source data 2. [file elife-98631-fig6-figsupp1-data2.zip › SFig5A RPS25.tif]
